# Response of Plants to Water Stress: A Meta-Analysis

**DOI:** 10.3389/fpls.2020.00978

**Published:** 2020-06-26

**Authors:** Yuan Sun, Cuiting Wang, Han Y. H. Chen, Honghua Ruan

**Affiliations:** ^1^ College of Biology and the Environment, Co-Innovation Center for Sustainable Forestry in Southern China, Nanjing Forestry University, Nanjing, China; ^2^ Faculty of Natural Resource Management, Lakehead University, Thunder Bay, ON, Canada

**Keywords:** water stress, plants, meta-analysis, reactive oxygen species, drought adaption

## Abstract

Plants are key to the functionality of many ecosystem processes. The duration and intensity of water stress are anticipated to increase in the future; however, a detailed elucidation of the responses of plants to water stress remains incomplete. For this study, we present a meta-analysis derived from the 1,301 paired observations of 84 studies to evaluate the responses of plants to water stress. The results revealed that although water stress inhibited plant growth and photosynthesis, it increased reactive oxygen species (ROS), plasma membrane permeability, enzymatic antioxidants, and non-enzymatic antioxidants. Importantly, these responses generally increased with the intensity and duration of water stress, with a more pronounced decrease in ROS anticipated over time. Our findings suggested that the overproduction of ROS was the primary mechanism behind the responses of plants to water stress, where plants appeared to acclimatize to water stress, to some extent, over time. Our synthesis provides a framework for better understanding the responses and mechanisms of plants under drought conditions.

## Introduction

Drought is expected to continuously and significantly increase by the end of this century (Choat et al., 2012; Bu et al., 2018; Sun et al., 2020). Water stress is problematic for plant growth and development (McDowell et al., 2011), as it limits access to the resources required for photosynthesis due to stomatal closure and the reduction of internal water transport (Breda et al., 2006). As such, water stress impairs normal plant functionality and further induces morphological, physiological, and biochemical changes to compensate for water limitations (Mitchell et al., 2013; Lee et al., 2016). Understanding the detailed patterns and mechanisms of responses by plants to water stress is central to predicting future plant functionality and resilience in the face of increasingly frequent drought episodes.

The impacts of water stress on plant growth, physiology, and biochemistry are well documented, and numerous individual studies have examined the roles of plant physiological indices as relates to their tolerance to water stress (van der Molen et al., 2011; Zwicke et al., 2015). For plants, water limitations lead to the overproduction of reactive oxygen species (ROS), such as hydrogen peroxide (H_2_O_2_), and superoxide anion radicals (O2̄·) which results in growth inhibition (Wallace et al., 2016), decreases in photosynthetic functions (Deeba et al., 2012), lipid peroxidation, and the higher frequency of programmed cell death processes (Gill and Tuteja, 2010). However, to adapt to water stress, plants have evolved many acclimation mechanisms, including osmotic adjustment and antioxidant defense systems, which enhances their capacity to grow and develop under drought conditions (Fu and Huang, 2001; Khaleghi et al., 2019). Under water stress conditions, soluble sugars and proline accumulate to serve as osmolytes in various plants, assist in membrane protein stabilization, and ultimately increase plant resistance against water stress (Ashraf and Foolad, 2007; Gomes et al., 2010; Per et al., 2017). Further, ROS scavenging enzymatic antioxidants, such as superoxide dismutase (SOD), peroxidase (POD), catalase (CAT), glutathione reductase (GR), and ascorbate peroxidase (APX) can be activated to clear these excessive ROS (Gill and Tuteja, 2010). Modifications in the activities of these enzymes are likely the primary path in plants for tolerating water stress (Nikoleta-Kleio et al., 2020).

The challenge remains to comprehensively address how various plants respond to water stress, as this can vary considerably (Yuan and Chen, 2015; Schneider et al., 2018). For example, the ROS in leaves may increase (Tang et al., 2017) or decrease (Saglam et al., 2011) under water stress. Similarly, water stress can enhance (Sedaghat et al., 2017) or depress (Zhang et al., 2017) the SOD activities of plants. Previous studies have revealed that the performance of plant responses to water stress may decrease with experimental intensity and duration (Schneider et al., 2018), and vary between different plant species and tissues (Mirzaee et al., 2013; Lum et al., 2014). Therefore, it was necessary to conduct a systematic analysis to summarize the responses of plants under water stress.

The meta-analysis is a statistical methodology for the synthesis of results across multiple studies to attain an overall understanding of a given problem (Gurevitch et al., 2018). A recent meta-analysis has specifically addressed the responses of plants to drought stress (Dong et al., 2017); however it focused on the physiological indices (i.e., plant height, proline, electrolyte leakage, and root length) associated with transcription factors (C-repeat/dehydration-responsive element-binding proteins) that play important roles in plant response to environmental perturbations. Here we focus on the responses of ROS and enzymatic antioxidants (SOD, POD, CAT, GR, and APX), which represent the defense mechanisms of plants under abiotic stresses (Gill and Tuteja, 2010; Sun et al., 2019a). For this study, we established a global dataset by retrieving published papers to January 2020, including 1,301 water-stress experiments from 84 papers (**Table S1**). Our objectives were to explore the general response patterns of plants to water stress, with the aim of providing reliable physiological indices for the screening of drought-resistant species in the future.

## Materials and Methods

### Data Collection

The database utilized in this meta-analysis was collected from peer-reviewed publications (**Table S1**) *via* the Web of Science and Google Scholar, prior to February 2020. The publication screening process is provided in **Figure S1**. Our search terms were “water stress” or “water reduction”, or “drought” and “plant”. The following criteria were applied for this investigation: (1) water stress and control groups began under the same abiotic and biotic conditions. (2) If the experiment included additional treatments, data were selected from the control and water stress groups only. (3) Water stress in these experiments was implemented through the direct manipulation of the soil moisture content in controlled-environment facilities (pot experiments). (4) The water stress duration was clearly reported. (5) The sample sizes and means for the control and treatment groups were directly reported or could be extracted using WebPlotDigitizer (Burda et al., 2017). Measurements from different plant species, water stress intensities, and experimental durations within a single study were considered to be distinct observations. Our final dataset included 1,301 paired observations from 84 primary articles (**Table S2**).

We examined the responses of eighteen indices to drought, including abscisic acid (ABA), ascorbate peroxidase (APX), ascorbate (AsA), carotenoid (Car), CAT, chlorophyll (Chl), dry weight, electrolyte leakage (EL), maximal efficiency of PSII photochemistry (Fv/Fm), glutathione reductase (GR), malondialdehyde (MDA), POD, proline, protein, photochemical quenching coefficient (qP), ROS, SOD, and soluble sugar. These indices were grouped into plant growth (dry weight and protein), photosynthetic characteristics (Chl, Fv/Fm, and qP); plasma membrane permeability (ROS, MDA, and EL), enzymatic antioxidants (APX, GR, CAT, POD, and SOD) and non-enzymatic antioxidants (ABA, AsA, proline, Car, and soluble sugar) based on the morphology, physiology, and functionalities of plants (Gill and Tuteja, 2010).

We collected several independent variables that might affect the responses of plants to drought. Plant tissues were classified as whole plant, leaf, shoots, and roots. Water stress intensity was calculated as the proportional reduction in soil moisture (reduced soil moisture under water stress treatment/soil moisture in the control groups), and the experimental duration was the number of days since its onset. In our dataset, the median water stress intensity was 0.52 and range of 0.05–0.88, whereas the median experimental duration was 36 d and ranged of 1–365 d.

### Statistical Analyses

We employed natural log response ratios (lnRR) as effect sizes (Hedges et al., 1999) to estimate the magnitude of the treatment effect. The lnRR was calculated as ln (X_i_/X_c_) = lnX_i_ − lnX_c_, where X_i_ and X_c_ are the mean values for the water stress and control groups, respectively. The lnRR was weighted by the reciprocal of sampling variance, which was calculated as ln [(1/n_i_) × (S_i_/X_i_)^2^ + (1/n_c_) × (S_c_/X_c_)^2^] using the R package *metafor* 2.1.0 (Viechtbauer, 2010), where S_i_ and S_c_ represent the standard deviations of the water stress and control groups, respectively, with n_i_ and n_c_ as sample sizes. In instances where the standard deviations (SD) were not reported (86 observations) we imputed them using the “Bracken 1992” method (Benitez-Lopez et al., 2017; Sun et al., 2019b) with *metagear* (Lajeunesse, 2016).

For each of the plant physiological indices, we used the following linear mixed-effect model to test whether the mean lnRR differed from zero:

$$\text{lnRR}=\beta_{0}+\beta_{1}\text{ln}(\text{I})+\beta_{2}\text{ln}(\text{D})+\pi_{study}+\varepsilon$$

where I and D represent the water stress intensity and experimental duration. *β_n_*, *π_study_*, and ε are the coefficients to be calculated, the random effect factor of “study”, and sampling error, respectively. We applied linear mixed-effects models using the restricted maximum likelihood estimation with the *lme4* package (Bates et al., 2015). Continuous predictors including water stress intensity and experimental duration in Equation (1) were scaled (observed minus mean and divided by one SD). To examine the linearity assumption between dependent and independent variables, we compared the logarithmic and linear functions for I and D and found that the logarithmic functions for I and D resulted in lower, or similar, Akaike information criterion (AIC) values (**Table S3**). For consistency, we analyzed variables with Equation (1). Accordingly, we also employed Equation (1) to test whether the mean lnRR of individual plant performance differed from zero. Because both predictors [ln(I) and ln(D)] were scaled, β_0_ represented the mean lnRR for the means of the predictors.

To examine whether the lnRR of physiological indices were altered with plant tissues, we tested the effects of plant tissues on the lnRR by adding the plant tissue terms to Equation (1). For ease of interpretation, we transformed the lnRR and its corresponding confidence interval (CI) using [exp (lnRR) − 1] × 100%. Further, linear-regressions were employed to examine the correlations of plant physiological indices and performances with water stress intensity and experimental duration, respectively. All statistical analyses were performed using R 3.6.0 software (R Development Core Team, 2019).

## Results

**Figure 1:**
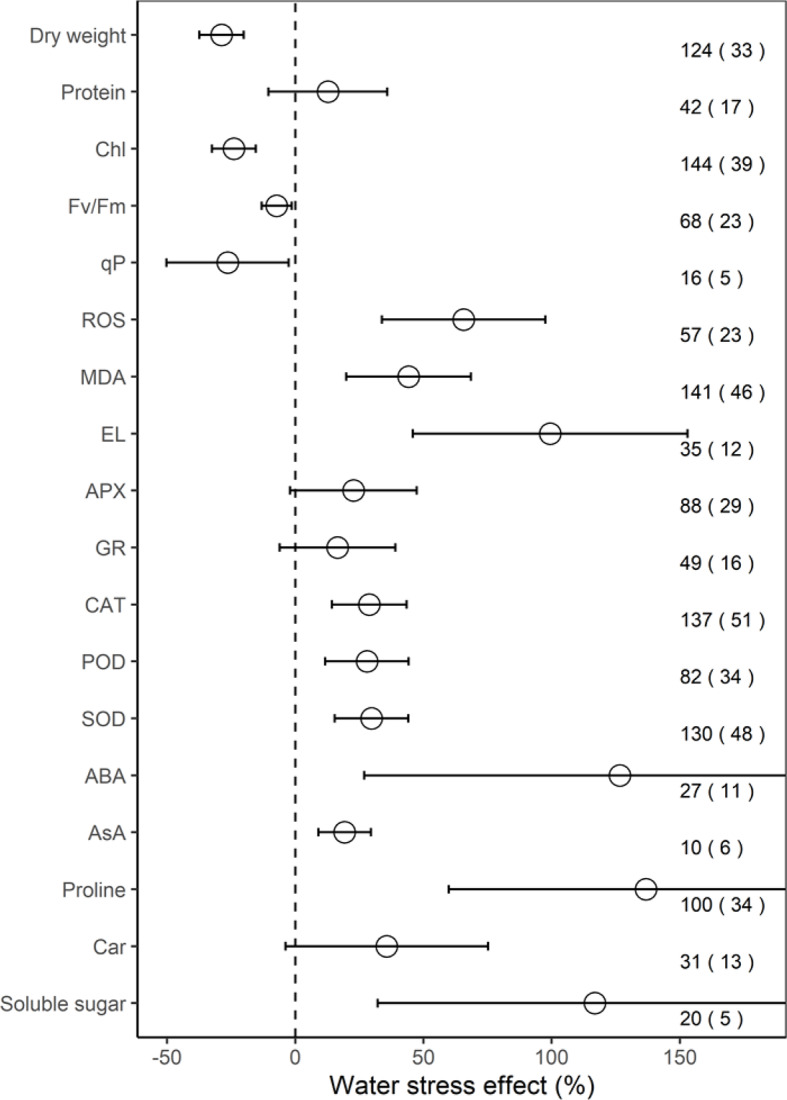
Response of physiological indices to water stress. Values are the means and 95% confidence intervals. The dashed black line represents zero effect size. Numbers without and within parentheses represent the number of observations and studies, respectively. Chl, Fv/Fm, qP, ROS, MDA, EL, APX, GR, CAT, POD, SOD, ABA, AsA, and Car, represent chlorophyll, maximal efficiency of PSII photochemistry, photochemical quenching coefficient, reactive oxygen species, malondialdehyde, electrolyte leakage, ascorbate peroxidase, glutathione reductase, catalase, peroxidase, superoxide dismutase, abscisic acid, ascorbate, and carotenoid, respectively.

Across all individual studies, the ROS increased significantly, by 65.7% on average (33.8–97.6%, *P* < 0.001), MDA by 44.2% (19.9–68.5%, *P* < 0.01), EL by 99.4% (45.9–153.0%, *P* < 0.01), CAT by 28.8% (14.3–43.4%, *P* < 0.01), POD by 28.0% (11.7–44.2%, *P* < 0.01), SOD by 29.8% (15.4–44.1%, *P* < 0.001), ABA by 126.6% (CI, 26.9–226.3%; *P* = 0.01), AsA by 19.3% (9.1–29.5%; *P* < 0.01), proline by 136.8% (59.9–213.7%, *P* < 0.001), and soluble sugar by 116.9% (32.2–201.5%, *P* = 0.03) under water stress, compared to the mean of the control groups (**Figure 1**). However, on average, water stress significantly (*P* < 0.05) decreased dry weight by 28.8%, Chl by 23.9%, Fv/Fm by 13.1%, and qP by 26.4%, but had no significant impacts on protein, APX, GR, and Car (all *P* > 0.05).

**Figure 2:**
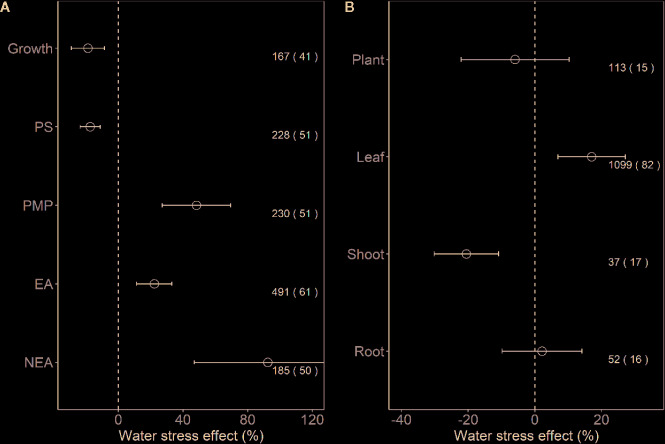
Response of plant performance **(A)** and tissues **(B)** to water stress. Values are means and 95% confidence intervals. Dashed black line represents zero effect size. Numbers without and within parentheses represent the number of observations and studies, respectively. PS, PMP, EA, and NEA represent photosynthesis, plasma membrane permeability, enzymatic antioxidants, and non-enzymatic antioxidants, respectively.

We found that the effect sizes for PMP, EA, and NEA increased significantly under water stress (all *P* < 0.001), and the effect size for growth and PS decreased (all *P* < 0.001; **Figure 2A**). Furthermore, for plant tissues tested indices, water stress had positive effects on leaves (17.1%, *P* < 0.01), negative effects on shoots (−20.5%, *P* < 0.001), but no effects on the whole plants and roots (all *P* > 0.05; **Figure 2B**).

**Figure 3:**
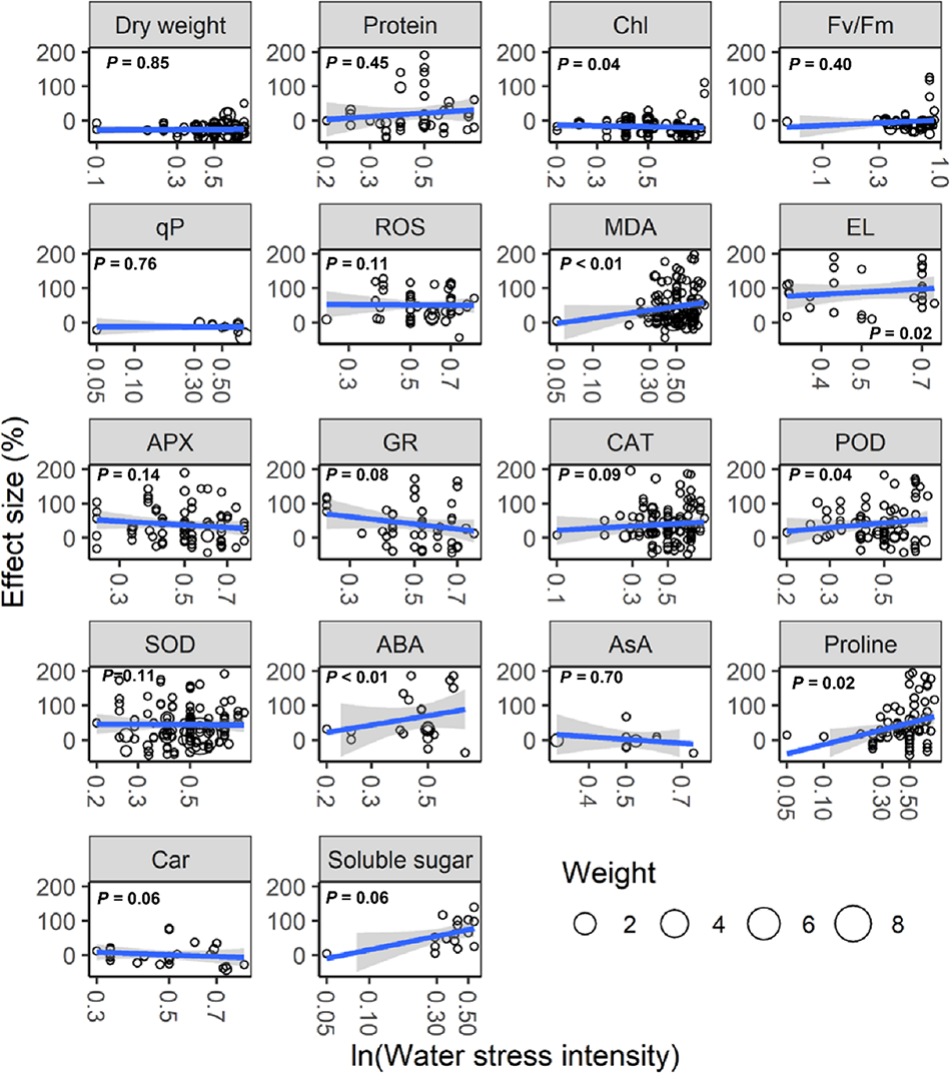
Responses of plant physiological indices to water stress intensity. Linear regressions (blue lines) and their 95% confidence intervals (shaded areas) and corresponding levels of significances (*P* values) are presented. Circle sizes are proportional to the sampling variances. See **Figure 1** for abbreviations.

**Figure 4:**
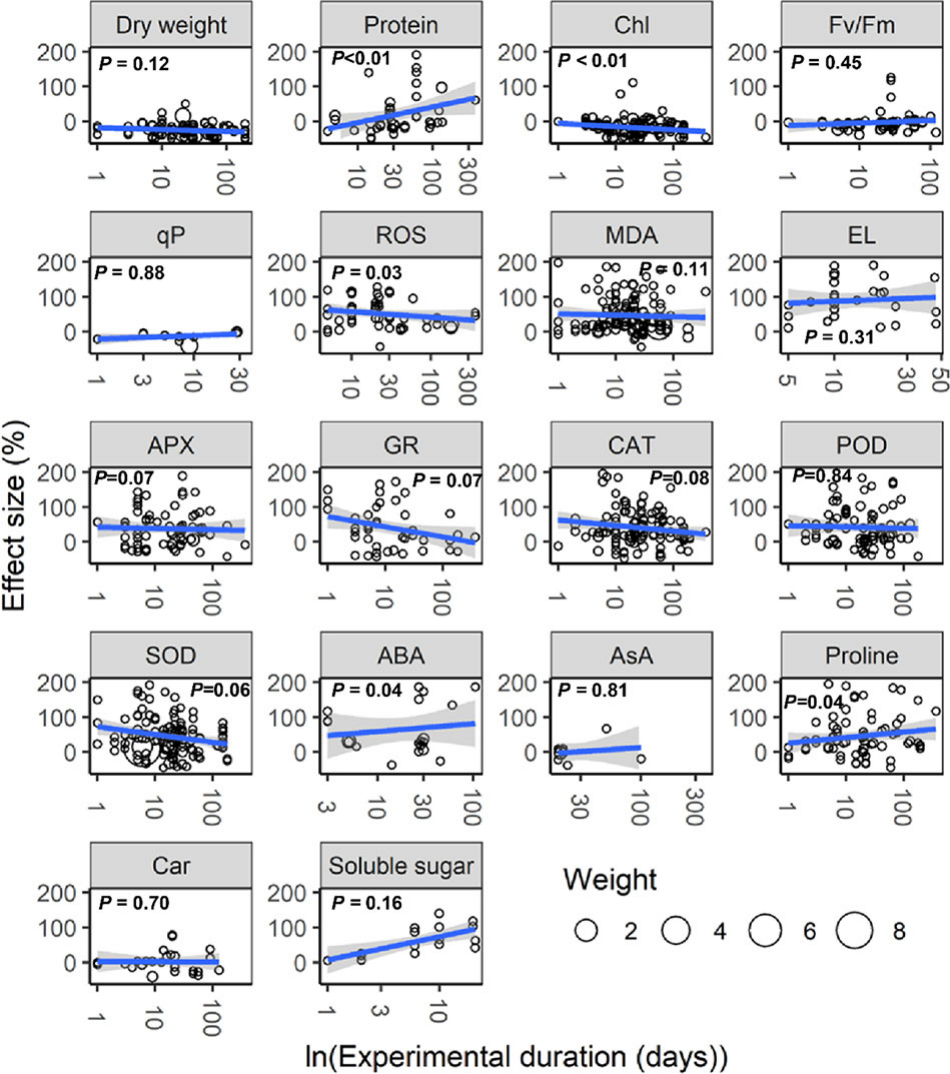
Responses of plant physiological indices to experimental duration. Linear regressions (blue lines) and their 95% confidence intervals (shaded areas) and corresponding levels of significances (*P* values) are presented. Circle sizes are proportional to the sampling variances. See **Figure 1** for abbreviations.

**Figure 5:**
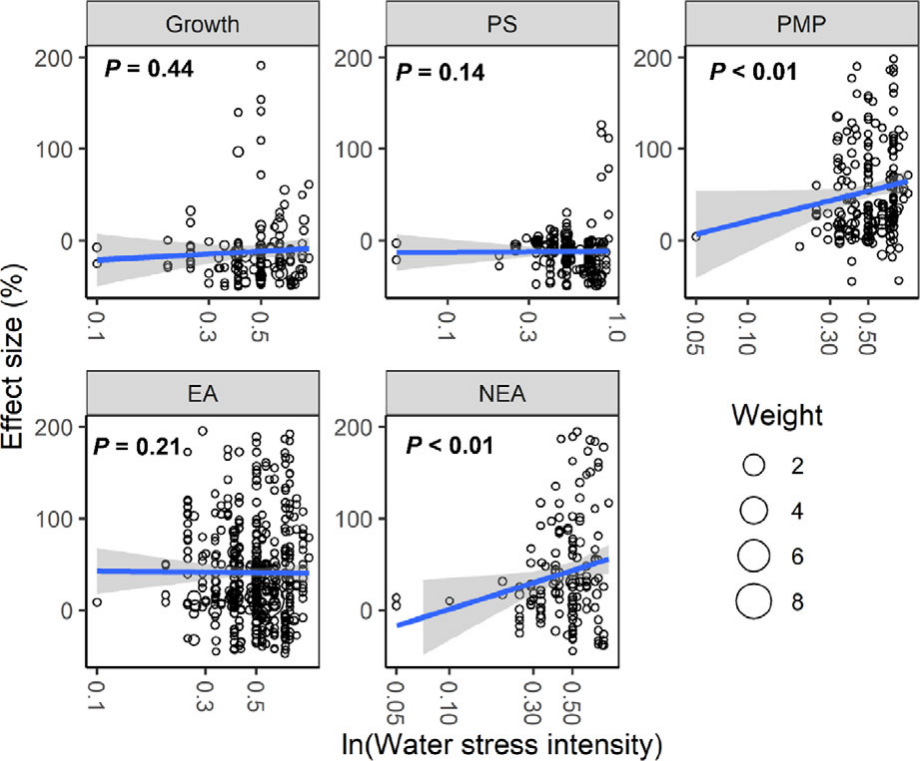
Responses of plant performances to water stress intensity. Linear regressions (blue lines) and their 95% confidence intervals (shaded areas) and corresponding levels of significances (*P* values) are presented. Circle sizes are proportional to the sampling variances. See **Figure 2** for abbreviations.

**Figure 6:**
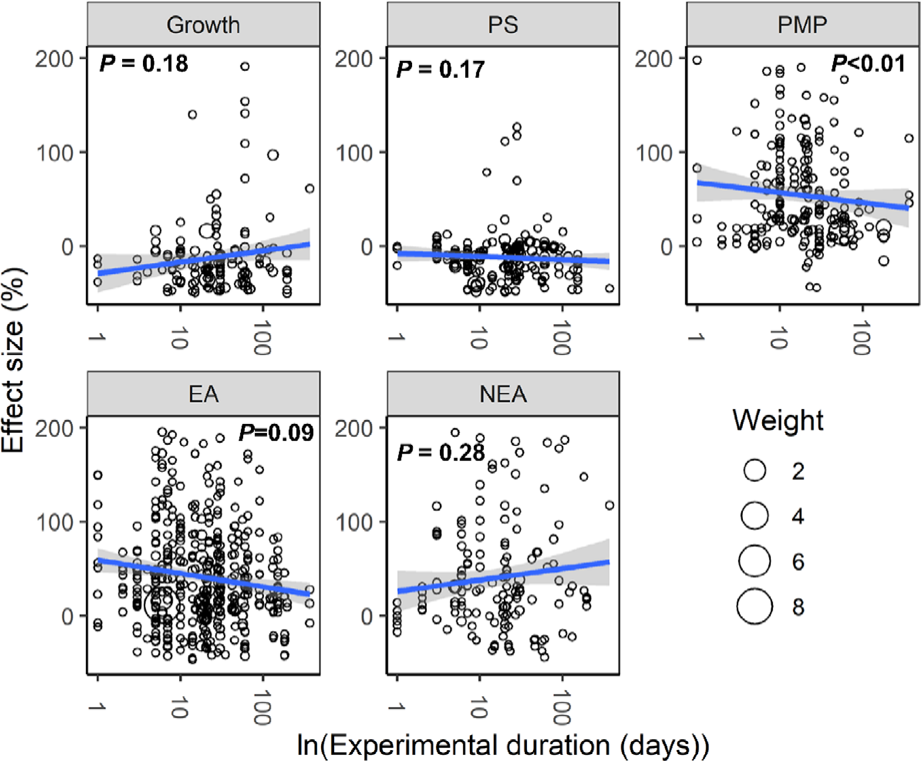
Responses of plant performances to experimental duration. Linear regressions (blue lines) and their 95% confidence intervals (shaded areas) and corresponding levels of significances (*P* values) are presented. Circle sizes are proportional to the sampling variances. See **Figure 2** for abbreviations.

With increasing water stress intensity, the effect size for MDA, EL, POD, ABA, and proline increased significantly (all *P* < 0.05), whereas the effect size for Chl decreased (*P* = 0.04; **Figure 3**). The effect sizes for Chl and ROS decreased significantly with experimental duration (all *P* < 0.05), and the effect size for protein, ABA, and proline increased (all *P* < 0.05; **Figure 4**). The effect sizes for PMP and NEA increased significantly with water stress intensity (all *P* < 0.01; **Figure 5**), and PMP decreased significantly with experimental duration (*P* < 0.01; **Figure 6**).

## Discussion

The meta-analysis for this study, based on 1,301 observations, is the first to integrally examine the responses of various plants to water stress. A consistent and general plant response pattern to water stress was found. These responses were more pronounced with water stress intensity and experimental duration, except for a decrease in ROS over time. Below, we elaborate on the potential mechanisms for the observed patterns and conclude with suggestions for future research.

### Mechanisms Behind Plant Responses

Our analysis revealed that water stress significantly inhibited plant growth and photosynthesis (**Figures 1** and **2**). Further, we found that the negative response of Chl to water stress was more pronounced with increasing intensity and duration (**Figures 3** and **4**). This suggests that Chl is very sensitive to water stress among various types of plants. The probable explanation was that water stress damaged photosynthetic organs and altered leaf structures, thereby reducing the photosynthetic activities of plants and negatively impacting growth (Aranjuelo et al., 2011). The overproduction of ROS accompanied by increasing MDA and EL indicated a malfunction of the plasma membrane (Murray et al., 1989; Bouchemal et al., 2016) and lipid peroxidation (Sun et al., 2019a), respectively. Our meta-analysis demonstrated that water stress significantly increased ROS, MDA, and EL (**Figure 1**), and stimulated PMP (**Figure 2A**), which suggested that membrane damage occurred under water stress. Schneider et al. (2018) reported that high ROS concentrations in plants were extremely toxic to lipids and resulted in oxidative stress. Together, these results indicated that the overproduction of ROS was the primary mechanism of water stress. We also found that MDA and PMP exhibited positive responses to intensity; however, ROS and PMP decreased with duration (**Figures 3**–**6**), which indicated that plants adjusted their growth morphology, and physiological indices to adapt to water stress over time, as reported in previous meta-analyses (He and Dijkstra, 2014). One possible explanation is that plants have evolved a number of strategies to adapt to water stress (see the discussion below) (Anjum et al., 2012; Sperdouli and Moustakas, 2012).

Our study offers new insights into the increase of EA being associated with scavenged ROS under water stress (**Figure 2A**). Although CAT, POD, and SOD activities were higher under water stress than the control, both APX and GR did not show significant responses to water stress (**Figure 1**). This suggested that the EA associated with the Halliwell-Asada pathway may work less efficiently than CAT, POD, and SOD, likely because various enzymes located within different cellular compartments have disparate functions (Mittler, 2002; Gill and Tuteja, 2010; Sun et al., 2019a). Further, the POD activity increased with intensity (**Figure 3**), which can be attributed to the capacity of plants to withstand stress (DaCosta and Huang, 2007) to maintain normal metabolic processes (Schneider et al., 2018). Thus, we recommend that the kinetics involved in the enzymatic responses to water stress should be investigated in future experiments.

Our study revealed higher ABA, AsA, proline, soluble sugar, and NEA (**Figures 1** and **2A**) under water stress. We also found that the positive responses of ABA, proline, and NEA were more pronounced with intensity (**Figures 3** and **5**), which suggested that ABA and proline were sensitive plant physiological indices to water stress. Higher concentrations of ABA served to facilitate an adaptation to water stress (Belkheiri and Mulas, 2013), whereas the increased accumulation of proline was considered to mitigate the adverse effects of ROS (Chen and Dickman, 2005). Interestingly, plant roots were not significantly impacted by water stress (**Figure 2B**). One possible explanation was that increased numbers of root ducts improved the efficacy of water transport, which assisted plants in resisting water stress (Lee et al., 2016).

### Suggestions for Future Experiments

We encountered two important inconsistencies in our meta-analysis across studies. Firstly, only 89 observations of the 1,301 in our dataset studied shoots and roots (**Figure 2B**). Plant shoots and roots are critical for projecting the impacts of drought on the functionalities of plant communities (He and Dijkstra, 2014; Sardans et al., 2017). Therefore, we propose additional water stress experiments to study shoots and roots that incorporate a wide range of physiological indices. Secondly, as with several ecological meta-analyses, we discovered a hemispheric bias in our knowledge of the effects of water stress on plants (Feeley et al., 2017). Most observations were derived from experiments that were performed at a latitude of >19.3° in the Northern Hemisphere (only nine studies were conducted in the Southern Hemisphere) (**Table S2**). Therefore, we are optimistic that our study will motivate new publications in underrepresented regions.

## Conclusion

In conclusion, our meta-analysis, which employed a global empirical dataset for the responses of plants to water stress, revised the previous notion that water stress inhibits plant growth (Schneider et al., 2018). The divergent responses of Chl, ROS, MDA, and EL were partially explained by the inhibition of plant growth. Water stress affected plant performance primarily through the overproduction of ROS, which led to plasma membrane damage. Meanwhile, a variety of physiological indices, i.e., CAT, POD, SOD, ABA, and proline were activated to control the levels of cellular ROS to compensate for water stress. These indices above were evaluated to facilitate screening for drought-resistant species. Further, the effects of water stress were observed to be more pronounced with intensity and duration, except for a decrease in ROS with water stress duration. Therefore, imbuing plants with the capacity to scavenge excessive ROS will be useful in the future to enhance their endurance during drought events.

## Data Availability Statement

The datasets generated for this study are available on request to the corresponding author.

## Author Contributions

YS and HR conceived the study. YS performed the meta-analysis, and wrote the first draft. CW helped to collect and process the data. HC and HR contributed to the interpretation of data and manuscript revisions.

## Funding

This work was supported by the National Key Research and Development Program of China (No. 2016YFD0600204) and the Priority Academic Program Development of Jiangsu Higher Education Institutions (PAPD).

## Conflict of Interest

The authors declare that the research was conducted in the absence of any commercial or financial relationships that could be construed as a potential conflict of interest.

## Abbreviations

ABA, abscisic acid; APX, ascorbate peroxidase; AsA, ascorbate; Car, carotenoid; CAT, catalase; Chl, chlorophyll; EA, enzymatic antioxidants; EL, electrolyte leakage; Fv/Fm, maximal efficiency of PSII photochemistry; GR, glutathione reductase; MDA, malondialdehyde; NEA, non-enzymatic antioxidants; PMP, plasma membrane permeability; POD, peroxidase; PS, photosynthesis; qP, photochemical quenching coefficient; ROS, reactive oxygen species; SD, standard deviation; SOD, superoxide dismutase.
