# Supplementary material for: Response of Plants to Water Stress: A Meta-Analysis
Source: Front Plant Sci. 2020 Jun 26;11:978. doi: 10.3389/fpls.2020.00978 (PMC7333662; doi:10.3389/fpls.2020.00978)
Supplement: Supplementary file 1 [file DataSheet_1.docx]

Supplementary Information

**Response of plants to water stress: a meta-analysis**

Yuan Sun, Cuiting Wang, Han Y.H. Chen, Honghua Ruan*

Honghua Ruan

Email: hhruan@njfu.edu.cn

**This file includes:**

Fig S1;

Tables S1 to S3


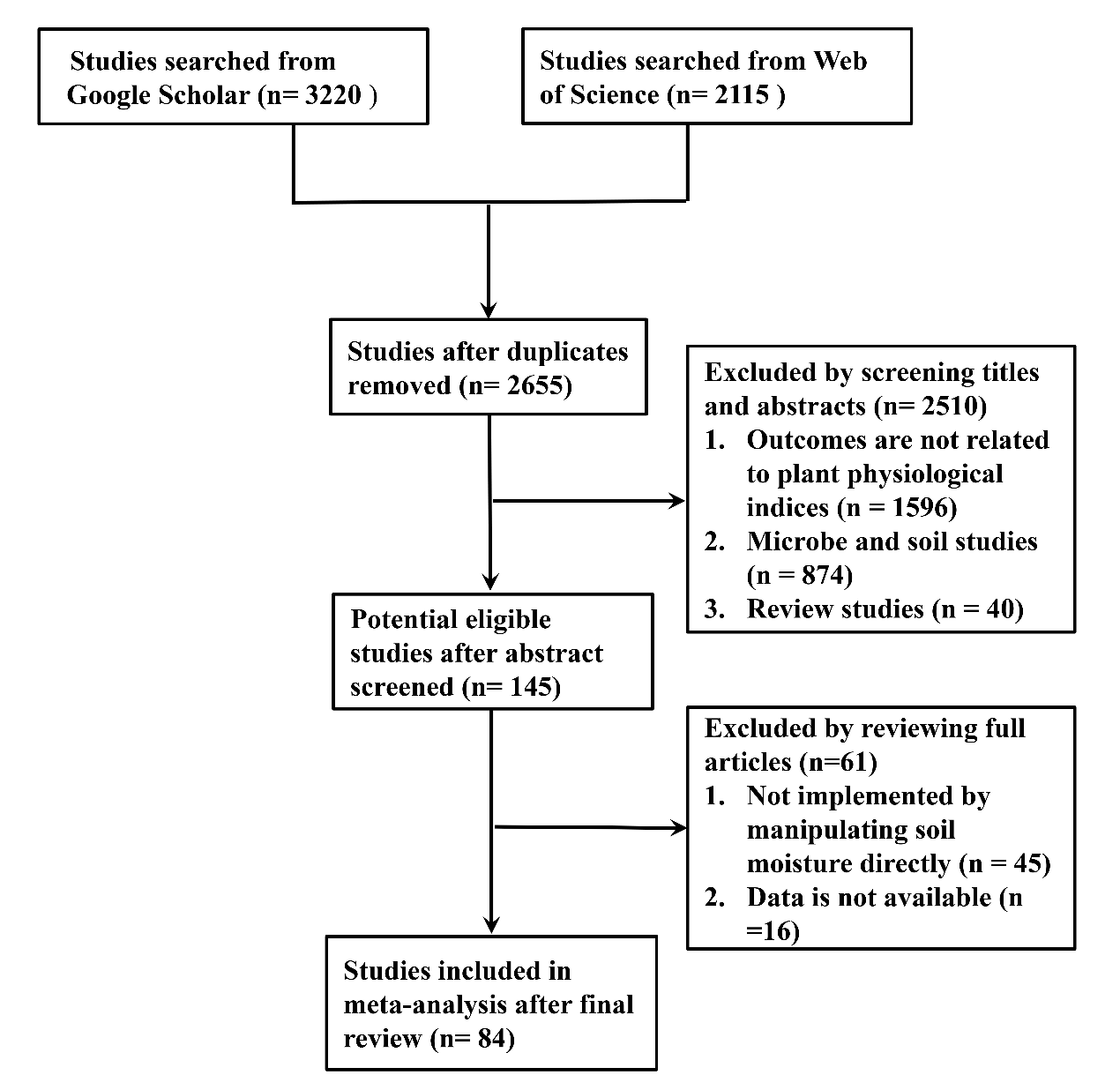


## **Figure S1.** Workflow diagram showing the procedure for selecting publications.

## **Table S1.** Bibliography for studies included in the meta-analysis.

| NO. | Publication |
| --- | --- |

1. Abedi, T., Pakniyat, H., 2010. Antioxidant Enzyme Changes in Response to Drought Stress in Ten Cultivars of Oilseed Rape (Brassica napus L.). Czech Academy of Agricultural Sciences 46, 27-34.
2. Abid, G., M’hamdi, M., Mingeot, D., Aouida, M., Aroua, I., Muhovski, Y., Sassi, K., Souissi, F., Mannai, K., Jebara, M., 2016. Effect of drought stress on chlorophyll fluorescence, antioxidant enzyme activities and gene expression patterns in faba bean (Vicia faba L.). Archives of Agronomy and Soil Science 63, 536-552.
3. Abuelsoud, W., Papenbrock, J., 2019. Drought differentially elicits antioxidant defense systems in two genotypes of Euphorbia tirucalli. Flora 259, 151460.
4. Agami, R.A., Ghramh, H.A., Hashem, M., 2017. Seed inoculation with Azospirillum lipoferum alleviates the adverse effects of drought stress on wheat plants. Journal of applied botany and food quality-angewandte botanik 90, 165-173.
5. Ashraf, M.A., Rasheed, R., Hussain, I., Iqbal, M., Haider, M.Z., Parveen, S., Sajid, M.A., 2014. Hydrogen peroxide modulates antioxidant system and nutrient relation in maize (Zea maysL.) under water-deficit conditions. Archives of Agronomy and Soil Science 61, 507-523.
6. Bandeppa, S., Paul, S., Thakur, J.K., Chandrashekar, N., Umesh, D.K., Aggarwal, C., Asha, A.D., 2019. Antioxidant, physiological and biochemical responses of drought susceptible and drought tolerant mustard (Brassica juncea L) genotypes to rhizobacterial inoculation under water deficit stress. Plant physiology and biochemistry: PPB 143, 19-28.
7. Bano, A., Ullah, F., Nosheen, A., 2012. Role of abscisic acid and drought stress on the activities of antioxidant enzymes in wheat. Plant Soil Environment 58, 181-185.
8. Baroowa, B., Gogoi, N., Farooq, M., 2016. Changes in physiological, biochemical and antioxidant enzyme activities of green gram (Vigna radiata L.) genotypes under drought. Acta Physiologiae Plantarum 38.
9. Batista, P.F., Muller, C., Merchant, A., Fuentes, D., de Oliveira Silva Filho, R., da Silva, F.B., Costa, A.C., 2019. Biochemical and physiological impacts of zinc sulphate, potassium phosphite and hydrogen sulphide in mitigating stress conditions in soybean. Physiologia plantarum.
10. Bayat, H., Moghadam, A.N., 2019. Drought effects on growth, water status, proline content and antioxidant system in three Salvia nemorosa L. cultivars. Acta Physiologiae Plantarum 41.
11. Belkheiri, O., Mulas, M., 2013. Effect of water stress on growth, water use efficiency and gas exchange as related to osmotic adjustment of two halophytes Atriplex spp. Functional Plant Biology 40, 466-474.
12. Borjas‐Ventura, R., Ferraudo, A.S., Martínez, C.A., Gratão, P.L., 2019. Global warming: Antioxidant responses to deal with drought and elevated temperature in Stylosanthes capitata, a forage legume. Journal of Agronomy and Crop Science 206, 13-27.
13. Bouchemal, K., Bouldjadj, R., Belbekri, M.N., Ykhlef, N., Djekoun, A., 2016. Differences in antioxidant enzyme activities and oxidative markers in ten wheat (Triticum durumDesf.) genotypes in response to drought, heat and paraquat stress. Archives of Agronomy and Soil Science 63, 710-722.
14. Brossa, R., Pinto-Marijuan, M., Francisco, R., Lopez-Carbonell, M., Chaves, M.M., Alegre, L., 2015. Redox proteomics and physiological responses in Cistus albidus shrubs subjected to long-term summer drought followed by recovery. Planta 241, 803-822.
15. Campos, C.N., Ávila, R.G., de Souza, K.R.D., Azevedo, L.M., Alves, J.D., 2019. Melatonin reduces oxidative stress and promotes drought tolerance in young Coffea arabica L. plants. Agricultural Water Management 211, 37-47.
16. Chen, D., Wang, S., Cao, B., Cao, D., Leng, G., Li, H., Yin, L., Shan, L., Deng, X., 2015. Genotypic Variation in Growth and Physiological Response to Drought Stress and Re-Watering Reveals the Critical Role of Recovery in Drought Adaptation in Maize Seedlings. Frontiers in plant science 6, 1241.
17. Cotrozzi, L., Remorini, D., Pellegrini, E., Landi, M., Massai, R., Nali, C., Guidi, L., Lorenzini, G., 2016. Variations in physiological and biochemical traits of oak seedlings grown under drought and ozone stress. Physiologia plantarum 157, 69-84.
18. DaCosta, M., Huang, B., 2007. Changes in antioxidant enzyme activities and lipid peroxidation for Bentgrass species in response to drought stress. Journal of the american society for horticultural science 132, 319-326.
19. Daniel, E., 1997. Effects of drought on photosynthesis and on the thermotolerance of photosystem II in seedlings of cedar (Cedrus atlantica and C. libani). Journal of Experimental Botany 48, 18365-11841.
20. de Oliveira, H.O., de Castro, G.L.S., Correa, L.O., Silvestre, W.V.D., do Nascimento, S.V., da Silva Valadares, R.B., de Oliveira, G.C., Santos, R.I.N., Festucci-Buselli, R.A., Pinheiro, H.A., 2019. Coupling physiological analysis with proteomic profile to understand the photosynthetic responses of young Euterpe oleracea palms to drought. Photosynthesis research 140, 189-205.
21. Ditmarova, L., Kurjak, D., Palmroth, S., Kmet, J., Strelcova, K., 2010. Physiological responses of Norway spruce (Picea abies) seedlings to drought stress. Tree physiology 30, 205-213.
22. Du, Y.-L., Wang, Z.-Y., Fan, J.-W., Turner, N.C., Wang, T., Li, F.-M., 2012. Aminobutyric acid increases abscisic acid accumulation and desiccation tolerance and decreases water use but fails to improve grain yield in two spring wheat cultivars under soil drying. Journal of Experimental Botany 63, 4849-4860.
23. E., G.P., 1984. Effect of Water Stress on the Chloroplast Antioxidant System. Plant Physiology. 76, 615-621.
24. Fang, X.W., Turner, N.C., Li, F.M., Li, W.J., Guo, X.S., 2011. Caragana korshinskii seedlings maintain positive photosynthesis during short-term, severe drought stress. Photosynthetica 49, 603-609.
25. Gao, S., Wang, Y., Yu, S., Huang, Y., Liu, H., Chen, W., He, X., 2020. Effects of drought stress on growth, physiology and secondary metabolites of Two Adonis species in Northeast China. Scientia Horticulturae 259, 108795.
26. Ghafar, M.A., Akram, N.A., Ashraf, M., Ashraf, M.Y., Sadiq, M., 2019. Thiamin-induced variations in oxidative defense processes in white clover ( Trifolium repens L.) under water deficit stress. Turkish Journal of Botany 43, 58-66.
27. Ghanbary, E., Kouchaksaraei, M.T., Guidi, L., Mirabolfathy, M., Etemad, V., Sanavi, S.A.M.M., Struve, D., 2018. Change in biochemical parameters of Persian oak (Quercus brantii Lindl.) seedlings inoculated by pathogens of charcoal disease under water deficit conditions. Trees 32, 1595-1608.
28. Gholami, M., Rahemi, M., Kholdebarin, B., Rastegar, S., 2012. Biochemical responses in leaves of four fig cultivars subjected to water stress and recovery. Scientia Horticulturae 148, 109-117.
29. Gokmen, E., Ceyhan, E., 2015. Effects of Drought Stress on Growth Parameters, Enzyme Activities and Proline Content in Chickpea Genotypes. Bangladesh journal of botany 44, 177-183.
30. Hameed, A., Goher, M., Iqbal, N., 2012. Drought induced programmed cell death and associated changes in antioxidants, proteases, and lipid peroxidation in wheat leaves. Biologia Plantarum 57, 370-374.
31. Hao, S., Cao, H., Wang, H., Pan, X., 2019. The physiological responses of tomato to water stress and re-water in different growth periods. Scientia Horticulturae 249, 143-154.
32. He, C., Wang, W., Hou, J., 2019. Plant Growth and Soil Microbial Impacts of Enhancing Licorice With Inoculating Dark Septate Endophytes Under Drought Stress. Frontiers in microbiology 10, 2277.
33. Hosseini, S.M., Hasanloo, T., Mohammadi, S., 2015. Physiological characteristics, antioxidant enzyme activities, and gene expression in 2 spring canola (Brassica napus L.) cultivars under drought stress conditions. Turkish Journal of Agriculture and Forestry 39, 413-420.
34. Hu, W.H., Xiao, Y.A., Zeng, J.J., Hu, X.H., 2010. Photosynthesis, respiration and antioxidant enzymes in pepper leaves under drought and heat stresses. Biologia Plantarum 54, 761-765.
35. Husen, A., 2010. Growth Characteristics, Physiological and Metabolic Responses of Teak (Tectona Grandis Linn. f.) Clones Differing in Rejuvenation Capacity Subjected to Drought Stress. Silvae Genetica 59, 124-136.
36. Huseynova, I.M., Aliyeva, D.R., Mammadov, A., Aliyev, J.A., 2015. Hydrogen peroxide generation and antioxidant enzyme activities in the leaves and roots of wheat cultivars subjected to long-term soil drought stress. Photosynthesis research 125, 279-289.
37. Iqbal, H., Yaning, C., Waqas, M., Rehman, H., Shareef, M., Iqbal, S., 2018. Hydrogen peroxide application improves quinoa performance by affecting physiological and biochemical mechanisms under water-deficit conditions. Journal of Agronomy and Crop Science 204, 541-553.
38. Júnior, S.d.O.M., Endres, L., Silva, J.V., Andrade, J.R.d., 2019. An efficient antioxidant system is associated with lower photosynthesis photoinhibition and greater tolerance to drought in sugarcane cultivars. Bioscience Journal, 691-704.
39. Jafari, S., Hashemi Garmdareh, S.E., Azadegan, B., 2019. Effects of drought stress on morphological, physiological, and biochemical characteristics of stock plant (Matthiola incana L.). Scientia Horticulturae 253, 128-133.
40. Jalil Sheshbahreh, M., Movahhedi Dehnavi, M., Salehi, A., Bahreininejad, B., 2019. Physiological and yield responses of purple coneflower (Echinacea purpurea (L.) Moench) to nitrogen sources at different levels of irrigation. Physiology and molecular biology of plants: an international journal of functional plant biology 25, 177-187.
41. Ji, Y., Zhang, X., Peng, Y., Huang, L., Liang, X., Wang, K., Yin, G., Zhao, X., 2014. Osmolyte accumulation, antioxidant enzyme activities and gene expression patterns in leaves of orchardgrass during drought stress and recovery. Grassland Science 60, 131-141.
42. Kalefetoğlu Macar, T., Ekmekçi, Y., 2009. Alterations in Photochemical and Physiological Activities of Chickpea (Cicer arietinumL.) Cultivars under Drought Stress. Journal of Agronomy and Crop Science 195, 335-346.
43. Karatas, I., Ozturk, L., Demir, Y., Unlukara, A., Kurunc, A., Duzdemir, O., 2014. Alterations in antioxidant enzyme activities and proline content in pea leaves under long-term drought stress. Toxicology and industrial health 30, 693-700.
44. Kebbas, S., Benseddik, T., Makhlouf, H., Aid, F., 2018. Physiological and Biochemical Behaviour of Gleditsia triacanthos L. Young Seedlings Under Drought Stress Conditions. Notulae Botanicae Horti Agrobotanici Cluj-Napoca 46, 585-592.
45. Khaleghi, A., Naderi, R., Brunetti, C., Maserti, B.E., Salami, S.A., Babalar, M., 2019. Morphological, physiochemical and antioxidant responses of Maclura pomifera to drought stress. Scientific reports 9, 19250.
46. Kiran, S., 2018. Effects of Vermicompost on Some Morphological, Physiological and Biochemical Parameters of Lettuce (Lactuca sativa var. crispa) under Drought Stress. Notulae Botanicae Horti Agrobotanici Cluj-Napoca 47, 352-358.
47. Klunklin, W., Savage, G., 2017. Effect on Quality Characteristics of Tomatoes Grown Under Well-Watered and Drought Stress Conditions. Foods 6.
48. Li, J., Liu, L., Zhou, H., Li, M., 2018. Improved Viability of Areca (Areca catechu L.) Seedlings under Drought Stress Using a Superabsorbent Polymer. HortScience 53, 1872-1876.
49. Li, K.R., Feng, C.H., 2010. Effects of brassinolide on drought resistance of Xanthoceras sorbifolia seedlings under water stress. Acta Physiologiae Plantarum 33, 1293-1300.
50. Li, X., Zhang, L., Ma, L., 2011. Effects of Preconditioning on Photosynthesis of Rice Seedlings under Water Stress. Procedia Environmental Sciences 11, 1339-1345.
51. Liang, D., Ni, Z., Xia, H., Xie, Y., Lv, X., Wang, J., Lin, L., Deng, Q., Luo, X., 2019. Exogenous melatonin promotes biomass accumulation and photosynthesis of kiwifruit seedlings under drought stress. Scientia Horticulturae 246, 34-43.
52. Lin, J., Zhang, R., Hu, Y., Song, Y., Hänninen, H., Wu, J., 2019. Interactive effects of drought and shading on Torreya grandis seedlings: physiological and growth responses. Trees 33, 951-961.
53. Maghsoudi, K., Emam, Y., Ashraf, M., Arvin, M.J., 2019. Alleviation of field water stress in wheat cultivars by using silicon and salicylic acid applied separately or in combination. Crop and Pasture Science 70, 36-43.
54. Masoumi, H., Masoumi, M., Darvish, F., Daneshian, J., Nourmohammadi, G., Habibi, D., 2010. Change in several Antioxidant Enzymes Activity and Seed Yield by Water Deficit Stress in Soybean (Glycine max L.) Cultivars. Notulae Botanicae Horti Agrobotanici Cluj-Napoca 38, 86-94.
55. Mathur, S., Tomar, R.S., Jajoo, A., 2019. Arbuscular Mycorrhizal fungi (AMF) protects photosynthetic apparatus of wheat under drought stress. Photosynthesis research 139, 227-238.
56. Moles, T.M., Mariotti, L., De Pedro, L.F., Guglielminetti, L., Picciarelli, P., Scartazza, A., 2018. Drought induced changes of leaf-to-root relationships in two tomato genotypes. Plant physiology and biochemistry: PPB 128, 24-31.
57. Nankishore, A., Farrell, A.D., 2016. The response of contrasting tomato genotypes to combined heat and drought stress. J Plant Physiol 202, 75-82.
58. Nazarli, H., Faraji, F., 2011. Response of proline, soluble sugars and antioxidant enzymes in wheat (Triticum aestivum L.) to different irrigation regimes in greenhouse condition. Cercetari agronomice in Moldova 44, 27-33.
59. Nazarli, H., Zardashti, M.R., Darvishzadeh, R., Mohammadi, M., 2011. Change in activity of antioxidative enzymes in young leaves of sunflower (Helianthus annuus L.) by application of super absorbent synthetic polymers under drought stress condition. Australian Journal of Crop Science 5, 1334-1338.
60. Nikolaeva, M.K., Maevskaya, S.N., Shugaev, A.G., Bukhov, N.G., 2010. Effect of drought on chlorophyll content and antioxidant enzyme activities in leaves of three wheat cultivars varying in productivity. Russian Journal of Plant Physiology 57, 87-95.
61. Nouraei, S., Rahimmalek, M., Saeidi, G., 2018. Variation in polyphenolic composition, antioxidants and physiological characteristics of globe artichoke (Cynara cardunculus var. scolymus Hayek L.) as affected by drought stress. Scientia Horticulturae 233, 378-385.
62. Ors, S., Ekinci, M., Yildirim, E., Sahin, U., 2016. Changes in gas exchange capacity and selected physiological properties of squash seedlings (Cucurbita pepoL.) under well-watered and drought stress conditions. Archives of Agronomy and Soil Science 62, 1700-1710.
63. Parveen, A., Liu, W., Hussain, S., Asghar, J., Perveen, S., Xiong, Y., 2019. Silicon Priming Regulates Morpho-Physiological Growth and Oxidative Metabolism in Maize under Drought Stress. Plants 8.
64. Petridis, A., Therios, I., Samouris, G., Koundouras, S., Giannakoula, A., 2012. Effect of water deficit on leaf phenolic composition, gas exchange, oxidative damage and antioxidant activity of four Greek olive (Olea europaea L.) cultivars. Plant physiology and biochemistry: PPB 60, 1-11.
65. Plazas, M., Nguyen, H.T., Gonzalez-Orenga, S., Fita, A., Vicente, O., Prohens, J., Boscaiu, M., 2019. Comparative analysis of the responses to water stress in eggplant (Solanum melongena) cultivars. Plant physiology and biochemistry: PPB 143, 72-82.
66. Pompelli, M.F., Barata-Luís, R., Vitorino, H.S., Gonçalves, E.R., Rolim, E.V., Santos, M.G., Almeida-Cortez, J.S., Ferreira, V.M., Lemos, E.E., Endres, L., 2010. Photosynthesis, photoprotection and antioxidant activity of purging nut under drought deficit and recovery. Biomass and Bioenergy 34, 1207-1215.
67. Pourghayoumi, M., Rahemi, M., Bakhshi, D., Aalami, A., Kamgar-Haghighi, A.A., 2017. Responses of pomegranate cultivars to severe water stress and recovery: changes on antioxidant enzyme activities, gene expression patterns and water stress responsive metabolites. Physiology and molecular biology of plants: an international journal of functional plant biology 23, 321-330.
68. Rigui, A.P., Carvalho, V., Wendt Dos Santos, A.L., Morvan-Bertrand, A., Prud'homme, M.P., Machado de Carvalho, M.A., Gaspar, M., 2019. Fructan and antioxidant metabolisms in plants of Lolium perenne under drought are modulated by exogenous nitric oxide. Plant physiology and biochemistry: PPB 145, 205-215.
69. Rolando, J.L., Ramírez, D.A., Yactayo, W., Monneveux, P., Quiroz, R., 2015. Leaf greenness as a drought tolerance related trait in potato (Solanum tuberosum L.). Environmental and Experimental Botany 110, 27-35.
70. Samota, M.K., Sasi, M., Singh, A., 2017. Impact of Seed Priming on Proline Content and Antioxidant Enzymes to Mitigate Drought Stress in Rice Genotype. International Journal of Current Microbiology and Applied Sciences 6, 2459-2466.
71. Sanchez-Rodriguez, E., Rubio-Wilhelmi Mdel, M., Blasco, B., Leyva, R., Romero, L., Ruiz, J.M., 2012. Antioxidant response resides in the shoot in reciprocal grafts of drought-tolerant and drought-sensitive cultivars in tomato under water stress. Plant science: an international journal of experimental plant biology 188-189, 89-96.
72. Sedaghat, M., Tahmasebi-Sarvestani, Z., Emam, Y., Mokhtassi-Bidgoli, A., 2017. Physiological and antioxidant responses of winter wheat cultivars to strigolactone and salicylic acid in drought. Plant physiology and biochemistry: PPB 119, 59-69.
73. Sharifi, P., Mohammadkhani, N., 2016. Effects of Drought Stress on Photosynthesis Factors in Wheat Genotypes during Anthesis. Cereal Research Communications 44, 229-239.
74. Shawon, R.A., Kang, B.S., Lee, S.G., Kim, S.K., Ju Lee, H., Katrich, E., Gorinstein, S., Ku, Y.G., 2020. Influence of drought stress on bioactive compounds, antioxidant enzymes and glucosinolate contents of Chinese cabbage (Brassica rapa). Food chemistry 308, 125657.
75. Sheoran, S., Thakur, V., Narwal, S., Turan, R., Mamrutha, H.M., Singh, V., Tiwari, V., Sharma, I., 2015. Differential Activity and Expression Profile of Antioxidant Enzymes and Physiological Changes in Wheat (Triticum aestivum L.) Under Drought. Applied biochemistry and biotechnology 177, 1282-1298.
76. Siddiqui, Z.S., Shahid, H., Cho, J.-I., Park, S.-H., Ryu, T.-H., Park, S.-C., 2016. Physiological responses of two halophytic grass species under drought stress environment. Acta Botanica Croatica 75, 31-38.
77. Silva, E.N., Ferreira-Silva, S.L., Fontenele, A.d.V., Ribeiro, R.V., Viégas, R.A., Silveira, J.A.G., 2010. Photosynthetic changes and protective mechanisms against oxidative damage subjected to isolated and combined drought and heat stresses in Jatropha curcas plants. Journal of Plant Physiology 167, 1157-1164.
78. Sperdouli, I., Moustakas, M., 2012. Interaction of proline, sugars, and anthocyanins during photosynthetic acclimation of Arabidopsis thaliana to drought stress. Journal of Plant Physiology 169, 577-585.
79. Sun, C., Li, X., Hu, Y., Zhao, P., Xu, T., Sun, J., Gao, X., 2015. Proline, Sugars, and Antioxidant Enzymes Respond to Drought Stress in the Leaves of Strawberry Plants. Korean Journal of Horticultural Science and Technology 33, 625-632.
80. Vaseva, I., Akiscan, Y., Simova-Stoilova, L., Kostadinova, A., Nenkova, R., Anders, I., Feller, U., Demirevska, K., 2012. Antioxidant response to drought in red and white clover. Acta Physiologiae Plantarum 34, 1689-1699.
81. Yactayo, W., Ramírez, D.A., Gutiérrez, R., Mares, V., Posadas, A., Quiroz, R., 2013. Effect of partial root-zone drying irrigation timing on potato tuber yield and water use efficiency. Agricultural Water Management 123, 65-70.
82. Yang, F., Hu, J., Li, J., Wu, X., Qian, Y., 2009. Chitosan enhances leaf membrane stability and antioxidant enzyme activities in apple seedlings under drought stress. Plant Growth Regulation 58, 131-136.
83. Zhang, W.-E., Wang, F., Pan, X.-J., Tian, Z.-G., Zhao, X.-M., 2013. Antioxidant Enzymes and Photosynthetic Responses to Drought Stress of Three Canna edulis Cultivars. Korean Journal of Horticultural Science and Technology 31, 677-686.
84. Zhang, X., Lei, L., Lai, J., Zhao, H., Song, W., 2018. Effects of drought stress and water recovery on physiological responses and gene expression in maize seedlings. BMC plant biology 18.

**Table S2.** Raw data used for our meta-analysis.

| **id** | **reference** | **latitude** | **longitude** | **species** | **ED** | **DI** | **PI** | **performance** | **PT** | **yi** | **vi** | **wi** |
| --- | --- | --- | --- | --- | --- | --- | --- | --- | --- | --- | --- | --- |
| 1 | Abedi et al., 2010 | 29.4 | 52.8 | Brassica napus L. | 21 | 0.4 | SOD | EA | L | 0.094 | 0.931 | 1.074 |
| 2 | Abedi et al., 2010 | 29.4 | 52.8 | Brassica napus L. | 21 | 0.7 | SOD | EA | L | 0.746 | 1.283 | 0.779 |
| 3 | Abedi et al., 2010 | 29.4 | 52.8 | Brassica napus L. | 21 | 0.4 | CAT | EA | L | -0.955 | 20.455 | 0.049 |
| 4 | Abedi et al., 2010 | 29.4 | 52.8 | Brassica napus L. | 21 | 0.7 | CAT | EA | L | -4.980 | 3600.366 | 0.000 |
| 5 | Abedi et al., 2010 | 29.4 | 52.8 | Brassica napus L. | 21 | 0.4 | POD | EA | L | 0.062 | 1.124 | 0.889 |
| 6 | Abedi et al., 2010 | 29.4 | 52.8 | Brassica napus L. | 21 | 0.7 | POD | EA | L | 0.910 | 0.861 | 1.161 |
| 7 | Abid et al., 2016 | 36.7 | 10.23 | Vicia faba L. | 28 | 0.33 | Fv/Fm | PS | L | -0.013 | 0.004 | 241.091 |
| 8 | Abid et al., 2016 | 36.7 | 10.23 | Vicia faba L. | 28 | 0.67 | Fv/Fm | PS | L | -0.026 | 0.003 | 356.763 |
| 9 | Abid et al., 2016 | 36.7 | 10.23 | Vicia faba L. | 28 | 0.33 | Fv/Fm | PS | L | -0.039 | 0.003 | 333.306 |
| 10 | Abid et al., 2016 | 36.7 | 10.23 | Vicia faba L. | 28 | 0.67 | Fv/Fm | PS | L | -0.180 | 0.004 | 285.047 |
| 11 | Abid et al., 2016 | 36.7 | 10.23 | Vicia faba L. | 28 | 0.33 | qP | PS | L | 0.000 | 0.000 | 5160.976 |
| 12 | Abid et al., 2016 | 36.7 | 10.23 | Vicia faba L. | 28 | 0.67 | qP | PS | L | -0.011 | 0.000 | 3424.194 |
| 13 | Abid et al., 2016 | 36.7 | 10.23 | Vicia faba L. | 28 | 0.33 | qP | PS | L | -0.011 | 0.000 | 6508.451 |
| 14 | Abid et al., 2016 | 36.7 | 10.23 | Vicia faba L. | 28 | 0.67 | qP | PS | L | -0.045 | 0.330 | 3.027 |
| 15 | Abid et al., 2016 | 36.7 | 10.23 | Vicia faba L. | 28 | 0.33 | Proline | NEA | L | 0.652 | 0.124 | 8.086 |
| 16 | Abid et al., 2016 | 36.7 | 10.23 | Vicia faba L. | 28 | 0.67 | Proline | NEA | L | 1.112 | 0.103 | 9.700 |
| 17 | Abid et al., 2016 | 36.7 | 10.23 | Vicia faba L. | 28 | 0.33 | Proline | NEA | L | 0.194 | 0.070 | 14.228 |
| 18 | Abid et al., 2016 | 36.7 | 10.23 | Vicia faba L. | 28 | 0.67 | Proline | NEA | L | 0.745 | 0.081 | 12.363 |
| 19 | Abid et al., 2016 | 36.7 | 10.23 | Vicia faba L. | 28 | 0.33 | CAT | EA | L | 0.140 | 0.009 | 105.874 |
| 20 | Abid et al., 2016 | 36.7 | 10.23 | Vicia faba L. | 28 | 0.67 | CAT | EA | L | 0.750 | 0.009 | 108.175 |
| 21 | Abid et al., 2016 | 36.7 | 10.23 | Vicia faba L. | 28 | 0.33 | CAT | EA | L | 0.063 | 0.003 | 368.530 |
| 22 | Abid et al., 2016 | 36.7 | 10.23 | Vicia faba L. | 28 | 0.67 | CAT | EA | L | 0.300 | 0.004 | 234.038 |
| 23 | Abid et al., 2016 | 36.7 | 10.23 | Vicia faba L. | 28 | 0.33 | APX | EA | L | 0.112 | 0.014 | 70.728 |
| 24 | Abid et al., 2016 | 36.7 | 10.23 | Vicia faba L. | 28 | 0.67 | APX | EA | L | -0.120 | 0.033 | 30.618 |
| 25 | Abid et al., 2016 | 36.7 | 10.23 | Vicia faba L. | 28 | 0.33 | APX | EA | L | -0.067 | 0.013 | 77.461 |
| 26 | Abid et al., 2016 | 36.7 | 10.23 | Vicia faba L. | 28 | 0.67 | APX | EA | L | -0.115 | 0.018 | 56.913 |
| 27 | Abid et al., 2016 | 36.7 | 10.23 | Vicia faba L. | 28 | 0.33 | SOD | EA | L | 0.819 | 0.041 | 24.377 |
| 28 | Abid et al., 2016 | 36.7 | 10.23 | Vicia faba L. | 28 | 0.67 | SOD | EA | L | 1.504 | 0.059 | 17.003 |
| 29 | Abid et al., 2016 | 36.7 | 10.23 | Vicia faba L. | 28 | 0.33 | SOD | EA | L | 0.563 | 0.067 | 14.831 |
| 30 | Abid et al., 2016 | 36.7 | 10.23 | Vicia faba L. | 28 | 0.67 | SOD | EA | L | 1.223 | 0.058 | 17.214 |
| 31 | Abid et al., 2016 | 36.7 | 10.23 | Vicia faba L. | 28 | 0.33 | POD | EA | L | 0.585 | 0.027 | 36.800 |
| 32 | Abid et al., 2016 | 36.7 | 10.23 | Vicia faba L. | 28 | 0.67 | POD | EA | L | 0.954 | 0.035 | 28.666 |
| 33 | Abid et al., 2016 | 36.7 | 10.23 | Vicia faba L. | 28 | 0.33 | POD | EA | L | 0.409 | 0.038 | 26.346 |
| 34 | Abid et al., 2016 | 36.7 | 10.23 | Vicia faba L. | 28 | 0.67 | POD | EA | L | -0.494 | 0.027 | 36.733 |
| 35 | Abuelsoud et al., 2019 | 53.4 | 9.7 | Euphorbia tirucalli L. | 180 | 0.6 | ROS | PMP | S | 0.108 | 0.000 | 22384.873 |
| 36 | Abuelsoud et al., 2019 | 53.4 | 9.7 | Euphorbia tirucalli L. | 180 | 0.6 | ROS | PMP | L | 0.184 | 0.000 | 60393.930 |
| 37 | Abuelsoud et al., 2019 | 53.4 | 9.7 | Euphorbia tirucalli L. | 180 | 0.6 | MDA | PMP | S | -0.167 | 0.000 | 5842.929 |
| 38 | Abuelsoud et al., 2019 | 53.4 | 9.7 | Euphorbia tirucalli L. | 180 | 0.6 | MDA | PMP | L | 0.085 | 0.000 | 3915.910 |
| 39 | Abuelsoud et al., 2019 | 53.4 | 9.7 | Euphorbia tirucalli L. | 180 | 0.6 | Proline | NEA | S | 0.907 | 0.017 | 59.646 |
| 40 | Abuelsoud et al., 2019 | 53.4 | 9.7 | Euphorbia tirucalli L. | 180 | 0.6 | Proline | NEA | L | 0.238 | 0.004 | 260.674 |
| 41 | Abuelsoud et al., 2019 | 53.4 | 9.7 | Euphorbia tirucalli L. | 180 | 0.6 | SOD | EA | S | -0.216 | 0.005 | 184.393 |
| 42 | Abuelsoud et al., 2019 | 53.4 | 9.7 | Euphorbia tirucalli L. | 180 | 0.6 | SOD | EA | L | -0.280 | 0.004 | 244.323 |
| 43 | Abuelsoud et al., 2019 | 53.4 | 9.7 | Euphorbia tirucalli L. | 180 | 0.6 | CAT | EA | S | 0.184 | 0.018 | 55.989 |
| 44 | Abuelsoud et al., 2019 | 53.4 | 9.7 | Euphorbia tirucalli L. | 180 | 0.6 | CAT | EA | L | -0.309 | 0.004 | 280.427 |
| 45 | Abuelsoud et al., 2019 | 53.4 | 9.7 | Euphorbia tirucalli L. | 180 | 0.6 | POD | EA | S | 1.296 | 0.077 | 12.907 |
| 46 | Abuelsoud et al., 2019 | 53.4 | 9.7 | Euphorbia tirucalli L. | 180 | 0.6 | POD | EA | L | -0.538 | 0.013 | 79.490 |
| 47 | Abuelsoud et al., 2019 | 53.4 | 9.7 | Euphorbia tirucalli L. | 180 | 0.6 | APX | EA | S | 0.374 | 0.002 | 500.757 |
| 48 | Abuelsoud et al., 2019 | 53.4 | 9.7 | Euphorbia tirucalli L. | 180 | 0.6 | APX | EA | L | -0.555 | 0.016 | 62.320 |
| 49 | Abuelsoud et al., 2019 | 53.4 | 9.7 | Euphorbia tirucalli L. | 180 | 0.6 | GR | EA | S | 0.187 | 0.004 | 225.462 |
| 50 | Abuelsoud et al., 2019 | 53.4 | 9.7 | Euphorbia tirucalli L. | 180 | 0.6 | GR | EA | L | -0.326 | 0.015 | 65.533 |
| 51 | Agami et al., 2017 | 29.6 | 30.9 | Triticum aestivum L. | 130 | 0.4 | Dry weight | Growth | S | -0.317 | 0.000 | 4532.295 |
| 52 | Agami et al., 2017 | 29.6 | 30.9 | Triticum aestivum L. | 130 | 0.4 | Dry weight | Growth | R | -1.037 | 0.001 | 827.940 |
| 53 | Agami et al., 2017 | 29.6 | 30.9 | Triticum aestivum L. | 130 | 0.4 | Chl | PS | L | -0.618 | 0.001 | 1985.545 |
| 54 | Agami et al., 2017 | 29.6 | 30.9 | Triticum aestivum L. | 130 | 0.4 | Car | NEA | L | -0.256 | 0.002 | 540.211 |
| 55 | Agami et al., 2017 | 29.6 | 30.9 | Triticum aestivum L. | 130 | 0.4 | Proline | NEA | L | 0.277 | 0.001 | 992.634 |
| 56 | Agami et al., 2017 | 29.6 | 30.9 | Triticum aestivum L. | 130 | 0.4 | Protein | Growth | L | 0.679 | 0.000 | 9615.343 |
| 57 | Agami et al., 2017 | 29.6 | 30.9 | Triticum aestivum L. | 130 | 0.4 | SOD | EA | L | 0.176 | 0.000 | 4139.247 |
| 58 | Agami et al., 2017 | 29.6 | 30.9 | Triticum aestivum L. | 130 | 0.4 | POD | EA | L | 0.416 | 0.001 | 1546.455 |
| 59 | Ashraf et al., 2014 | 31.5 | 73.1 | Zea mays L. | 14 | 0.4 | Dry weight | Growth | S | -0.515 | 0.080 | 12.442 |
| 60 | Ashraf et al., 2014 | 31.5 | 73.1 | Zea mays L. | 14 | 0.4 | Dry weight | Growth | R | -1.081 | 0.062 | 16.121 |
| 61 | Ashraf et al., 2014 | 31.5 | 73.1 | Zea mays L. | 14 | 0.4 | Dry weight | Growth | L | -0.660 | 0.031 | 32.468 |
| 62 | Ashraf et al., 2014 | 31.5 | 73.1 | Zea mays L. | 14 | 0.4 | Chl | PS | L | -0.577 | 0.015 | 68.804 |
| 63 | Ashraf et al., 2014 | 31.5 | 73.1 | Zea mays L. | 14 | 0.4 | Chl | PS | L | -0.369 | 0.015 | 67.174 |
| 64 | Ashraf et al., 2014 | 31.5 | 73.1 | Zea mays L. | 14 | 0.4 | POD | EA | L | 0.480 | 0.018 | 54.431 |
| 65 | Ashraf et al., 2014 | 31.5 | 73.1 | Zea mays L. | 14 | 0.4 | CAT | EA | L | -0.329 | 0.058 | 17.164 |
| 66 | Ashraf et al., 2014 | 31.5 | 73.1 | Zea mays L. | 14 | 0.4 | SOD | EA | L | 0.491 | 0.017 | 58.517 |
| 67 | Ashraf et al., 2014 | 31.5 | 73.1 | Zea mays L. | 14 | 0.4 | ROS | PMP | L | 1.127 | 0.094 | 10.626 |
| 68 | Ashraf et al., 2014 | 31.5 | 73.1 | Zea mays L. | 14 | 0.4 | Protein | Growth | L | 0.875 | 0.013 | 77.217 |
| 69 | Ashraf et al., 2014 | 31.5 | 73.1 | Zea mays L. | 14 | 0.4 | MDA | PMP | L | 1.397 | 0.061 | 16.528 |
| 70 | Bandeppa et al., 2019 | 28.7 | 77.1 | Brassica juncea L | 50 | 0.5 | Dry weight | Growth | S | -0.162 | 0.008 | 120.273 |
| 71 | Bandeppa et al., 2019 | 28.7 | 77.1 | Brassica juncea L | 50 | 0.5 | Dry weight | Growth | R | -0.519 | 0.030 | 33.268 |
| 72 | Bandeppa et al., 2019 | 28.7 | 77.1 | Brassica juncea L | 45 | 0.5 | Proline | NEA | L | 1.147 | 0.006 | 169.703 |
| 73 | Bandeppa et al., 2019 | 28.7 | 77.1 | Brassica juncea L | 45 | 0.5 | CAT | EA | L | 0.167 | 0.006 | 169.703 |
| 74 | Bandeppa et al., 2019 | 28.7 | 77.1 | Brassica juncea L | 45 | 0.5 | POD | EA | L | 0.060 | 0.006 | 169.703 |
| 75 | Bandeppa et al., 2019 | 28.7 | 77.1 | Brassica juncea L | 45 | 0.5 | GR | EA | L | -0.298 | 0.006 | 169.703 |
| 76 | Bandeppa et al., 2019 | 28.7 | 77.1 | Brassica juncea L | 45 | 0.5 | SOD | EA | L | 0.060 | 0.006 | 169.703 |
| 77 | Bandeppa et al., 2019 | 28.7 | 77.1 | Brassica juncea L | 45 | 0.5 | ROS | PMP | L | 0.091 | 0.006 | 169.703 |
| 78 | Bandeppa et al., 2019 | 28.7 | 77.1 | Brassica juncea L | 45 | 0.5 | ABA | NEA | L | -0.297 | 0.006 | 169.703 |
| 79 | Bano et al., 2012 | 33.6 | 73.1 | Triticum aestivum L. | 3 | 0.470588 | SOD | EA | L | 0.223 | 0.004 | 226.271 |
| 80 | Bano et al., 2012 | 33.6 | 73.1 | Triticum aestivum L. | 3 | 0.470588 | POD | EA | L | 0.105 | 0.004 | 226.271 |
| 81 | Bano et al., 2012 | 33.6 | 73.1 | Triticum aestivum L. | 3 | 0.470588 | SOD | EA | L | 0.405 | 0.004 | 226.271 |
| 82 | Bano et al., 2012 | 33.6 | 73.1 | Triticum aestivum L. | 3 | 0.470588 | POD | EA | L | 0.182 | 0.004 | 226.271 |
| 83 | Bano et al., 2012 | 33.6 | 73.1 | Triticum aestivum L. | 3 | 0.470588 | ABA | NEA | L | 0.628 | 0.004 | 226.271 |
| 84 | Bano et al., 2012 | 33.6 | 73.1 | Triticum aestivum L. | 3 | 0.470588 | ABA | NEA | L | 0.640 | 0.004 | 226.271 |
| 85 | Baroowa et al., 2016 | 26.5 | 93.2 | Vigna radiata L. | 15 | 0.35 | MDA | PMP | L | 0.507 | 0.008 | 120.683 |
| 86 | Baroowa et al., 2016 | 26.5 | 93.2 | Vigna radiata L. | 15 | 0.35 | MDA | PMP | L | 0.593 | 0.010 | 104.169 |
| 87 | Baroowa et al., 2016 | 26.5 | 93.2 | Vigna radiata L. | 15 | 0.35 | MDA | PMP | L | 0.249 | 0.018 | 56.575 |
| 88 | Baroowa et al., 2016 | 26.5 | 93.2 | Vigna radiata L. | 15 | 0.35 | MDA | PMP | L | 0.161 | 0.002 | 527.705 |
| 89 | Baroowa et al., 2016 | 26.5 | 93.2 | Vigna radiata L. | 15 | 0.35 | SOD | EA | L | 0.076 | 0.007 | 135.410 |
| 90 | Baroowa et al., 2016 | 26.5 | 93.2 | Vigna radiata L. | 15 | 0.35 | SOD | EA | L | 0.063 | 0.006 | 161.637 |
| 91 | Baroowa et al., 2016 | 26.5 | 93.2 | Vigna radiata L. | 15 | 0.35 | SOD | EA | L | 0.065 | 0.005 | 213.842 |
| 92 | Baroowa et al., 2016 | 26.5 | 93.2 | Vigna radiata L. | 15 | 0.35 | SOD | EA | L | 0.406 | 0.003 | 317.471 |
| 93 | Baroowa et al., 2016 | 26.5 | 93.2 | Vigna radiata L. | 15 | 0.35 | Protein | Growth | L | -0.615 | 0.008 | 127.365 |
| 94 | Baroowa et al., 2016 | 26.5 | 93.2 | Vigna radiata L. | 15 | 0.35 | Protein | Growth | L | -0.678 | 0.013 | 79.081 |
| 95 | Baroowa et al., 2016 | 26.5 | 93.2 | Vigna radiata L. | 15 | 0.35 | Protein | Growth | L | -0.346 | 0.002 | 478.310 |
| 96 | Baroowa et al., 2016 | 26.5 | 93.2 | Vigna radiata L. | 15 | 0.35 | Protein | Growth | L | -0.052 | 0.000 | 4607.895 |
| 97 | Baroowa et al., 2016 | 26.5 | 93.2 | Vigna radiata L. | 15 | 0.35 | CAT | EA | L | 0.629 | 0.017 | 58.632 |
| 98 | Baroowa et al., 2016 | 26.5 | 93.2 | Vigna radiata L. | 15 | 0.35 | CAT | EA | L | 0.626 | 0.047 | 21.237 |
| 99 | Baroowa et al., 2016 | 26.5 | 93.2 | Vigna radiata L. | 15 | 0.35 | CAT | EA | L | 0.718 | 0.004 | 229.474 |
| 100 | Baroowa et al., 2016 | 26.5 | 93.2 | Vigna radiata L. | 15 | 0.35 | CAT | EA | L | 0.762 | 0.050 | 19.921 |
| 101 | Batista et al., 2019 | -9.4 | -47.6 | Glycine max (L.) Merrill | 30 | 0.5 | Dry weight | Growth | P | -0.114 | 0.000 | 5517.151 |
| 102 | Batista et al., 2019 | -9.4 | -47.6 | Glycine max (L.) Merrill | 30 | 0.5 | Dry weight | Growth | L | -0.833 | 0.001 | 910.687 |
| 103 | Batista et al., 2019 | -9.4 | -47.6 | Glycine max (L.) Merrill | 30 | 0.5 | Dry weight | Growth | S | -0.576 | 0.001 | 773.385 |
| 104 | Batista et al., 2019 | -9.4 | -47.6 | Glycine max (L.) Merrill | 30 | 0.5 | Dry weight | Growth | R | -0.630 | 0.003 | 380.504 |
| 105 | Batista et al., 2019 | -9.4 | -47.6 | Glycine max (L.) Merrill | 60 | 0.5 | Dry weight | Growth | P | -0.319 | 0.000 | 2385.542 |
| 106 | Batista et al., 2019 | -9.4 | -47.6 | Glycine max (L.) Merrill | 60 | 0.5 | Dry weight | Growth | L | -0.551 | 0.000 | 5153.968 |
| 107 | Batista et al., 2019 | -9.4 | -47.6 | Glycine max (L.) Merrill | 60 | 0.5 | Dry weight | Growth | S | -0.473 | 0.000 | 8226.417 |
| 108 | Batista et al., 2019 | -9.4 | -47.6 | Glycine max (L.) Merrill | 60 | 0.5 | Dry weight | Growth | R | -0.323 | 0.002 | 457.532 |
| 109 | Batista et al., 2019 | -9.4 | -47.6 | Glycine max (L.) Merrill | 30 | 0.5 | Chl | PS | L | -0.222 | 0.002 | 584.922 |
| 110 | Batista et al., 2019 | -9.4 | -47.6 | Glycine max (L.) Merrill | 30 | 0.5 | Chl | PS | L | -0.396 | 0.009 | 110.488 |
| 111 | Batista et al., 2019 | -9.4 | -47.6 | Glycine max (L.) Merrill | 60 | 0.5 | Chl | PS | L | -0.396 | 0.001 | 1665.934 |
| 112 | Batista et al., 2019 | -9.4 | -47.6 | Glycine max (L.) Merrill | 60 | 0.5 | Chl | PS | L | -0.215 | 0.000 | 2626.588 |
| 113 | Batista et al., 2019 | -9.4 | -47.6 | Glycine max (L.) Merrill | 30 | 0.5 | Fv/Fm | PS | L | -0.089 | 0.000 | 20130.315 |
| 114 | Batista et al., 2019 | -9.4 | -47.6 | Glycine max (L.) Merrill | 60 | 0.5 | Fv/Fm | PS | L | -0.112 | 0.000 | 4903.947 |
| 115 | Batista et al., 2019 | -9.4 | -47.6 | Glycine max (L.) Merrill | 30 | 0.5 | MDA | PMP | L | 0.462 | 0.006 | 176.711 |
| 116 | Batista et al., 2019 | -9.4 | -47.6 | Glycine max (L.) Merrill | 30 | 0.5 | SOD | EA | L | 0.460 | 0.001 | 1270.110 |
| 117 | Batista et al., 2019 | -9.4 | -47.6 | Glycine max (L.) Merrill | 30 | 0.5 | CAT | EA | L | 0.505 | 0.005 | 219.315 |
| 118 | Batista et al., 2019 | -9.4 | -47.6 | Glycine max (L.) Merrill | 30 | 0.5 | APX | EA | L | 0.391 | 0.006 | 157.656 |
| 119 | Batista et al., 2019 | -9.4 | -47.6 | Glycine max (L.) Merrill | 30 | 0.5 | POD | EA | L | -0.239 | 0.000 | 3050.308 |
| 120 | Batista et al., 2019 | -9.4 | -47.6 | Glycine max (L.) Merrill | 60 | 0.5 | MDA | PMP | L | 0.586 | 0.003 | 329.574 |
| 121 | Batista et al., 2019 | -9.4 | -47.6 | Glycine max (L.) Merrill | 60 | 0.5 | SOD | EA | L | 0.343 | 0.005 | 194.547 |
| 122 | Batista et al., 2019 | -9.4 | -47.6 | Glycine max (L.) Merrill | 60 | 0.5 | CAT | EA | L | 0.288 | 0.000 | 2094.785 |
| 123 | Batista et al., 2019 | -9.4 | -47.6 | Glycine max (L.) Merrill | 60 | 0.5 | APX | EA | L | 0.310 | 0.000 | 2090.834 |
| 124 | Batista et al., 2019 | -9.4 | -47.6 | Glycine max (L.) Merrill | 60 | 0.5 | POD | EA | L | 0.405 | 0.004 | 257.920 |
| 125 | Bayat et al., 2019 | 31.8 | 59.3 | Salvia nemorosa L. | 10 | 0.5 | Dry weight | Growth | L | -0.223 | 0.001 | 942.768 |
| 126 | Bayat et al., 2019 | 31.8 | 59.3 | Salvia nemorosa L. | 10 | 0.5 | Dry weight | Growth | R | -0.493 | 0.002 | 575.031 |
| 127 | Bayat et al., 2019 | 31.8 | 59.3 | Salvia nemorosa L. | 10 | 0.5 | Dry weight | Growth | S | -0.457 | 0.001 | 1010.211 |
| 128 | Bayat et al., 2019 | 31.8 | 59.3 | Salvia nemorosa L. | 10 | 0.5 | Dry weight | Growth | P | -0.473 | 0.000 | 2198.322 |
| 129 | Bayat et al., 2019 | 31.8 | 59.3 | Salvia nemorosa L. | 10 | 0.5 | EL | PMP | L | 1.158 | 0.010 | 97.593 |
| 130 | Bayat et al., 2019 | 31.8 | 59.3 | Salvia nemorosa L. | 10 | 0.5 | Chl | PS | L | -0.127 | 0.029 | 34.525 |
| 131 | Bayat et al., 2019 | 31.8 | 59.3 | Salvia nemorosa L. | 10 | 0.5 | Chl | PS | L | -0.307 | 0.034 | 29.516 |
| 132 | Bayat et al., 2019 | 31.8 | 59.3 | Salvia nemorosa L. | 10 | 0.5 | CAT | EA | L | 0.455 | 0.002 | 510.112 |
| 133 | Bayat et al., 2019 | 31.8 | 59.3 | Salvia nemorosa L. | 10 | 0.5 | Proline | NEA | L | 1.063 | 0.006 | 174.056 |
| 134 | Bayat et al., 2019 | 31.8 | 59.3 | Salvia nemorosa L. | 10 | 0.5 | POD | EA | L | 0.663 | 0.012 | 84.560 |
| 135 | Belkheiri et al., 2013 | 39.9 | 8.6 | Atriplex nummularia Lindl. | 35 | 0.428571 | Dry weight | Growth | L | -0.588 | 0.000 | 5914.542 |
| 136 | Belkheiri et al., 2013 | 39.9 | 8.6 | Atriplex nummularia Lindl. | 70 | 0.428571 | Dry weight | Growth | L | -0.607 | 0.000 | 4295.486 |
| 137 | Belkheiri et al., 2013 | 39.9 | 8.6 | Atriplex nummularia Lindl. | 105 | 0.428571 | Dry weight | Growth | L | -0.182 | 0.000 | 13166.629 |
| 138 | Belkheiri et al., 2013 | 39.9 | 8.6 | Atriplex halimus L. | 35 | 0.428571 | Dry weight | Growth | L | -0.789 | 0.000 | 7000.389 |
| 139 | Belkheiri et al., 2013 | 39.9 | 8.6 | Atriplex halimus L. | 70 | 0.428571 | Dry weight | Growth | L | -1.544 | 0.000 | 2710.274 |
| 140 | Belkheiri et al., 2013 | 39.9 | 8.6 | Atriplex halimus L. | 105 | 0.428571 | Dry weight | Growth | L | -1.058 | 0.000 | 20079.187 |
| 141 | Belkheiri et al., 2013 | 39.9 | 8.6 | Atriplex nummularia Lindl. | 105 | 0.428571 | ABA | NEA | L | 1.055 | 0.003 | 314.001 |
| 142 | Belkheiri et al., 2013 | 39.9 | 8.6 | Atriplex halimus L. | 105 | 0.428571 | ABA | NEA | L | 2.367 | 0.011 | 88.509 |
| 143 | Borjas-Ventura et al., 2019 | -21.3 | -47.8 | Stylosanthes capitata | 46 | 0.6 | MDA | PMP | L | 0.249 | 0.001 | 1641.125 |
| 144 | Borjas-Ventura et al., 2019 | -21.3 | -47.8 | Stylosanthes capitata | 46 | 0.6 | ROS | PMP | L | 0.307 | 0.020 | 49.029 |
| 145 | Borjas-Ventura et al., 2019 | -21.3 | -47.8 | Stylosanthes capitata | 46 | 0.6 | SOD | EA | L | -0.119 | 0.003 | 325.291 |
| 146 | Borjas-Ventura et al., 2019 | -21.3 | -47.8 | Stylosanthes capitata | 46 | 0.6 | CAT | EA | L | 0.194 | 0.002 | 467.167 |
| 147 | Borjas-Ventura et al., 2019 | -21.3 | -47.8 | Stylosanthes capitata | 46 | 0.6 | APX | EA | L | 0.060 | 0.000 | 20393.916 |
| 148 | Borjas-Ventura et al., 2019 | -21.3 | -47.8 | Stylosanthes capitata | 46 | 0.6 | GR | EA | L | 0.046 | 0.003 | 304.574 |
| 149 | Bouchemal et al., 2017 | 34.2 | 5.8 | Triticum durum Desf. | 10 | 0.7 | MDA | PMP | L | 0.649 | 0.024 | 41.299 |
| 150 | Bouchemal et al., 2017 | 34.2 | 5.8 | Triticum durum Desf. | 10 | 0.7 | MDA | PMP | L | 1.537 | 0.117 | 8.550 |
| 151 | Bouchemal et al., 2017 | 34.2 | 5.8 | Triticum durum Desf. | 10 | 0.7 | MDA | PMP | L | 1.139 | 0.104 | 9.598 |
| 152 | Bouchemal et al., 2017 | 34.2 | 5.8 | Triticum durum Desf. | 10 | 0.7 | MDA | PMP | L | 1.308 | 0.037 | 26.987 |
| 153 | Bouchemal et al., 2017 | 34.2 | 5.8 | Triticum durum Desf. | 10 | 0.7 | MDA | PMP | L | 0.452 | 0.114 | 8.744 |
| 154 | Bouchemal et al., 2017 | 34.2 | 5.8 | Triticum durum Desf. | 10 | 0.7 | MDA | PMP | L | 0.300 | 0.042 | 23.622 |
| 155 | Bouchemal et al., 2017 | 34.2 | 5.8 | Triticum durum Desf. | 10 | 0.7 | MDA | PMP | L | 1.044 | 0.005 | 215.941 |
| 156 | Bouchemal et al., 2017 | 34.2 | 5.8 | Triticum durum Desf. | 10 | 0.7 | MDA | PMP | L | 0.738 | 0.070 | 14.204 |
| 157 | Bouchemal et al., 2017 | 34.2 | 5.8 | Triticum durum Desf. | 10 | 0.7 | MDA | PMP | L | 1.158 | 0.014 | 69.886 |
| 158 | Bouchemal et al., 2017 | 34.2 | 5.8 | Triticum durum Desf. | 10 | 0.7 | MDA | PMP | L | 0.388 | 0.012 | 82.493 |
| 159 | Bouchemal et al., 2017 | 34.2 | 5.8 | Triticum durum Desf. | 10 | 0.7 | ROS | PMP | L | 0.393 | 0.015 | 64.912 |
| 160 | Bouchemal et al., 2017 | 34.2 | 5.8 | Triticum durum Desf. | 10 | 0.7 | ROS | PMP | L | 0.736 | 0.009 | 114.762 |
| 161 | Bouchemal et al., 2017 | 34.2 | 5.8 | Triticum durum Desf. | 10 | 0.7 | ROS | PMP | L | 0.525 | 0.023 | 43.333 |
| 162 | Bouchemal et al., 2017 | 34.2 | 5.8 | Triticum durum Desf. | 10 | 0.7 | ROS | PMP | L | 0.748 | 0.006 | 166.260 |
| 163 | Bouchemal et al., 2017 | 34.2 | 5.8 | Triticum durum Desf. | 10 | 0.7 | ROS | PMP | L | 0.233 | 0.003 | 313.367 |
| 164 | Bouchemal et al., 2017 | 34.2 | 5.8 | Triticum durum Desf. | 10 | 0.7 | ROS | PMP | L | 0.289 | 0.003 | 333.436 |
| 165 | Bouchemal et al., 2017 | 34.2 | 5.8 | Triticum durum Desf. | 10 | 0.7 | ROS | PMP | L | 0.763 | 0.001 | 1548.431 |
| 166 | Bouchemal et al., 2017 | 34.2 | 5.8 | Triticum durum Desf. | 10 | 0.7 | ROS | PMP | L | 0.536 | 0.001 | 868.805 |
| 167 | Bouchemal et al., 2017 | 34.2 | 5.8 | Triticum durum Desf. | 10 | 0.7 | ROS | PMP | L | 0.487 | 0.002 | 422.589 |
| 168 | Bouchemal et al., 2017 | 34.2 | 5.8 | Triticum durum Desf. | 10 | 0.7 | ROS | PMP | L | 0.374 | 0.031 | 31.831 |
| 169 | Bouchemal et al., 2017 | 34.2 | 5.8 | Triticum durum Desf. | 10 | 0.7 | EL | PMP | L | 0.684 | 0.015 | 68.124 |
| 170 | Bouchemal et al., 2017 | 34.2 | 5.8 | Triticum durum Desf. | 10 | 0.7 | EL | PMP | L | 1.059 | 0.010 | 101.121 |
| 171 | Bouchemal et al., 2017 | 34.2 | 5.8 | Triticum durum Desf. | 10 | 0.7 | EL | PMP | L | 0.491 | 0.008 | 123.992 |
| 172 | Bouchemal et al., 2017 | 34.2 | 5.8 | Triticum durum Desf. | 10 | 0.7 | EL | PMP | L | 0.983 | 0.001 | 840.551 |
| 173 | Bouchemal et al., 2017 | 34.2 | 5.8 | Triticum durum Desf. | 10 | 0.7 | EL | PMP | L | 0.723 | 0.011 | 94.661 |
| 174 | Bouchemal et al., 2017 | 34.2 | 5.8 | Triticum durum Desf. | 10 | 0.7 | EL | PMP | L | 0.364 | 0.013 | 79.823 |
| 175 | Bouchemal et al., 2017 | 34.2 | 5.8 | Triticum durum Desf. | 10 | 0.7 | EL | PMP | L | 0.959 | 0.008 | 128.714 |
| 176 | Bouchemal et al., 2017 | 34.2 | 5.8 | Triticum durum Desf. | 10 | 0.7 | EL | PMP | L | 0.584 | 0.010 | 103.410 |
| 177 | Bouchemal et al., 2017 | 34.2 | 5.8 | Triticum durum Desf. | 10 | 0.7 | EL | PMP | L | 0.882 | 0.002 | 407.450 |
| 178 | Bouchemal et al., 2017 | 34.2 | 5.8 | Triticum durum Desf. | 10 | 0.7 | EL | PMP | L | 0.503 | 0.004 | 255.673 |
| 179 | Bouchemal et al., 2017 | 34.2 | 5.8 | Triticum durum Desf. | 10 | 0.7 | CAT | EA | L | 0.868 | 0.046 | 21.894 |
| 180 | Bouchemal et al., 2017 | 34.2 | 5.8 | Triticum durum Desf. | 10 | 0.7 | CAT | EA | L | -0.012 | 0.006 | 173.446 |
| 181 | Bouchemal et al., 2017 | 34.2 | 5.8 | Triticum durum Desf. | 10 | 0.7 | CAT | EA | L | 0.712 | 0.018 | 55.794 |
| 182 | Bouchemal et al., 2017 | 34.2 | 5.8 | Triticum durum Desf. | 10 | 0.7 | CAT | EA | L | 0.081 | 0.006 | 167.502 |
| 183 | Bouchemal et al., 2017 | 34.2 | 5.8 | Triticum durum Desf. | 10 | 0.7 | CAT | EA | L | 1.591 | 0.001 | 686.643 |
| 184 | Bouchemal et al., 2017 | 34.2 | 5.8 | Triticum durum Desf. | 10 | 0.7 | CAT | EA | L | 1.177 | 0.028 | 35.089 |
| 185 | Bouchemal et al., 2017 | 34.2 | 5.8 | Triticum durum Desf. | 10 | 0.7 | CAT | EA | L | 0.285 | 0.045 | 22.261 |
| 186 | Bouchemal et al., 2017 | 34.2 | 5.8 | Triticum durum Desf. | 10 | 0.7 | CAT | EA | L | 1.139 | 0.108 | 9.269 |
| 187 | Bouchemal et al., 2017 | 34.2 | 5.8 | Triticum durum Desf. | 10 | 0.7 | CAT | EA | L | -0.107 | 0.019 | 52.006 |
| 188 | Bouchemal et al., 2017 | 34.2 | 5.8 | Triticum durum Desf. | 10 | 0.7 | CAT | EA | L | 1.593 | 0.022 | 45.841 |
| 189 | Brossa et al., 2015 | 41.4 | 2.1 | Cistus albidus | 50 | 0.5 | Fv/Fm | PS | L | 0.175 | 0.035 | 28.887 |
| 190 | Brossa et al., 2015 | 41.4 | 2.1 | Cistus albidus | 100 | 0.5 | Fv/Fm | PS | L | 0.142 | 0.008 | 129.357 |
| 191 | Brossa et al., 2015 | 41.4 | 2.1 | Cistus albidus | 50 | 0.5 | AsA | NEA | L | 0.517 | 0.005 | 190.601 |
| 192 | Brossa et al., 2015 | 41.4 | 2.1 | Cistus albidus | 100 | 0.5 | AsA | NEA | L | -0.211 | 0.008 | 123.508 |
| 193 | Brossa et al., 2015 | 41.4 | 2.1 | Cistus albidus | 50 | 0.5 | GR | EA | L | 1.784 | 0.022 | 46.149 |
| 194 | Brossa et al., 2015 | 41.4 | 2.1 | Cistus albidus | 100 | 0.5 | GR | EA | L | -1.179 | 0.021 | 47.522 |
| 195 | Campos et al., 2019 | -23.6 | -46.7 | Coffea arabica L. | 21 | 0.6 | Dry weight | Growth | S | -0.433 | 0.000 | 71942.007 |
| 196 | Campos et al., 2019 | -23.6 | -46.7 | Coffea arabica L. | 21 | 0.6 | Dry weight | Growth | R | 0.149 | 0.000 | 60882.153 |
| 197 | Campos et al., 2019 | -23.6 | -46.7 | Coffea arabica L. | 21 | 0.6 | Chl | PS | P | -0.104 | 0.000 | 2134.383 |
| 198 | Campos et al., 2019 | -23.6 | -46.7 | Coffea arabica L. | 21 | 0.6 | SOD | EA | L | 0.376 | 0.001 | 701.503 |
| 199 | Campos et al., 2019 | -23.6 | -46.7 | Coffea arabica L. | 21 | 0.6 | SOD | EA | R | 0.375 | 0.014 | 69.091 |
| 200 | Campos et al., 2019 | -23.6 | -46.7 | Coffea arabica L. | 21 | 0.6 | APX | EA | L | -0.982 | 0.000 | 6145.597 |
| 201 | Campos et al., 2019 | -23.6 | -46.7 | Coffea arabica L. | 21 | 0.6 | APX | EA | R | -0.901 | 0.000 | 5271.808 |
| 202 | Campos et al., 2019 | -23.6 | -46.7 | Coffea arabica L. | 21 | 0.6 | CAT | EA | L | 0.191 | 0.003 | 321.396 |
| 203 | Campos et al., 2019 | -23.6 | -46.7 | Coffea arabica L. | 21 | 0.6 | CAT | EA | R | 0.425 | 0.000 | 2879.273 |
| 204 | Campos et al., 2019 | -23.6 | -46.7 | Coffea arabica L. | 21 | 0.6 | AsA | NEA | L | 0.092 | 0.006 | 169.703 |
| 205 | Campos et al., 2019 | -23.6 | -46.7 | Coffea arabica L. | 21 | 0.6 | AsA | NEA | R | 0.013 | 0.008 | 118.142 |
| 206 | Campos et al., 2019 | -23.6 | -46.7 | Coffea arabica L. | 21 | 0.6 | Proline | NEA | L | 0.272 | 0.002 | 564.119 |
| 207 | Campos et al., 2019 | -23.6 | -46.7 | Coffea arabica L. | 21 | 0.6 | Proline | NEA | R | 0.393 | 0.002 | 422.722 |
| 208 | Chen et al., 2016 | 34.12 | 108.7 | Zea mays L. | 22 | 0.6875 | Fv/Fm | PS | L | -0.188 | 0.000 | 4756.953 |
| 209 | Chen et al., 2016 | 34.12 | 108.7 | Zea mays L. | 22 | 0.6875 | Chl | PS | L | -0.249 | 0.000 | 4318.604 |
| 210 | Chen et al., 2016 | 34.12 | 108.7 | Zea mays L. | 22 | 0.6875 | Car | NEA | L | 0.163 | 0.001 | 1888.451 |
| 211 | Chen et al., 2016 | 34.12 | 108.7 | Zea mays L. | 22 | 0.6875 | ROS | PMP | L | 0.687 | 0.002 | 453.491 |
| 212 | Chen et al., 2016 | 34.12 | 108.7 | Zea mays L. | 22 | 0.6875 | MDA | PMP | L | -0.194 | 0.007 | 151.221 |
| 213 | Chen et al., 2016 | 34.12 | 108.7 | Zea mays L. | 22 | 0.6875 | Dry weight | Growth | S | -0.589 | 0.001 | 985.719 |
| 214 | Cotrozzi et al., 2016 | 43.7 | 10.7 | Quercus ilex | 77 | 0.7 | Proline | NEA | L | 0.595 | 0.003 | 346.321 |
| 215 | Cotrozzi et al., 2016 | 43.7 | 10.7 | Quercus pubescens | 77 | 0.7 | Proline | NEA | L | 1.154 | 0.008 | 128.245 |
| 216 | Cotrozzi et al., 2016 | 43.7 | 10.7 | Quercus cerris | 77 | 0.7 | Proline | NEA | L | 0.440 | 0.022 | 45.321 |
| 217 | Cotrozzi et al., 2016 | 43.7 | 10.7 | Quercus ilex | 77 | 0.7 | MDA | PMP | L | 0.006 | 0.004 | 235.323 |
| 218 | Cotrozzi et al., 2016 | 43.7 | 10.7 | Quercus pubescens | 77 | 0.7 | MDA | PMP | L | 0.067 | 0.000 | 2262.871 |
| 219 | Cotrozzi et al., 2016 | 43.7 | 10.7 | Quercus cerris | 77 | 0.7 | MDA | PMP | L | 0.207 | 0.002 | 466.017 |
| 220 | Cotrozzi et al., 2016 | 43.7 | 10.7 | Quercus ilex | 77 | 0.7 | Fv/Fm | PS | L | -0.024 | 0.000 | 3974.446 |
| 221 | Cotrozzi et al., 2016 | 43.7 | 10.7 | Quercus pubescens | 77 | 0.7 | Fv/Fm | PS | L | 0.000 | 0.000 | 3306.720 |
| 222 | Cotrozzi et al., 2016 | 43.7 | 10.7 | Quercus cerris | 77 | 0.7 | Fv/Fm | PS | L | -0.012 | 0.000 | 2614.313 |
| 223 | DaCosta et al., 2007 | 50.6 | 10.2 | Agrostis capillaris L. | 28 | 0.8 | Fv/Fm | PS | L | 0.818 | 0.004 | 226.271 |
| 224 | DaCosta et al., 2007 | 50.6 | 10.2 | Agrostis stolonifera L. | 28 | 0.8 | Fv/Fm | PS | L | 0.778 | 0.004 | 226.271 |
| 225 | DaCosta et al., 2007 | 50.6 | 10.2 | Agrostis canina L. | 28 | 0.8 | Fv/Fm | PS | L | 0.528 | 0.004 | 226.271 |
| 226 | DaCosta et al., 2007 | 50.6 | 10.2 | Agrostis capillaris L. | 28 | 0.8 | APX | EA | L | 0.216 | 0.004 | 226.271 |
| 227 | DaCosta et al., 2007 | 50.6 | 10.2 | Agrostis stolonifera L. | 28 | 0.8 | APX | EA | L | 0.367 | 0.004 | 226.271 |
| 228 | DaCosta et al., 2007 | 50.6 | 10.2 | Agrostis canina L. | 28 | 0.8 | APX | EA | L | 0.300 | 0.004 | 226.271 |
| 229 | DaCosta et al., 2007 | 50.6 | 10.2 | Agrostis capillaris L. | 28 | 0.8 | SOD | EA | L | 0.508 | 0.004 | 226.271 |
| 230 | DaCosta et al., 2007 | 50.6 | 10.2 | Agrostis stolonifera L. | 28 | 0.8 | SOD | EA | L | 0.608 | 0.004 | 226.271 |
| 231 | DaCosta et al., 2007 | 50.6 | 10.2 | Agrostis canina L. | 28 | 0.8 | SOD | EA | L | 0.406 | 0.004 | 226.271 |
| 232 | DaCosta et al., 2007 | 50.6 | 10.2 | Agrostis capillaris L. | 28 | 0.8 | CAT | EA | L | 0.637 | 0.004 | 226.271 |
| 233 | DaCosta et al., 2007 | 50.6 | 10.2 | Agrostis stolonifera L. | 28 | 0.8 | CAT | EA | L | 0.731 | 0.004 | 226.271 |
| 234 | DaCosta et al., 2007 | 50.6 | 10.2 | Agrostis canina L. | 28 | 0.8 | CAT | EA | L | 0.595 | 0.004 | 226.271 |
| 235 | DaCosta et al., 2007 | 50.6 | 10.2 | Agrostis capillaris L. | 28 | 0.8 | MDA | PMP | L | -1.970 | 0.004 | 226.271 |
| 236 | DaCosta et al., 2007 | 50.6 | 10.2 | Agrostis stolonifera L. | 28 | 0.8 | MDA | PMP | L | -2.072 | 0.004 | 226.271 |
| 237 | DaCosta et al., 2007 | 50.6 | 10.2 | Agrostis canina L. | 28 | 0.8 | MDA | PMP | L | -1.132 | 0.004 | 226.271 |
| 238 | Ditmarova et al., 2009 | 48.9 | 19.5 | Picea abies [L.] Karst | 48 | 0.29 | Proline | NEA | L | 0.516 | 0.003 | 288.189 |
| 239 | Ditmarova et al., 2009 | 48.9 | 19.5 | Picea abies [L.] Karst | 48 | 0.57 | Proline | NEA | L | 1.489 | 0.013 | 78.860 |
| 240 | Ditmarova et al., 2009 | 48.9 | 19.5 | Picea abies [L.] Karst | 48 | 0.29 | Fv/Fm | PS | L | 0.025 | 0.002 | 421.375 |
| 241 | Ditmarova et al., 2009 | 48.9 | 19.5 | Picea abies [L.] Karst | 48 | 0.57 | Fv/Fm | PS | L | -0.380 | 0.008 | 125.851 |
| 242 | Du et al., 2012 | 35.8 | 104.1 | Triticum aestivum L. | 30 | 0.388889 | Dry weight | Growth | R | -0.184 | 0.008 | 130.166 |
| 243 | Du et al., 2012 | 35.8 | 104.1 | Triticum aestivum L. | 30 | 0.61 | Dry weight | Growth | R | -0.327 | 0.006 | 155.413 |
| 244 | Du et al., 2012 | 35.8 | 104.1 | Triticum aestivum L. | 30 | 0.388889 | Dry weight | Growth | R | -0.110 | 0.006 | 157.787 |
| 245 | Du et al., 2012 | 35.8 | 104.1 | Triticum aestivum L. | 30 | 0.61 | Dry weight | Growth | R | -0.265 | 0.006 | 161.039 |
| 246 | Du et al., 2012 | 35.8 | 104.1 | Triticum aestivum L. | 30 | 0.388889 | Dry weight | Growth | L | -0.368 | 0.005 | 198.519 |
| 247 | Du et al., 2012 | 35.8 | 104.1 | Triticum aestivum L. | 30 | 0.61 | Dry weight | Growth | L | -0.832 | 0.006 | 162.409 |
| 248 | Du et al., 2012 | 35.8 | 104.1 | Triticum aestivum L. | 30 | 0.388889 | Dry weight | Growth | L | -0.282 | 0.127 | 7.877 |
| 249 | Du et al., 2012 | 35.8 | 104.1 | Triticum aestivum L. | 30 | 0.61 | Dry weight | Growth | L | -0.786 | 0.127 | 7.869 |
| 250 | Du et al., 2012 | 35.8 | 104.1 | Triticum aestivum L. | 30 | 0.388889 | ABA | NEA | L | 0.255 | 0.006 | 162.409 |
| 251 | Du et al., 2012 | 35.8 | 104.1 | Triticum aestivum L. | 30 | 0.61 | ABA | NEA | L | 1.007 | 0.005 | 203.695 |
| 252 | Du et al., 2012 | 35.8 | 104.1 | Triticum aestivum L. | 30 | 0.388889 | ROS | PMP | L | 0.098 | 0.006 | 168.922 |
| 253 | Du et al., 2012 | 35.8 | 104.1 | Triticum aestivum L. | 30 | 0.61 | ROS | PMP | L | 0.317 | 0.007 | 142.047 |
| 254 | Du et al., 2012 | 35.8 | 104.1 | Triticum aestivum L. | 30 | 0.388889 | ROS | PMP | L | 0.367 | 0.005 | 203.695 |
| 255 | Du et al., 2012 | 35.8 | 104.1 | Triticum aestivum L. | 30 | 0.61 | ROS | PMP | L | 0.749 | 0.005 | 198.519 |
| 256 | Du et al., 2012 | 35.8 | 104.1 | Triticum aestivum L. | 30 | 0.388889 | MDA | PMP | L | 0.048 | 0.009 | 106.738 |
| 257 | Du et al., 2012 | 35.8 | 104.1 | Triticum aestivum L. | 30 | 0.61 | MDA | PMP | L | 0.335 | 0.008 | 123.142 |
| 258 | Du et al., 2012 | 35.8 | 104.1 | Triticum aestivum L. | 30 | 0.388889 | SOD | EA | L | 0.134 | 0.002 | 645.438 |
| 259 | Du et al., 2012 | 35.8 | 104.1 | Triticum aestivum L. | 30 | 0.61 | SOD | EA | L | 0.226 | 0.002 | 580.958 |
| 260 | Du et al., 2012 | 35.8 | 104.1 | Triticum aestivum L. | 30 | 0.388889 | APX | EA | L | 0.040 | 0.004 | 227.848 |
| 261 | Du et al., 2012 | 35.8 | 104.1 | Triticum aestivum L. | 30 | 0.61 | APX | EA | L | 0.329 | 0.005 | 221.391 |
| 262 | Du et al., 2012 | 35.8 | 104.1 | Triticum aestivum L. | 30 | 0.388889 | CAT | EA | L | 0.173 | 0.006 | 160.980 |
| 263 | Du et al., 2012 | 35.8 | 104.1 | Triticum aestivum L. | 30 | 0.61 | CAT | EA | L | 0.395 | 0.005 | 201.452 |
| 264 | Du et al., 2012 | 35.8 | 104.1 | Triticum aestivum L. | 30 | 0.388889 | GR | EA | L | 0.172 | 0.005 | 211.786 |
| 265 | Du et al., 2012 | 35.8 | 104.1 | Triticum aestivum L. | 30 | 0.61 | GR | EA | L | 0.282 | 0.006 | 171.182 |
| 266 | Epron et al., 1997 | 43.6 | 3.9 | Cedrus atlantica | 21 | 0.778947 | Fv/Fm | PS | L | -0.012 | 0.002 | 643.700 |
| 267 | Epron et al., 1997 | 43.6 | 3.9 | Cedrus atlantica | 21 | 0.82 | Fv/Fm | PS | L | -0.025 | 0.000 | 4098.750 |
| 268 | Epron et al., 1997 | 43.6 | 3.9 | Cedrus libam | 21 | 0.78 | Fv/Fm | PS | L | -0.012 | 0.002 | 644.883 |
| 269 | Epron et al., 1997 | 43.6 | 3.9 | Cedrus libam | 21 | 0.821053 | Fv/Fm | PS | L | -0.012 | 0.002 | 644.883 |
| 270 | Fang et al., 2011 | 35.9 | 104.1 | Caragana korshinskii | 12 | 0.88 | Chl | PS | L | 0.582 | 0.004 | 282.838 |
| 271 | Fang et al., 2011 | 35.9 | 104.1 | Caragana korshinskii | 12 | 0.88 | Fv/Fm | PS | L | 0.023 | 0.004 | 282.838 |
| 272 | Fang et al., 2011 | 35.9 | 104.1 | Caragana korshinskii | 20 | 0.88 | Chl | PS | L | 0.750 | 0.004 | 282.838 |
| 273 | Fang et al., 2011 | 35.9 | 104.1 | Caragana korshinskii | 20 | 0.88 | Fv/Fm | PS | L | 0.248 | 0.002 | 412.132 |
| 274 | Gamble et al., 19984 | 31.7 | -98.9 | Triticum aestivum L. | 133 | 0.4 | GR | EA | L | -0.274 | 0.048 | 20.955 |
| 275 | Gamble et al., 19984 | 31.7 | -98.9 | Triticum aestivum L. | 133 | 0.4 | GR | EA | L | 0.049 | 0.004 | 242.622 |
| 276 | Gamble et al., 19984 | 31.7 | -98.9 | Triticum aestivum L. | 133 | 0.4 | GR | EA | L | 0.596 | 0.009 | 108.279 |
| 277 | Gamble et al., 19984 | 31.7 | -98.9 | Triticum aestivum L. | 133 | 0.4 | GR | EA | L | 0.033 | 0.015 | 65.630 |
| 278 | Gamble et al., 19984 | 31.7 | -98.9 | Triticum aestivum L. | 133 | 0.4 | CAT | EA | L | 0.243 | 0.004 | 226.914 |
| 279 | Gamble et al., 19984 | 31.7 | -98.9 | Triticum aestivum L. | 133 | 0.4 | CAT | EA | L | 0.464 | 0.012 | 86.505 |
| 280 | Gamble et al., 19984 | 31.7 | -98.9 | Triticum aestivum L. | 133 | 0.4 | CAT | EA | L | 0.160 | 0.006 | 167.259 |
| 281 | Gamble et al., 19984 | 31.7 | -98.9 | Triticum aestivum L. | 133 | 0.4 | CAT | EA | L | -0.240 | 0.010 | 102.745 |
| 282 | Gamble et al., 19984 | 31.7 | -98.9 | Triticum aestivum L. | 95 | 0.4 | Chl | PS | L | -0.017 | 0.002 | 510.693 |
| 283 | Gamble et al., 19984 | 31.7 | -98.9 | Triticum aestivum L. | 116 | 0.4 | Chl | PS | L | -0.117 | 0.001 | 1576.437 |
| 284 | Gamble et al., 19984 | 31.7 | -98.9 | Triticum aestivum L. | 133 | 0.4 | Chl | PS | L | -0.284 | 0.006 | 174.941 |
| 285 | Gamble et al., 19984 | 31.7 | -98.9 | Triticum aestivum L. | 95 | 0.4 | Protein | Growth | L | -0.071 | 0.001 | 1208.195 |
| 286 | Gamble et al., 19984 | 31.7 | -98.9 | Triticum aestivum L. | 116 | 0.4 | Protein | Growth | L | -0.040 | 0.002 | 406.749 |
| 287 | Gamble et al., 19984 | 31.7 | -98.9 | Triticum aestivum L. | 133 | 0.4 | Protein | Growth | L | -0.019 | 0.005 | 216.613 |
| 288 | Gao et al., 2020 | 41.8 | 123.4 | Adonis amurensis | 5 | 0.5 | Dry weight | Growth | P | -0.059 | 0.000 | 4060.700 |
| 289 | Gao et al., 2020 | 41.8 | 123.4 | Adonis pseudoamurensis | 5 | 0.5 | Dry weight | Growth | P | -0.041 | 0.004 | 262.005 |
| 290 | Gao et al., 2020 | 41.8 | 123.4 | Adonis amurensis | 30 | 0.5 | Dry weight | Growth | P | -0.637 | 0.001 | 821.395 |
| 291 | Gao et al., 2020 | 41.8 | 123.4 | Adonis pseudoamurensis | 30 | 0.5 | Dry weight | Growth | P | -0.702 | 0.003 | 375.241 |
| 292 | Gao et al., 2020 | 41.8 | 123.4 | Adonis amurensis | 5 | 0.5 | ROS | PMP | L | 0.055 | 0.017 | 59.842 |
| 293 | Gao et al., 2020 | 41.8 | 123.4 | Adonis pseudoamurensis | 5 | 0.5 | ROS | PMP | L | -0.017 | 0.004 | 238.401 |
| 294 | Gao et al., 2020 | 41.8 | 123.4 | Adonis amurensis | 30 | 0.5 | ROS | PMP | L | 0.771 | 0.023 | 44.378 |
| 295 | Gao et al., 2020 | 41.8 | 123.4 | Adonis pseudoamurensis | 30 | 0.5 | ROS | PMP | L | 0.326 | 0.002 | 446.003 |
| 296 | Gao et al., 2020 | 41.8 | 123.4 | Adonis amurensis | 5 | 0.5 | MDA | PMP | L | 0.017 | 0.000 | 22562.494 |
| 297 | Gao et al., 2020 | 41.8 | 123.4 | Adonis pseudoamurensis | 5 | 0.5 | MDA | PMP | L | 0.005 | 0.000 | 21113.462 |
| 298 | Gao et al., 2020 | 41.8 | 123.4 | Adonis amurensis | 30 | 0.5 | MDA | PMP | L | 0.291 | 0.001 | 1124.691 |
| 299 | Gao et al., 2020 | 41.8 | 123.4 | Adonis pseudoamurensis | 30 | 0.5 | MDA | PMP | L | 0.439 | 0.001 | 1886.751 |
| 300 | Gao et al., 2020 | 41.8 | 123.4 | Adonis amurensis | 5 | 0.5 | Proline | NEA | L | 0.104 | 0.001 | 1894.225 |
| 301 | Gao et al., 2020 | 41.8 | 123.4 | Adonis pseudoamurensis | 5 | 0.5 | Proline | NEA | L | 0.134 | 0.000 | 2136.321 |
| 302 | Gao et al., 2020 | 41.8 | 123.4 | Adonis amurensis | 30 | 0.5 | Proline | NEA | L | 0.229 | 0.001 | 1875.463 |
| 303 | Gao et al., 2020 | 41.8 | 123.4 | Adonis pseudoamurensis | 30 | 0.5 | Proline | NEA | L | 0.227 | 0.001 | 1926.959 |
| 304 | Gao et al., 2020 | 41.8 | 123.4 | Adonis amurensis | 5 | 0.5 | Protein | Growth | L | 0.152 | 0.000 | 6776.075 |
| 305 | Gao et al., 2020 | 41.8 | 123.4 | Adonis pseudoamurensis | 5 | 0.5 | Protein | Growth | L | 0.049 | 0.001 | 1028.841 |
| 306 | Gao et al., 2020 | 41.8 | 123.4 | Adonis amurensis | 30 | 0.5 | Protein | Growth | L | -0.093 | 0.000 | 5361.022 |
| 307 | Gao et al., 2020 | 41.8 | 123.4 | Adonis pseudoamurensis | 30 | 0.5 | Protein | Growth | L | -0.119 | 0.000 | 13536.843 |
| 308 | Gao et al., 2020 | 41.8 | 123.4 | Adonis amurensis | 5 | 0.5 | CAT | EA | L | -0.307 | 0.001 | 1562.978 |
| 309 | Gao et al., 2020 | 41.8 | 123.4 | Adonis pseudoamurensis | 5 | 0.5 | CAT | EA | L | 0.143 | 0.000 | 6217.540 |
| 310 | Gao et al., 2020 | 41.8 | 123.4 | Adonis amurensis | 30 | 0.5 | CAT | EA | L | 0.258 | 0.000 | 4531.766 |
| 311 | Gao et al., 2020 | 41.8 | 123.4 | Adonis pseudoamurensis | 30 | 0.5 | CAT | EA | L | -0.150 | 0.000 | 2283.796 |
| 312 | Gao et al., 2020 | 41.8 | 123.4 | Adonis amurensis | 5 | 0.5 | POD | EA | L | -0.057 | 0.001 | 902.320 |
| 313 | Gao et al., 2020 | 41.8 | 123.4 | Adonis pseudoamurensis | 5 | 0.5 | POD | EA | L | -0.032 | 0.001 | 779.364 |
| 314 | Gao et al., 2020 | 41.8 | 123.4 | Adonis amurensis | 30 | 0.5 | POD | EA | L | 0.235 | 0.001 | 735.320 |
| 315 | Gao et al., 2020 | 41.8 | 123.4 | Adonis pseudoamurensis | 30 | 0.5 | POD | EA | L | 0.148 | 0.001 | 1382.606 |
| 316 | Gao et al., 2020 | 41.8 | 123.4 | Adonis amurensis | 5 | 0.5 | SOD | EA | L | 0.493 | 0.000 | 2727.955 |
| 317 | Gao et al., 2020 | 41.8 | 123.4 | Adonis pseudoamurensis | 5 | 0.5 | SOD | EA | L | 0.377 | 0.000 | 12804.884 |
| 318 | Gao et al., 2020 | 41.8 | 123.4 | Adonis amurensis | 30 | 0.5 | SOD | EA | L | -0.433 | 0.001 | 1547.586 |
| 319 | Gao et al., 2020 | 41.8 | 123.4 | Adonis pseudoamurensis | 30 | 0.5 | SOD | EA | L | -0.002 | 0.000 | 2428.150 |
| 320 | Gao et al., 2020 | 41.8 | 123.4 | Adonis amurensis | 5 | 0.5 | APX | EA | L | 0.195 | 0.002 | 598.475 |
| 321 | Gao et al., 2020 | 41.8 | 123.4 | Adonis pseudoamurensis | 5 | 0.5 | APX | EA | L | -0.061 | 0.004 | 256.339 |
| 322 | Gao et al., 2020 | 41.8 | 123.4 | Adonis amurensis | 30 | 0.5 | APX | EA | L | 0.860 | 0.001 | 1374.693 |
| 323 | Gao et al., 2020 | 41.8 | 123.4 | Adonis pseudoamurensis | 30 | 0.5 | APX | EA | L | 1.061 | 0.001 | 769.364 |
| 324 | Gao et al., 2020 | 41.8 | 123.4 | Adonis amurensis | 5 | 0.5 | ABA | NEA | L | 0.257 | 0.000 | 4106.145 |
| 325 | Gao et al., 2020 | 41.8 | 123.4 | Adonis pseudoamurensis | 5 | 0.5 | ABA | NEA | L | 0.270 | 0.000 | 33316.635 |
| 326 | Gao et al., 2020 | 41.8 | 123.4 | Adonis amurensis | 30 | 0.5 | ABA | NEA | L | 0.326 | 0.000 | 9883.804 |
| 327 | Gao et al., 2020 | 41.8 | 123.4 | Adonis pseudoamurensis | 30 | 0.5 | ABA | NEA | L | 0.054 | 0.000 | 4085.360 |
| 328 | Ghafar et al., 2019 | 31.5 | 73.2 | Trifolium repens L. | 28 | 0.2 | Dry weight | Growth | S | -1.070 | 0.161 | 6.193 |
| 329 | Ghafar et al., 2019 | 31.5 | 73.2 | Trifolium repens L. | 28 | 0.4 | Dry weight | Growth | S | -1.070 | 0.097 | 10.345 |
| 330 | Ghafar et al., 2019 | 31.5 | 73.2 | Trifolium repens L. | 28 | 0.2 | Dry weight | Growth | R | -0.773 | 0.031 | 32.052 |
| 331 | Ghafar et al., 2019 | 31.5 | 73.2 | Trifolium repens L. | 28 | 0.4 | Dry weight | Growth | R | -0.644 | 0.024 | 41.486 |
| 332 | Ghafar et al., 2019 | 31.5 | 73.2 | Trifolium repens L. | 28 | 0.2 | Chl | PS | L | -0.181 | 0.003 | 311.829 |
| 333 | Ghafar et al., 2019 | 31.5 | 73.2 | Trifolium repens L. | 28 | 0.4 | Chl | PS | L | -0.171 | 0.004 | 269.488 |
| 334 | Ghafar et al., 2019 | 31.5 | 73.2 | Trifolium repens L. | 28 | 0.2 | Chl | PS | L | -0.326 | 0.008 | 117.801 |
| 335 | Ghafar et al., 2019 | 31.5 | 73.2 | Trifolium repens L. | 28 | 0.4 | Chl | PS | L | -0.457 | 0.013 | 78.599 |
| 336 | Ghafar et al., 2019 | 31.5 | 73.2 | Trifolium repens L. | 28 | 0.2 | ABA | NEA | P | 0.274 | 0.003 | 300.560 |
| 337 | Ghafar et al., 2019 | 31.5 | 73.2 | Trifolium repens L. | 28 | 0.4 | ABA | NEA | P | 0.172 | 0.004 | 251.306 |
| 338 | Ghafar et al., 2019 | 31.5 | 73.2 | Trifolium repens L. | 28 | 0.2 | MDA | PMP | P | -0.068 | 0.015 | 65.481 |
| 339 | Ghafar et al., 2019 | 31.5 | 73.2 | Trifolium repens L. | 28 | 0.4 | MDA | PMP | P | -0.589 | 0.033 | 30.100 |
| 340 | Ghafar et al., 2019 | 31.5 | 73.2 | Trifolium repens L. | 28 | 0.2 | Protein | Growth | P | 0.003 | 0.006 | 170.283 |
| 341 | Ghafar et al., 2019 | 31.5 | 73.2 | Trifolium repens L. | 28 | 0.4 | Protein | Growth | P | -0.197 | 0.009 | 116.791 |
| 342 | Ghafar et al., 2019 | 31.5 | 73.2 | Trifolium repens L. | 28 | 0.2 | CAT | EA | L | 0.410 | 0.009 | 115.196 |
| 343 | Ghafar et al., 2019 | 31.5 | 73.2 | Trifolium repens L. | 28 | 0.4 | CAT | EA | L | 0.417 | 0.008 | 118.734 |
| 344 | Ghafar et al., 2019 | 31.5 | 73.2 | Trifolium repens L. | 28 | 0.2 | POD | EA | L | 0.155 | 0.006 | 170.422 |
| 345 | Ghafar et al., 2019 | 31.5 | 73.2 | Trifolium repens L. | 28 | 0.4 | POD | EA | L | 0.494 | 0.005 | 212.686 |
| 346 | Ghafar et al., 2019 | 31.5 | 73.2 | Trifolium repens L. | 28 | 0.2 | SOD | EA | L | 0.400 | 0.005 | 193.587 |
| 347 | Ghafar et al., 2019 | 31.5 | 73.2 | Trifolium repens L. | 28 | 0.4 | SOD | EA | L | 0.354 | 0.006 | 177.071 |
| 348 | Ghanbary et al., 2018 | 33.6 | 46.4 | Quercus brantii Lindl. | 365 | 0.8 | Chl | PS | L | -0.599 | 0.005 | 219.042 |
| 349 | Ghanbary et al., 2018 | 33.6 | 46.4 | Quercus brantii Lindl. | 365 | 0.8 | Car | PS | L | -0.794 | 0.004 | 271.578 |
| 350 | Ghanbary et al., 2018 | 33.6 | 46.4 | Quercus brantii Lindl. | 365 | 0.8 | Proline | NEA | L | 0.777 | 0.003 | 298.569 |
| 351 | Ghanbary et al., 2018 | 33.6 | 46.4 | Quercus brantii Lindl. | 365 | 0.8 | Protein | Growth | L | 0.478 | 0.005 | 198.197 |
| 352 | Ghanbary et al., 2018 | 33.6 | 46.4 | Quercus brantii Lindl. | 365 | 0.8 | MDA | PMP | L | 0.764 | 0.002 | 538.571 |
| 353 | Ghanbary et al., 2018 | 33.6 | 46.4 | Quercus brantii Lindl. | 365 | 0.8 | ROS | PMP | L | 0.435 | 0.025 | 40.779 |
| 354 | Ghanbary et al., 2018 | 33.6 | 46.4 | Quercus brantii Lindl. | 365 | 0.8 | ROS | PMP | L | 0.379 | 0.014 | 73.569 |
| 355 | Ghanbary et al., 2018 | 33.6 | 46.4 | Quercus brantii Lindl. | 365 | 0.8 | POD | EA | L | 1.246 | 0.023 | 43.993 |
| 356 | Ghanbary et al., 2018 | 33.6 | 46.4 | Quercus brantii Lindl. | 365 | 0.8 | SOD | EA | L | 1.371 | 0.013 | 76.307 |
| 357 | Ghanbary et al., 2018 | 33.6 | 46.4 | Quercus brantii Lindl. | 365 | 0.8 | CAT | EA | L | 0.247 | 0.011 | 90.131 |
| 358 | Ghanbary et al., 2018 | 33.6 | 46.4 | Quercus brantii Lindl. | 365 | 0.8 | APX | EA | L | -0.083 | 0.003 | 331.327 |
| 359 | Ghanbary et al., 2018 | 33.6 | 46.4 | Quercus brantii Lindl. | 365 | 0.8 | GR | EA | L | 0.123 | 0.003 | 384.245 |
| 360 | Ghanbary et al., 2018 | 33.6 | 46.4 | Quercus brantii Lindl. | 365 | 0.8 | AsA | NEA | L | 1.148 | 0.003 | 323.261 |
| 361 | Gholami et al., 2012 | 29.7 | 52.8 | Ficus carica L. | 14 | 0.7 | Chl | PS | L | 0.171 | 0.002 | 457.670 |
| 362 | Gholami et al., 2012 | 29.7 | 52.8 | Ficus carica L. | 14 | 0.7 | Car | NEA | L | 0.301 | 0.003 | 304.122 |
| 363 | Gholami et al., 2012 | 29.7 | 52.8 | Ficus carica L. | 14 | 0.7 | GR | EA | L | -0.273 | 0.001 | 846.784 |
| 364 | Gholami et al., 2012 | 29.7 | 52.8 | Ficus carica L. | 14 | 0.7 | ABA | NEA | L | -0.457 | 0.007 | 146.759 |
| 365 | Gholami et al., 2012 | 29.7 | 52.8 | Ficus carica L. | 14 | 0.7 | CAT | EA | L | -0.383 | 0.024 | 41.520 |
| 366 | Gholami et al., 2012 | 29.7 | 52.8 | Ficus carica L. | 14 | 0.7 | SOD | EA | L | 0.495 | 0.004 | 233.233 |
| 367 | Gokmen et al., 2015 | 32.5 | 37.9 | Cicer arietinum L. | 3 | 0.42 | Chl | PS | L | -0.047 | 0.006 | 169.703 |
| 368 | Gokmen et al., 2015 | 32.5 | 37.9 | Cicer arietinum L. | 5 | 0.53 | Chl | PS | L | -0.104 | 0.006 | 169.703 |
| 369 | Gokmen et al., 2015 | 32.5 | 37.9 | Cicer arietinum L. | 7 | 0.67 | Chl | PS | L | -0.240 | 0.006 | 169.703 |
| 370 | Gokmen et al., 2015 | 32.5 | 37.9 | Cicer arietinum L. | 3 | 0.42 | Chl | PS | L | -0.097 | 0.006 | 169.703 |
| 371 | Gokmen et al., 2015 | 32.5 | 37.9 | Cicer arietinum L. | 5 | 0.53 | Chl | PS | L | -0.215 | 0.006 | 169.703 |
| 372 | Gokmen et al., 2015 | 32.5 | 37.9 | Cicer arietinum L. | 7 | 0.67 | Chl | PS | L | -0.152 | 0.006 | 169.703 |
| 373 | Gokmen et al., 2015 | 32.5 | 37.9 | Cicer arietinum L. | 3 | 0.42 | POD | EA | L | 0.333 | 0.006 | 169.703 |
| 374 | Gokmen et al., 2015 | 32.5 | 37.9 | Cicer arietinum L. | 5 | 0.53 | POD | EA | L | 0.580 | 0.006 | 169.703 |
| 375 | Gokmen et al., 2015 | 32.5 | 37.9 | Cicer arietinum L. | 7 | 0.67 | POD | EA | L | 0.837 | 0.006 | 169.703 |
| 376 | Gokmen et al., 2015 | 32.5 | 37.9 | Cicer arietinum L. | 3 | 0.42 | SOD | EA | L | 1.187 | 0.006 | 169.703 |
| 377 | Gokmen et al., 2015 | 32.5 | 37.9 | Cicer arietinum L. | 5 | 0.53 | SOD | EA | L | 1.260 | 0.006 | 169.703 |
| 378 | Gokmen et al., 2015 | 32.5 | 37.9 | Cicer arietinum L. | 7 | 0.67 | SOD | EA | L | 1.286 | 0.006 | 169.703 |
| 379 | Gokmen et al., 2015 | 32.5 | 37.9 | Cicer arietinum L. | 3 | 0.42 | GR | EA | L | 0.258 | 0.006 | 169.703 |
| 380 | Gokmen et al., 2015 | 32.5 | 37.9 | Cicer arietinum L. | 5 | 0.53 | GR | EA | L | 0.399 | 0.006 | 169.703 |
| 381 | Gokmen et al., 2015 | 32.5 | 37.9 | Cicer arietinum L. | 7 | 0.67 | GR | EA | L | 0.457 | 0.006 | 169.703 |
| 382 | Gokmen et al., 2015 | 32.5 | 37.9 | Cicer arietinum L. | 3 | 0.42 | APX | EA | L | 0.353 | 0.006 | 169.703 |
| 383 | Gokmen et al., 2015 | 32.5 | 37.9 | Cicer arietinum L. | 5 | 0.53 | APX | EA | L | 0.627 | 0.006 | 169.703 |
| 384 | Gokmen et al., 2015 | 32.5 | 37.9 | Cicer arietinum L. | 7 | 0.67 | APX | EA | L | 0.850 | 0.006 | 169.703 |
| 385 | Gokmen et al., 2015 | 32.5 | 37.9 | Cicer arietinum L. | 3 | 0.42 | CAT | EA | L | 0.137 | 0.006 | 169.703 |
| 386 | Gokmen et al., 2015 | 32.5 | 37.9 | Cicer arietinum L. | 5 | 0.53 | CAT | EA | L | 0.232 | 0.006 | 169.703 |
| 387 | Gokmen et al., 2015 | 32.5 | 37.9 | Cicer arietinum L. | 7 | 0.67 | CAT | EA | L | 0.238 | 0.006 | 169.703 |
| 388 | Gokmen et al., 2015 | 32.5 | 37.9 | Cicer arietinum L. | 3 | 0.42 | Proline | NEA | L | 1.863 | 0.006 | 169.703 |
| 389 | Gokmen et al., 2015 | 32.5 | 37.9 | Cicer arietinum L. | 5 | 0.53 | Proline | NEA | L | 1.987 | 0.006 | 169.703 |
| 390 | Gokmen et al., 2015 | 32.5 | 37.9 | Cicer arietinum L. | 7 | 0.67 | Proline | NEA | L | 2.206 | 0.006 | 169.703 |
| 391 | Hameed et al., 2013 | 31.4 | 73 | Triticum aestivum L. | 19 | 0.5 | MDA | PMP | L | 0.248 | 0.003 | 331.254 |
| 392 | Hameed et al., 2013 | 31.4 | 73 | Triticum aestivum L. | 19 | 0.5 | CAT | EA | L | 0.094 | 0.001 | 676.561 |
| 393 | Hameed et al., 2013 | 31.4 | 73 | Triticum aestivum L. | 19 | 0.5 | POD | EA | L | -0.248 | 0.020 | 48.928 |
| 394 | Hameed et al., 2013 | 31.4 | 73 | Triticum aestivum L. | 19 | 0.5 | SOD | EA | L | 0.169 | 0.002 | 500.006 |
| 395 | Hameed et al., 2013 | 31.4 | 73 | Triticum aestivum L. | 19 | 0.5 | APX | EA | L | 0.238 | 0.013 | 74.747 |
| 396 | Hameed et al., 2013 | 31.4 | 73 | Triticum aestivum L. | 19 | 0.5 | Proline | NEA | L | 0.344 | 0.004 | 246.782 |
| 397 | Hameed et al., 2013 | 31.4 | 73 | Triticum aestivum L. | 19 | 0.5 | AsA | NEA | L | -0.248 | 0.014 | 70.280 |
| 398 | Hameed et al., 2013 | 31.4 | 73 | Triticum aestivum L. | 19 | 0.5 | MDA | PMP | L | 0.000 | 0.004 | 274.734 |
| 399 | Hameed et al., 2013 | 31.4 | 73 | Triticum aestivum L. | 19 | 0.5 | CAT | EA | L | 0.103 | 0.002 | 597.761 |
| 400 | Hameed et al., 2013 | 31.4 | 73 | Triticum aestivum L. | 19 | 0.5 | POD | EA | L | 1.041 | 0.030 | 33.391 |
| 401 | Hameed et al., 2013 | 31.4 | 73 | Triticum aestivum L. | 19 | 0.5 | SOD | EA | L | 0.458 | 0.002 | 437.187 |
| 402 | Hameed et al., 2013 | 31.4 | 73 | Triticum aestivum L. | 19 | 0.5 | APX | EA | L | -0.130 | 0.019 | 53.548 |
| 403 | Hameed et al., 2013 | 31.4 | 73 | Triticum aestivum L. | 19 | 0.5 | Proline | NEA | L | 0.026 | 0.001 | 1032.044 |
| 404 | Hameed et al., 2013 | 31.4 | 73 | Triticum aestivum L. | 19 | 0.5 | AsA | NEA | L | 0.087 | 0.020 | 50.054 |
| 405 | Hao et al., 2019 | 34.1 | 108.4 | tomato | 18 | 0.266667 | SOD | EA | L | -0.392 | 0.000 | 11092.163 |
| 406 | Hao et al., 2019 | 34.1 | 108.4 | tomato | 18 | 0.4 | SOD | EA | L | 0.490 | 0.000 | 29236.070 |
| 407 | Hao et al., 2019 | 34.1 | 108.4 | tomato | 18 | 0.266667 | SOD | EA | R | 0.129 | 0.000 | 36906.342 |
| 408 | Hao et al., 2019 | 34.1 | 108.4 | tomato | 18 | 0.4 | SOD | EA | R | 0.143 | 0.000 | 70378.707 |
| 409 | Hao et al., 2019 | 34.1 | 108.4 | tomato | 18 | 0.266667 | POD | EA | L | 0.707 | 0.001 | 1533.124 |
| 410 | Hao et al., 2019 | 34.1 | 108.4 | tomato | 18 | 0.4 | POD | EA | L | 1.718 | 0.000 | 2196.876 |
| 411 | Hao et al., 2019 | 34.1 | 108.4 | tomato | 18 | 0.266667 | POD | EA | R | -0.037 | 0.001 | 1697.030 |
| 412 | Hao et al., 2019 | 34.1 | 108.4 | tomato | 18 | 0.4 | POD | EA | R | 0.081 | 0.001 | 1697.030 |
| 413 | Hao et al., 2019 | 34.1 | 108.4 | tomato | 18 | 0.27 | CAT | EA | L | 0.053 | 0.000 | 6905.505 |
| 414 | Hao et al., 2019 | 34.1 | 108.4 | tomato | 18 | 0.4 | CAT | EA | L | 0.620 | 0.000 | 9087.373 |
| 415 | Hao et al., 2019 | 34.1 | 108.4 | tomato | 18 | 0.27 | CAT | EA | R | 0.049 | 0.000 | 13052.691 |
| 416 | Hao et al., 2019 | 34.1 | 108.4 | tomato | 18 | 0.4 | CAT | EA | R | 0.147 | 0.000 | 11768.467 |
| 417 | He et al., 2019 | 39.9 | 116.5 | Glycyrrhiza uralensis | 60 | 0.57 | Dry weight | Growth | S | -0.345 | 0.001 | 1146.186 |
| 418 | He et al., 2019 | 39.9 | 116.5 | Glycyrrhiza uralensis | 60 | 0.57 | Dry weight | Growth | R | -0.011 | 0.000 | 2150.903 |
| 419 | He et al., 2019 | 39.9 | 116.5 | Glycyrrhiza uralensis | 60 | 0.57 | MDA | PMP | L | 0.344 | 0.010 | 105.006 |
| 420 | He et al., 2019 | 39.9 | 116.5 | Glycyrrhiza uralensis | 60 | 0.57 | SOD | EA | L | -0.189 | 0.003 | 293.756 |
| 421 | He et al., 2019 | 39.9 | 116.5 | Glycyrrhiza uralensis | 60 | 0.57 | Protein | Growth | L | 0.142 | 0.009 | 116.807 |
| 422 | He et al., 2019 | 39.9 | 116.5 | Glycyrrhiza uralensis | 60 | 0.57 | CAT | EA | L | -0.182 | 0.006 | 169.811 |
| 423 | He et al., 2019 | 39.9 | 116.5 | Glycyrrhiza uralensis | 60 | 0.57 | POD | EA | L | 0.194 | 0.002 | 583.959 |
| 424 | Hosseini et al., 2015 | 35.8 | 50.9 | Brassica napus L. | 65 | 0.333333 | Dry weight | Growth | P | -0.355 | 0.004 | 226.271 |
| 425 | Hosseini et al., 2015 | 35.8 | 50.9 | Brassica napus L. | 65 | 0.67 | Dry weight | Growth | P | -0.630 | 0.004 | 226.271 |
| 426 | Hosseini et al., 2015 | 35.8 | 50.9 | Brassica napus L. | 65 | 0.33 | Dry weight | Growth | P | -0.355 | 0.004 | 226.271 |
| 427 | Hosseini et al., 2015 | 35.8 | 50.9 | Brassica napus L. | 65 | 0.666667 | Dry weight | Growth | P | -0.545 | 0.004 | 226.271 |
| 428 | Hosseini et al., 2015 | 35.8 | 50.9 | Brassica napus L. | 65 | 0.333333 | Proline | NEA | L | 0.418 | 0.004 | 226.271 |
| 429 | Hosseini et al., 2015 | 35.8 | 50.9 | Brassica napus L. | 65 | 0.67 | Proline | NEA | L | 1.045 | 0.004 | 226.271 |
| 430 | Hosseini et al., 2015 | 35.8 | 50.9 | Brassica napus L. | 65 | 0.33 | Proline | NEA | L | 1.338 | 0.004 | 226.271 |
| 431 | Hosseini et al., 2015 | 35.8 | 50.9 | Brassica napus L. | 65 | 0.666667 | Proline | NEA | L | 1.819 | 0.004 | 226.271 |
| 432 | Hosseini et al., 2015 | 35.8 | 50.9 | Brassica napus L. | 65 | 0.333333 | CAT | EA | L | 0.475 | 0.004 | 226.271 |
| 433 | Hosseini et al., 2015 | 35.8 | 50.9 | Brassica napus L. | 65 | 0.67 | CAT | EA | L | 0.502 | 0.004 | 226.271 |
| 434 | Hosseini et al., 2015 | 35.8 | 50.9 | Brassica napus L. | 65 | 0.33 | CAT | EA | L | 0.462 | 0.004 | 226.271 |
| 435 | Hosseini et al., 2015 | 35.8 | 50.9 | Brassica napus L. | 65 | 0.666667 | CAT | EA | L | 0.641 | 0.004 | 226.271 |
| 436 | Hosseini et al., 2015 | 35.8 | 50.9 | Brassica napus L. | 65 | 0.333333 | APX | EA | L | 0.272 | 0.004 | 226.271 |
| 437 | Hosseini et al., 2015 | 35.8 | 50.9 | Brassica napus L. | 65 | 0.67 | APX | EA | L | 0.241 | 0.004 | 226.271 |
| 438 | Hosseini et al., 2015 | 35.8 | 50.9 | Brassica napus L. | 65 | 0.33 | APX | EA | L | 0.277 | 0.004 | 226.271 |
| 439 | Hosseini et al., 2015 | 35.8 | 50.9 | Brassica napus L. | 65 | 0.666667 | APX | EA | L | 0.344 | 0.004 | 226.271 |
| 440 | Hosseini et al., 2015 | 35.8 | 50.9 | Brassica napus L. | 65 | 0.333333 | POD | EA | L | 0.204 | 0.004 | 226.271 |
| 441 | Hosseini et al., 2015 | 35.8 | 50.9 | Brassica napus L. | 65 | 0.67 | POD | EA | L | 0.982 | 0.004 | 226.271 |
| 442 | Hosseini et al., 2015 | 35.8 | 50.9 | Brassica napus L. | 65 | 0.33 | POD | EA | L | 0.725 | 0.004 | 226.271 |
| 443 | Hosseini et al., 2015 | 35.8 | 50.9 | Brassica napus L. | 65 | 0.666667 | POD | EA | L | 1.001 | 0.004 | 226.271 |
| 444 | Hu et al., 2010 | 27.8 | 115.4 | Capsicum annuum L. | 5 | 0.375 | EL | PMP | L | 0.368 | 0.012 | 84.321 |
| 445 | Hu et al., 2010 | 27.8 | 115.4 | Capsicum annuum L. | 5 | 0.375 | EL | PMP | L | 0.565 | 0.010 | 104.519 |
| 446 | Hu et al., 2010 | 27.8 | 115.4 | Capsicum annuum L. | 5 | 0.375 | Fv/Fm | PS | L | -0.084 | 0.000 | 7101.999 |
| 447 | Hu et al., 2010 | 27.8 | 115.4 | Capsicum annuum L. | 5 | 0.375 | Fv/Fm | PS | L | -0.246 | 0.000 | 17940.139 |
| 448 | Hu et al., 2010 | 27.8 | 115.4 | Capsicum annuum L. | 5 | 0.375 | ROS | PMP | L | 0.501 | 0.048 | 21.013 |
| 449 | Hu et al., 2010 | 27.8 | 115.4 | Capsicum annuum L. | 5 | 0.375 | ROS | PMP | L | 0.784 | 0.029 | 34.056 |
| 450 | Hu et al., 2010 | 27.8 | 115.4 | Capsicum annuum L. | 5 | 0.375 | ROS | PMP | L | -1.945 | 1.336 | 0.748 |
| 451 | Hu et al., 2010 | 27.8 | 115.4 | Capsicum annuum L. | 5 | 0.375 | ROS | PMP | L | -2.246 | 0.541 | 1.849 |
| 452 | Hu et al., 2010 | 27.8 | 115.4 | Capsicum annuum L. | 5 | 0.375 | MDA | PMP | L | 0.786 | 0.005 | 220.885 |
| 453 | Hu et al., 2010 | 27.8 | 115.4 | Capsicum annuum L. | 5 | 0.375 | MDA | PMP | L | 1.253 | 0.006 | 172.696 |
| 454 | Hu et al., 2010 | 27.8 | 115.4 | Capsicum annuum L. | 5 | 0.375 | SOD | EA | L | 0.985 | 0.017 | 59.902 |
| 455 | Hu et al., 2010 | 27.8 | 115.4 | Capsicum annuum L. | 5 | 0.375 | SOD | EA | L | 0.716 | 0.017 | 59.557 |
| 456 | Hu et al., 2010 | 27.8 | 115.4 | Capsicum annuum L. | 5 | 0.375 | APX | EA | L | 0.718 | 0.007 | 133.775 |
| 457 | Hu et al., 2010 | 27.8 | 115.4 | Capsicum annuum L. | 5 | 0.375 | APX | EA | L | 0.762 | 0.012 | 85.354 |
| 458 | Hu et al., 2010 | 27.8 | 115.4 | Capsicum annuum L. | 5 | 0.375 | SOD | EA | L | 1.014 | 0.009 | 107.234 |
| 459 | Hu et al., 2010 | 27.8 | 115.4 | Capsicum annuum L. | 5 | 0.375 | SOD | EA | L | 0.723 | 0.006 | 159.414 |
| 460 | Hu et al., 2010 | 27.8 | 115.4 | Capsicum annuum L. | 5 | 0.375 | APX | EA | L | 1.175 | 0.009 | 117.302 |
| 461 | Hu et al., 2010 | 27.8 | 115.4 | Capsicum annuum L. | 5 | 0.375 | APX | EA | L | 0.797 | 0.005 | 181.842 |
| 462 | Hu et al., 2010 | 27.8 | 115.4 | Capsicum annuum L. | 5 | 0.375 | SOD | EA | L | 1.172 | 0.010 | 99.401 |
| 463 | Hu et al., 2010 | 27.8 | 115.4 | Capsicum annuum L. | 5 | 0.375 | SOD | EA | L | 0.711 | 0.009 | 113.656 |
| 464 | Hu et al., 2010 | 27.8 | 115.4 | Capsicum annuum L. | 5 | 0.375 | APX | EA | L | 1.286 | 0.008 | 117.803 |
| 465 | Hu et al., 2010 | 27.8 | 115.4 | Capsicum annuum L. | 5 | 0.375 | APX | EA | L | 0.887 | 0.008 | 119.161 |
| 466 | Husen et al., 2010 | 30.3 | 77.9 | Tectona Grandis Linn. f. | 20 | 0.5 | Chl | PS | L | -0.651 | 0.003 | 374.178 |
| 467 | Husen et al., 2010 | 30.3 | 77.9 | Tectona Grandis Linn. f. | 20 | 0.5 | Chl | PS | L | -0.671 | 0.010 | 104.159 |
| 468 | Husen et al., 2010 | 30.3 | 77.9 | Tectona Grandis Linn. f. | 20 | 0.5 | Chl | PS | L | -0.666 | 0.003 | 354.018 |
| 469 | Husen et al., 2010 | 30.3 | 77.9 | Tectona Grandis Linn. f. | 20 | 0.5 | Chl | PS | L | -0.693 | 0.005 | 221.918 |
| 470 | Husen et al., 2010 | 30.3 | 77.9 | Tectona Grandis Linn. f. | 20 | 0.5 | Car | NEA | L | 0.577 | 0.003 | 332.476 |
| 471 | Husen et al., 2010 | 30.3 | 77.9 | Tectona Grandis Linn. f. | 20 | 0.5 | Car | NEA | L | 0.560 | 0.003 | 320.634 |
| 472 | Husen et al., 2010 | 30.3 | 77.9 | Tectona Grandis Linn. f. | 20 | 0.5 | Fv/Fm | PS | L | -0.257 | 0.000 | 6436.907 |
| 473 | Husen et al., 2010 | 30.3 | 77.9 | Tectona Grandis Linn. f. | 20 | 0.5 | Fv/Fm | PS | L | -0.266 | 0.000 | 3222.988 |
| 474 | Husen et al., 2010 | 30.3 | 77.9 | Tectona Grandis Linn. f. | 20 | 0.5 | Proline | NEA | L | -0.013 | 0.003 | 379.341 |
| 475 | Husen et al., 2010 | 30.3 | 77.9 | Tectona Grandis Linn. f. | 20 | 0.5 | EL | PMP | L | 0.113 | 0.002 | 573.581 |
| 476 | Husen et al., 2010 | 30.3 | 77.9 | Tectona Grandis Linn. f. | 20 | 0.5 | MDA | PMP | L | 0.044 | 0.007 | 145.130 |
| 477 | Husen et al., 2010 | 30.3 | 77.9 | Tectona Grandis Linn. f. | 20 | 0.5 | SOD | EA | L | -0.031 | 0.004 | 268.401 |
| 478 | Huseynova et al., 2015 | 40.5 | 47.7 | Triticum durum Desf. | 60 | 0.5 | Protein | Growth | L | 1.069 | 0.007 | 153.222 |
| 479 | Huseynova et al., 2015 | 40.5 | 47.7 | Triticum durum Desf. | 60 | 0.5 | Protein | Growth | L | 0.542 | 0.006 | 174.339 |
| 480 | Huseynova et al., 2015 | 40.5 | 47.7 | Triticum durum Desf. | 60 | 0.5 | Protein | Growth | L | 0.881 | 0.005 | 199.712 |
| 481 | Huseynova et al., 2015 | 40.5 | 47.7 | Triticum durum Desf. | 60 | 0.5 | Protein | Growth | R | 0.120 | 0.006 | 177.529 |
| 482 | Huseynova et al., 2015 | 40.5 | 47.7 | Triticum durum Desf. | 60 | 0.5 | Protein | Growth | R | 0.739 | 0.012 | 84.166 |
| 483 | Huseynova et al., 2015 | 40.5 | 47.7 | Triticum durum Desf. | 60 | 0.5 | Protein | Growth | R | 0.933 | 0.008 | 126.460 |
| 484 | Huseynova et al., 2015 | 40.5 | 47.7 | Triticum durum Desf. | 60 | 0.5 | CAT | EA | L | 0.199 | 0.002 | 471.152 |
| 485 | Huseynova et al., 2015 | 40.5 | 47.7 | Triticum durum Desf. | 60 | 0.5 | CAT | EA | L | 0.087 | 0.003 | 379.951 |
| 486 | Huseynova et al., 2015 | 40.5 | 47.7 | Triticum durum Desf. | 60 | 0.5 | CAT | EA | L | 0.552 | 0.003 | 392.433 |
| 487 | Huseynova et al., 2015 | 40.5 | 47.7 | Triticum durum Desf. | 60 | 0.5 | CAT | EA | R | -0.425 | 0.002 | 424.069 |
| 488 | Huseynova et al., 2015 | 40.5 | 47.7 | Triticum durum Desf. | 60 | 0.5 | CAT | EA | R | -0.400 | 0.022 | 44.659 |
| 489 | Huseynova et al., 2015 | 40.5 | 47.7 | Triticum durum Desf. | 60 | 0.5 | CAT | EA | R | 0.264 | 0.009 | 106.386 |
| 490 | Jafari et al., 2019 | 35.5 | 51.7 | Matthiola incana L. | 193 | 0.1 | Dry weight | Growth | S | -0.076 | 0.002 | 494.800 |
| 491 | Jafari et al., 2019 | 35.5 | 51.7 | Matthiola incana L. | 193 | 0.2 | Dry weight | Growth | S | -0.308 | 0.003 | 394.049 |
| 492 | Jafari et al., 2019 | 35.5 | 51.7 | Matthiola incana L. | 193 | 0.3 | Dry weight | Growth | S | -0.620 | 0.003 | 332.426 |
| 493 | Jafari et al., 2019 | 35.5 | 51.7 | Matthiola incana L. | 193 | 0.4 | Dry weight | Growth | S | -1.008 | 0.003 | 384.007 |
| 494 | Jafari et al., 2019 | 35.5 | 51.7 | Matthiola incana L. | 193 | 0.1 | Dry weight | Growth | R | -0.289 | 0.005 | 193.963 |
| 495 | Jafari et al., 2019 | 35.5 | 51.7 | Matthiola incana L. | 193 | 0.2 | Dry weight | Growth | R | -0.342 | 0.006 | 163.661 |
| 496 | Jafari et al., 2019 | 35.5 | 51.7 | Matthiola incana L. | 193 | 0.3 | Dry weight | Growth | R | -0.441 | 0.008 | 123.234 |
| 497 | Jafari et al., 2019 | 35.5 | 51.7 | Matthiola incana L. | 193 | 0.4 | Dry weight | Growth | R | -0.691 | 0.013 | 79.755 |
| 498 | Jafari et al., 2019 | 35.5 | 51.7 | Matthiola incana L. | 193 | 0.1 | CAT | EA | L | 0.087 | 0.004 | 226.271 |
| 499 | Jafari et al., 2019 | 35.5 | 51.7 | Matthiola incana L. | 193 | 0.2 | CAT | EA | L | 0.087 | 0.004 | 226.271 |
| 500 | Jafari et al., 2019 | 35.5 | 51.7 | Matthiola incana L. | 193 | 0.3 | CAT | EA | L | 0.087 | 0.004 | 226.271 |
| 501 | Jafari et al., 2019 | 35.5 | 51.7 | Matthiola incana L. | 193 | 0.4 | CAT | EA | L | 0.128 | 0.004 | 226.271 |
| 502 | Jafari et al., 2019 | 35.5 | 51.7 | Matthiola incana L. | 193 | 0.1 | Proline | NEA | L | 0.097 | 0.004 | 226.271 |
| 503 | Jafari et al., 2019 | 35.5 | 51.7 | Matthiola incana L. | 193 | 0.2 | Proline | NEA | L | 0.160 | 0.004 | 226.271 |
| 504 | Jafari et al., 2019 | 35.5 | 51.7 | Matthiola incana L. | 193 | 0.3 | Proline | NEA | L | 0.169 | 0.004 | 226.271 |
| 505 | Jafari et al., 2019 | 35.5 | 51.7 | Matthiola incana L. | 193 | 0.4 | Proline | NEA | L | 0.181 | 0.004 | 226.271 |
| 506 | Ji et al., 2014 | 30.2 | 102.7 | Dactylis glomerata L. | 21 | 0.428571 | MDA | PMP | L | 1.651 | 0.004 | 226.271 |
| 507 | Ji et al., 2014 | 30.2 | 102.7 | Dactylis glomerata L. | 24 | 0.428571 | MDA | PMP | L | 1.111 | 0.004 | 226.271 |
| 508 | Ji et al., 2014 | 30.2 | 102.7 | Dactylis glomerata L. | 9 | 0.428571 | EL | PMP | L | 0.262 | 0.004 | 226.271 |
| 509 | Ji et al., 2014 | 30.2 | 102.7 | Dactylis glomerata L. | 9 | 0.428571 | EL | PMP | L | 0.252 | 0.004 | 226.271 |
| 510 | Ji et al., 2014 | 30.2 | 102.7 | Dactylis glomerata L. | 18 | 0.428571 | EL | PMP | L | 1.066 | 0.004 | 226.271 |
| 511 | Ji et al., 2014 | 30.2 | 102.7 | Dactylis glomerata L. | 18 | 0.428571 | EL | PMP | L | 0.766 | 0.004 | 226.271 |
| 512 | Ji et al., 2014 | 30.2 | 102.7 | Dactylis glomerata L. | 21 | 0.428571 | EL | PMP | L | 1.225 | 0.004 | 226.271 |
| 513 | Ji et al., 2014 | 30.2 | 102.7 | Dactylis glomerata L. | 21 | 0.428571 | EL | PMP | L | 0.957 | 0.004 | 226.271 |
| 514 | Ji et al., 2014 | 30.2 | 102.7 | Dactylis glomerata L. | 21 | 0.428571 | Proline | NEA | L | 2.547 | 0.004 | 226.271 |
| 515 | Ji et al., 2014 | 30.2 | 102.7 | Dactylis glomerata L. | 24 | 0.428571 | Proline | NEA | L | 3.583 | 0.004 | 226.271 |
| 516 | Ji et al., 2014 | 30.2 | 102.7 | Dactylis glomerata L. | 21 | 0.428571 | SOD | EA | L | -0.437 | 0.025 | 40.620 |
| 517 | Ji et al., 2014 | 30.2 | 102.7 | Dactylis glomerata L. | 24 | 0.428571 | SOD | EA | L | -0.548 | 0.100 | 10.040 |
| 518 | Ji et al., 2014 | 30.2 | 102.7 | Dactylis glomerata L. | 21 | 0.428571 | POD | EA | L | 0.000 | 0.003 | 358.871 |
| 519 | Ji et al., 2014 | 30.2 | 102.7 | Dactylis glomerata L. | 24 | 0.428571 | POD | EA | L | 0.013 | 0.005 | 210.745 |
| 520 | Ji et al., 2014 | 30.2 | 102.7 | Dactylis glomerata L. | 21 | 0.428571 | CAT | EA | L | -0.718 | 0.001 | 1114.091 |
| 521 | Ji et al., 2014 | 30.2 | 102.7 | Dactylis glomerata L. | 24 | 0.428571 | CAT | EA | L | -0.423 | 0.001 | 776.217 |
| 522 | Junior et al., 2019 | -9.5 | -35.8 | Saccharum officinarum | 22 | 0.5 | Chl | PS | L | -0.155 | 0.003 | 339.406 |
| 523 | Junior et al., 2019 | -9.5 | -35.8 | Saccharum officinarum | 22 | 0.85 | Chl | PS | L | -0.242 | 0.004 | 226.271 |
| 524 | Junior et al., 2019 | -9.5 | -35.8 | Saccharum officinarum | 22 | 0.5 | Chl | PS | L | -0.283 | 0.004 | 226.271 |
| 525 | Junior et al., 2019 | -9.5 | -35.8 | Saccharum officinarum | 22 | 0.85 | Chl | PS | L | -0.384 | 0.004 | 226.271 |
| 526 | Junior et al., 2019 | -9.5 | -35.8 | Saccharum officinarum | 22 | 0.5 | Car | PS | L | -0.148 | 0.004 | 226.271 |
| 527 | Junior et al., 2019 | -9.5 | -35.8 | Saccharum officinarum | 22 | 0.85 | Car | PS | L | -0.308 | 0.004 | 226.271 |
| 528 | Junior et al., 2019 | -9.5 | -35.8 | Saccharum officinarum | 22 | 0.5 | Fv/Fm | PS | L | -0.026 | 0.004 | 226.271 |
| 529 | Junior et al., 2019 | -9.5 | -35.8 | Saccharum officinarum | 22 | 0.85 | Fv/Fm | PS | L | -0.134 | 0.004 | 226.271 |
| 530 | Junior et al., 2019 | -9.5 | -35.8 | Saccharum officinarum | 22 | 0.5 | CAT | EA | L | 0.338 | 0.004 | 226.271 |
| 531 | Junior et al., 2019 | -9.5 | -35.8 | Saccharum officinarum | 22 | 0.85 | CAT | EA | L | 0.455 | 0.004 | 226.271 |
| 532 | Junior et al., 2019 | -9.5 | -35.8 | Saccharum officinarum | 22 | 0.5 | SOD | EA | L | 0.433 | 0.004 | 226.271 |
| 533 | Junior et al., 2019 | -9.5 | -35.8 | Saccharum officinarum | 22 | 0.85 | SOD | EA | L | 0.585 | 0.004 | 226.271 |
| 534 | Junior et al., 2019 | -9.5 | -35.8 | Saccharum officinarum | 22 | 0.5 | ROS | PMP | L | 0.153 | 0.004 | 226.271 |
| 535 | Junior et al., 2019 | -9.5 | -35.8 | Saccharum officinarum | 22 | 0.85 | ROS | PMP | L | 0.539 | 0.004 | 226.271 |
| 536 | Junior et al., 2019 | -9.5 | -35.8 | Saccharum officinarum | 22 | 0.5 | MDA | PMP | L | 0.140 | 0.004 | 226.271 |
| 537 | Junior et al., 2019 | -9.5 | -35.8 | Saccharum officinarum | 22 | 0.85 | MDA | PMP | L | 0.416 | 0.004 | 226.271 |
| 538 | Karatas et al., 2014 | 40.1 | 38.1 | Pisum sativum L. | 42 | 0.25 | Chl | PS | L | -0.050 | 0.000 | 4993.833 |
| 539 | Karatas et al., 2014 | 40.1 | 38.1 | Pisum sativum L. | 42 | 0.5 | Chl | PS | L | -0.243 | 0.001 | 832.679 |
| 540 | Karatas et al., 2014 | 40.1 | 38.1 | Pisum sativum L. | 42 | 0.25 | Protein | Growth | L | -0.139 | 0.001 | 881.216 |
| 541 | Karatas et al., 2014 | 40.1 | 38.1 | Pisum sativum L. | 42 | 0.5 | Protein | Growth | L | -0.208 | 0.001 | 1158.383 |
| 542 | Karatas et al., 2014 | 40.1 | 38.1 | Pisum sativum L. | 42 | 0.25 | Proline | NEA | L | 0.161 | 0.001 | 840.036 |
| 543 | Karatas et al., 2014 | 40.1 | 38.1 | Pisum sativum L. | 42 | 0.5 | Proline | NEA | L | 0.472 | 0.001 | 714.322 |
| 544 | Karatas et al., 2014 | 40.1 | 38.1 | Pisum sativum L. | 42 | 0.25 | ROS | PMP | L | 0.092 | 0.001 | 1889.704 |
| 545 | Karatas et al., 2014 | 40.1 | 38.1 | Pisum sativum L. | 42 | 0.5 | ROS | PMP | L | 0.036 | 0.002 | 488.058 |
| 546 | Karatas et al., 2014 | 40.1 | 38.1 | Pisum sativum L. | 42 | 0.25 | MDA | PMP | L | 0.246 | 0.002 | 520.807 |
| 547 | Karatas et al., 2014 | 40.1 | 38.1 | Pisum sativum L. | 42 | 0.5 | MDA | PMP | L | 0.101 | 0.002 | 529.138 |
| 548 | Karatas et al., 2014 | 40.1 | 38.1 | Pisum sativum L. | 42 | 0.25 | SOD | EA | L | 0.080 | 0.006 | 154.565 |
| 549 | Karatas et al., 2014 | 40.1 | 38.1 | Pisum sativum L. | 42 | 0.5 | SOD | EA | L | 0.278 | 0.007 | 143.614 |
| 550 | Karatas et al., 2014 | 40.1 | 38.1 | Pisum sativum L. | 42 | 0.25 | CAT | EA | L | 0.499 | 0.004 | 236.751 |
| 551 | Karatas et al., 2014 | 40.1 | 38.1 | Pisum sativum L. | 42 | 0.5 | CAT | EA | L | 0.600 | 0.004 | 233.977 |
| 552 | Karatas et al., 2014 | 40.1 | 38.1 | Pisum sativum L. | 42 | 0.25 | POD | EA | L | 0.331 | 0.003 | 339.218 |
| 553 | Karatas et al., 2014 | 40.1 | 38.1 | Pisum sativum L. | 42 | 0.5 | POD | EA | L | 0.181 | 0.009 | 108.148 |
| 554 | Karatas et al., 2014 | 40.1 | 38.1 | Pisum sativum L. | 42 | 0.25 | APX | EA | L | 0.062 | 0.002 | 487.165 |
| 555 | Karatas et al., 2014 | 40.1 | 38.1 | Pisum sativum L. | 42 | 0.5 | APX | EA | L | 0.082 | 0.001 | 1194.513 |
| 556 | Kebbas et al., 2018 | 35.2 | -0.6 | Gleditsia triacanthos L. | 1 | 0.33 | Chl | PS | L | 0.000 | 0.003 | 335.406 |
| 557 | Kebbas et al., 2018 | 35.2 | -0.6 | Gleditsia triacanthos L. | 4 | 0.33 | Chl | PS | L | -0.152 | 0.003 | 309.567 |
| 558 | Kebbas et al., 2018 | 35.2 | -0.6 | Gleditsia triacanthos L. | 9 | 0.33 | Chl | PS | L | -0.327 | 0.002 | 543.148 |
| 559 | Kebbas et al., 2018 | 35.2 | -0.6 | Gleditsia triacanthos L. | 13 | 0.33 | Chl | PS | L | -0.642 | 0.006 | 158.451 |
| 560 | Kebbas et al., 2018 | 35.2 | -0.6 | Gleditsia triacanthos L. | 16 | 0.33 | Chl | PS | L | -2.093 | 0.005 | 216.762 |
| 561 | Kebbas et al., 2018 | 35.2 | -0.6 | Gleditsia triacanthos L. | 19 | 0.33 | Chl | PS | L | -2.442 | 0.004 | 272.263 |
| 562 | Kebbas et al., 2018 | 35.2 | -0.6 | Gleditsia triacanthos L. | 1 | 0.33 | Car | NEA | L | 0.000 | 0.010 | 95.400 |
| 563 | Kebbas et al., 2018 | 35.2 | -0.6 | Gleditsia triacanthos L. | 4 | 0.33 | Car | NEA | L | -0.157 | 0.006 | 167.007 |
| 564 | Kebbas et al., 2018 | 35.2 | -0.6 | Gleditsia triacanthos L. | 9 | 0.33 | Car | NEA | L | 0.028 | 0.002 | 433.535 |
| 565 | Kebbas et al., 2018 | 35.2 | -0.6 | Gleditsia triacanthos L. | 13 | 0.33 | Car | NEA | L | -0.003 | 0.078 | 12.794 |
| 566 | Kebbas et al., 2018 | 35.2 | -0.6 | Gleditsia triacanthos L. | 16 | 0.33 | Car | NEA | L | 0.162 | 0.010 | 104.731 |
| 567 | Kebbas et al., 2018 | 35.2 | -0.6 | Gleditsia triacanthos L. | 19 | 0.33 | Car | NEA | L | 0.200 | 0.008 | 119.304 |
| 568 | Kebbas et al., 2018 | 35.2 | -0.6 | Gleditsia triacanthos L. | 1 | 0.33 | Car | NEA | L | -0.058 | 0.004 | 282.838 |
| 569 | Kebbas et al., 2018 | 35.2 | -0.6 | Gleditsia triacanthos L. | 4 | 0.33 | Car | NEA | L | 1.332 | 0.004 | 282.838 |
| 570 | Kebbas et al., 2018 | 35.2 | -0.6 | Gleditsia triacanthos L. | 9 | 0.33 | Car | NEA | L | 1.717 | 0.004 | 282.838 |
| 571 | Kebbas et al., 2018 | 35.2 | -0.6 | Gleditsia triacanthos L. | 13 | 0.33 | Car | NEA | L | 1.961 | 0.004 | 282.838 |
| 572 | Kebbas et al., 2018 | 35.2 | -0.6 | Gleditsia triacanthos L. | 16 | 0.33 | Car | NEA | L | 2.560 | 0.004 | 282.838 |
| 573 | Kebbas et al., 2018 | 35.2 | -0.6 | Gleditsia triacanthos L. | 19 | 0.33 | Car | NEA | L | 2.737 | 0.004 | 282.838 |
| 574 | Khaleghi et al., 2019 | 35.8 | 50.9 | Maclura pomifera (Raf.) | 22 | 0.25 | Dry weight | Growth | P | -0.253 | 0.002 | 449.787 |
| 575 | Khaleghi et al., 2019 | 35.8 | 50.9 | Maclura pomifera (Raf.) | 22 | 0.5 | Dry weight | Growth | P | -0.419 | 0.003 | 292.752 |
| 576 | Khaleghi et al., 2019 | 35.8 | 50.9 | Maclura pomifera (Raf.) | 22 | 0.7 | Dry weight | Growth | P | -0.766 | 0.003 | 337.108 |
| 577 | Khaleghi et al., 2019 | 35.8 | 50.9 | Maclura pomifera (Raf.) | 1 | 0.25 | Dry weight | Growth | P | -0.141 | 0.015 | 66.926 |
| 578 | Khaleghi et al., 2019 | 35.8 | 50.9 | Maclura pomifera (Raf.) | 8 | 0.25 | Dry weight | Growth | P | -0.296 | 0.007 | 140.722 |
| 579 | Khaleghi et al., 2019 | 35.8 | 50.9 | Maclura pomifera (Raf.) | 15 | 0.25 | Dry weight | Growth | P | -0.261 | 0.012 | 82.613 |
| 580 | Khaleghi et al., 2019 | 35.8 | 50.9 | Maclura pomifera (Raf.) | 22 | 0.25 | Dry weight | Growth | P | -0.361 | 0.028 | 36.319 |
| 581 | Khaleghi et al., 2019 | 35.8 | 50.9 | Maclura pomifera (Raf.) | 1 | 0.5 | Dry weight | Growth | P | -0.220 | 0.021 | 48.211 |
| 582 | Khaleghi et al., 2019 | 35.8 | 50.9 | Maclura pomifera (Raf.) | 8 | 0.5 | Dry weight | Growth | P | -0.279 | 0.029 | 34.263 |
| 583 | Khaleghi et al., 2019 | 35.8 | 50.9 | Maclura pomifera (Raf.) | 15 | 0.5 | Dry weight | Growth | P | -0.477 | 0.023 | 43.221 |
| 584 | Khaleghi et al., 2019 | 35.8 | 50.9 | Maclura pomifera (Raf.) | 22 | 0.5 | Dry weight | Growth | P | -0.606 | 0.034 | 29.148 |
| 585 | Khaleghi et al., 2019 | 35.8 | 50.9 | Maclura pomifera (Raf.) | 1 | 0.7 | Dry weight | Growth | P | -0.481 | 0.018 | 55.255 |
| 586 | Khaleghi et al., 2019 | 35.8 | 50.9 | Maclura pomifera (Raf.) | 8 | 0.7 | Dry weight | Growth | P | -0.643 | 0.035 | 28.517 |
| 587 | Khaleghi et al., 2019 | 35.8 | 50.9 | Maclura pomifera (Raf.) | 15 | 0.7 | Dry weight | Growth | P | -0.828 | 0.035 | 28.472 |
| 588 | Khaleghi et al., 2019 | 35.8 | 50.9 | Maclura pomifera (Raf.) | 22 | 0.7 | Dry weight | Growth | P | -0.882 | 0.016 | 62.313 |
| 589 | Khaleghi et al., 2019 | 35.8 | 50.9 | Maclura pomifera (Raf.) | 1 | 0.25 | MDA | PMP | P | 0.257 | 0.008 | 122.429 |
| 590 | Khaleghi et al., 2019 | 35.8 | 50.9 | Maclura pomifera (Raf.) | 8 | 0.25 | MDA | PMP | P | 0.292 | 0.008 | 123.762 |
| 591 | Khaleghi et al., 2019 | 35.8 | 50.9 | Maclura pomifera (Raf.) | 15 | 0.25 | MDA | PMP | P | 0.240 | 0.008 | 131.206 |
| 592 | Khaleghi et al., 2019 | 35.8 | 50.9 | Maclura pomifera (Raf.) | 22 | 0.25 | MDA | PMP | P | 0.472 | 0.017 | 57.398 |
| 593 | Khaleghi et al., 2019 | 35.8 | 50.9 | Maclura pomifera (Raf.) | 1 | 0.5 | MDA | PMP | P | 0.605 | 0.007 | 152.455 |
| 594 | Khaleghi et al., 2019 | 35.8 | 50.9 | Maclura pomifera (Raf.) | 8 | 0.5 | MDA | PMP | P | 0.729 | 0.005 | 183.064 |
| 595 | Khaleghi et al., 2019 | 35.8 | 50.9 | Maclura pomifera (Raf.) | 15 | 0.5 | MDA | PMP | P | 0.547 | 0.007 | 144.301 |
| 596 | Khaleghi et al., 2019 | 35.8 | 50.9 | Maclura pomifera (Raf.) | 22 | 0.5 | MDA | PMP | P | 0.650 | 0.016 | 61.496 |
| 597 | Khaleghi et al., 2019 | 35.8 | 50.9 | Maclura pomifera (Raf.) | 1 | 0.7 | MDA | PMP | P | 1.093 | 0.005 | 182.717 |
| 598 | Khaleghi et al., 2019 | 35.8 | 50.9 | Maclura pomifera (Raf.) | 8 | 0.7 | MDA | PMP | P | 1.271 | 0.005 | 216.257 |
| 599 | Khaleghi et al., 2019 | 35.8 | 50.9 | Maclura pomifera (Raf.) | 15 | 0.7 | MDA | PMP | P | 1.184 | 0.006 | 178.105 |
| 600 | Khaleghi et al., 2019 | 35.8 | 50.9 | Maclura pomifera (Raf.) | 22 | 0.7 | MDA | PMP | P | 1.377 | 0.015 | 65.034 |
| 601 | Khaleghi et al., 2019 | 35.8 | 50.9 | Maclura pomifera (Raf.) | 1 | 0.25 | Proline | NEA | P | -0.189 | 0.007 | 138.019 |
| 602 | Khaleghi et al., 2019 | 35.8 | 50.9 | Maclura pomifera (Raf.) | 8 | 0.25 | Proline | NEA | P | -0.116 | 0.006 | 169.543 |
| 603 | Khaleghi et al., 2019 | 35.8 | 50.9 | Maclura pomifera (Raf.) | 15 | 0.25 | Proline | NEA | P | -0.074 | 0.005 | 197.000 |
| 604 | Khaleghi et al., 2019 | 35.8 | 50.9 | Maclura pomifera (Raf.) | 22 | 0.25 | Proline | NEA | P | -0.118 | 0.004 | 238.674 |
| 605 | Khaleghi et al., 2019 | 35.8 | 50.9 | Maclura pomifera (Raf.) | 1 | 0.5 | Proline | NEA | P | -0.109 | 0.005 | 188.060 |
| 606 | Khaleghi et al., 2019 | 35.8 | 50.9 | Maclura pomifera (Raf.) | 8 | 0.5 | Proline | NEA | P | -0.247 | 0.006 | 155.070 |
| 607 | Khaleghi et al., 2019 | 35.8 | 50.9 | Maclura pomifera (Raf.) | 15 | 0.5 | Proline | NEA | P | -0.377 | 0.007 | 138.870 |
| 608 | Khaleghi et al., 2019 | 35.8 | 50.9 | Maclura pomifera (Raf.) | 22 | 0.5 | Proline | NEA | P | -0.365 | 0.008 | 128.598 |
| 609 | Khaleghi et al., 2019 | 35.8 | 50.9 | Maclura pomifera (Raf.) | 1 | 0.7 | Proline | NEA | P | -0.109 | 0.006 | 172.101 |
| 610 | Khaleghi et al., 2019 | 35.8 | 50.9 | Maclura pomifera (Raf.) | 8 | 0.7 | Proline | NEA | P | -0.247 | 0.005 | 183.485 |
| 611 | Khaleghi et al., 2019 | 35.8 | 50.9 | Maclura pomifera (Raf.) | 15 | 0.7 | Proline | NEA | P | -0.398 | 0.005 | 219.052 |
| 612 | Khaleghi et al., 2019 | 35.8 | 50.9 | Maclura pomifera (Raf.) | 22 | 0.7 | Proline | NEA | P | -0.405 | 0.006 | 168.585 |
| 613 | Khaleghi et al., 2019 | 35.8 | 50.9 | Maclura pomifera (Raf.) | 1 | 0.25 | SOD | EA | P | 0.610 | 0.012 | 83.884 |
| 614 | Khaleghi et al., 2019 | 35.8 | 50.9 | Maclura pomifera (Raf.) | 8 | 0.25 | SOD | EA | P | 0.722 | 0.014 | 72.201 |
| 615 | Khaleghi et al., 2019 | 35.8 | 50.9 | Maclura pomifera (Raf.) | 15 | 0.25 | SOD | EA | P | 1.002 | 0.018 | 55.412 |
| 616 | Khaleghi et al., 2019 | 35.8 | 50.9 | Maclura pomifera (Raf.) | 22 | 0.25 | SOD | EA | P | 0.790 | 0.011 | 89.351 |
| 617 | Khaleghi et al., 2019 | 35.8 | 50.9 | Maclura pomifera (Raf.) | 1 | 0.5 | SOD | EA | P | 0.916 | 0.011 | 87.597 |
| 618 | Khaleghi et al., 2019 | 35.8 | 50.9 | Maclura pomifera (Raf.) | 8 | 0.5 | SOD | EA | P | 0.928 | 0.014 | 72.553 |
| 619 | Khaleghi et al., 2019 | 35.8 | 50.9 | Maclura pomifera (Raf.) | 15 | 0.5 | SOD | EA | P | 1.253 | 0.008 | 124.319 |
| 620 | Khaleghi et al., 2019 | 35.8 | 50.9 | Maclura pomifera (Raf.) | 22 | 0.5 | SOD | EA | P | 0.946 | 0.004 | 231.556 |
| 621 | Khaleghi et al., 2019 | 35.8 | 50.9 | Maclura pomifera (Raf.) | 1 | 0.7 | SOD | EA | P | 1.314 | 0.004 | 252.114 |
| 622 | Khaleghi et al., 2019 | 35.8 | 50.9 | Maclura pomifera (Raf.) | 8 | 0.7 | SOD | EA | P | 1.326 | 0.007 | 137.988 |
| 623 | Khaleghi et al., 2019 | 35.8 | 50.9 | Maclura pomifera (Raf.) | 15 | 0.7 | SOD | EA | P | 1.531 | 0.008 | 122.798 |
| 624 | Khaleghi et al., 2019 | 35.8 | 50.9 | Maclura pomifera (Raf.) | 22 | 0.7 | SOD | EA | P | 1.148 | 0.004 | 230.733 |
| 625 | Khaleghi et al., 2019 | 35.8 | 50.9 | Maclura pomifera (Raf.) | 1 | 0.25 | APX | EA | P | 0.448 | 0.005 | 202.561 |
| 626 | Khaleghi et al., 2019 | 35.8 | 50.9 | Maclura pomifera (Raf.) | 8 | 0.25 | APX | EA | P | 0.569 | 0.005 | 216.190 |
| 627 | Khaleghi et al., 2019 | 35.8 | 50.9 | Maclura pomifera (Raf.) | 15 | 0.25 | APX | EA | P | 0.567 | 0.007 | 153.719 |
| 628 | Khaleghi et al., 2019 | 35.8 | 50.9 | Maclura pomifera (Raf.) | 22 | 0.25 | APX | EA | P | 0.716 | 0.007 | 143.018 |
| 629 | Khaleghi et al., 2019 | 35.8 | 50.9 | Maclura pomifera (Raf.) | 1 | 0.5 | APX | EA | P | 1.127 | 0.006 | 181.465 |
| 630 | Khaleghi et al., 2019 | 35.8 | 50.9 | Maclura pomifera (Raf.) | 8 | 0.5 | APX | EA | P | 1.216 | 0.006 | 168.583 |
| 631 | Khaleghi et al., 2019 | 35.8 | 50.9 | Maclura pomifera (Raf.) | 15 | 0.5 | APX | EA | P | 1.316 | 0.006 | 173.226 |
| 632 | Khaleghi et al., 2019 | 35.8 | 50.9 | Maclura pomifera (Raf.) | 22 | 0.5 | APX | EA | P | 1.474 | 0.004 | 273.414 |
| 633 | Khaleghi et al., 2019 | 35.8 | 50.9 | Maclura pomifera (Raf.) | 1 | 0.7 | APX | EA | P | 1.304 | 0.005 | 217.760 |
| 634 | Khaleghi et al., 2019 | 35.8 | 50.9 | Maclura pomifera (Raf.) | 8 | 0.7 | APX | EA | P | 1.360 | 0.005 | 211.693 |
| 635 | Khaleghi et al., 2019 | 35.8 | 50.9 | Maclura pomifera (Raf.) | 15 | 0.7 | APX | EA | P | 1.516 | 0.005 | 221.718 |
| 636 | Khaleghi et al., 2019 | 35.8 | 50.9 | Maclura pomifera (Raf.) | 22 | 0.7 | APX | EA | P | 1.865 | 0.004 | 254.095 |
| 637 | Khaleghi et al., 2019 | 35.8 | 50.9 | Maclura pomifera (Raf.) | 1 | 0.25 | GR | EA | P | 0.665 | 0.009 | 110.549 |
| 638 | Khaleghi et al., 2019 | 35.8 | 50.9 | Maclura pomifera (Raf.) | 8 | 0.25 | GR | EA | P | 0.756 | 0.007 | 134.232 |
| 639 | Khaleghi et al., 2019 | 35.8 | 50.9 | Maclura pomifera (Raf.) | 15 | 0.25 | GR | EA | P | 0.784 | 0.009 | 105.525 |
| 640 | Khaleghi et al., 2019 | 35.8 | 50.9 | Maclura pomifera (Raf.) | 22 | 0.25 | GR | EA | P | 0.575 | 0.010 | 100.071 |
| 641 | Khaleghi et al., 2019 | 35.8 | 50.9 | Maclura pomifera (Raf.) | 1 | 0.5 | GR | EA | P | 0.780 | 0.008 | 120.055 |
| 642 | Khaleghi et al., 2019 | 35.8 | 50.9 | Maclura pomifera (Raf.) | 8 | 0.5 | GR | EA | P | 0.887 | 0.008 | 121.799 |
| 643 | Khaleghi et al., 2019 | 35.8 | 50.9 | Maclura pomifera (Raf.) | 15 | 0.5 | GR | EA | P | 1.003 | 0.008 | 130.923 |
| 644 | Khaleghi et al., 2019 | 35.8 | 50.9 | Maclura pomifera (Raf.) | 22 | 0.5 | GR | EA | P | 0.882 | 0.007 | 139.754 |
| 645 | Khaleghi et al., 2019 | 35.8 | 50.9 | Maclura pomifera (Raf.) | 1 | 0.7 | GR | EA | P | 0.915 | 0.008 | 122.157 |
| 646 | Khaleghi et al., 2019 | 35.8 | 50.9 | Maclura pomifera (Raf.) | 8 | 0.7 | GR | EA | P | 0.976 | 0.009 | 117.269 |
| 647 | Khaleghi et al., 2019 | 35.8 | 50.9 | Maclura pomifera (Raf.) | 15 | 0.7 | GR | EA | P | 1.245 | 0.008 | 133.269 |
| 648 | Khaleghi et al., 2019 | 35.8 | 50.9 | Maclura pomifera (Raf.) | 22 | 0.7 | GR | EA | P | 1.213 | 0.007 | 142.159 |
| 649 | Kiran et al., 2019 | 39.9 | 33 | Lactuca sativa var. crispa | 46 | 0.5 | Dry weight | Growth | S | -0.052 | 0.001 | 787.822 |
| 650 | Kiran et al., 2019 | 39.9 | 33 | Lactuca sativa var. crispa | 46 | 0.75 | Dry weight | Growth | S | -0.034 | 0.001 | 776.383 |
| 651 | Kiran et al., 2019 | 39.9 | 33 | Lactuca sativa var. crispa | 46 | 0.5 | Chl | PS | L | -0.116 | 0.006 | 174.808 |
| 652 | Kiran et al., 2019 | 39.9 | 33 | Lactuca sativa var. crispa | 46 | 0.75 | Chl | PS | L | -0.288 | 0.007 | 143.453 |
| 653 | Kiran et al., 2019 | 39.9 | 33 | Lactuca sativa var. crispa | 46 | 0.5 | EL | PMP | L | 0.205 | 0.004 | 226.271 |
| 654 | Kiran et al., 2019 | 39.9 | 33 | Lactuca sativa var. crispa | 46 | 0.75 | EL | PMP | L | 0.449 | 0.004 | 226.271 |
| 655 | Kiran et al., 2019 | 39.9 | 33 | Lactuca sativa var. crispa | 46 | 0.5 | Car | NEA | L | -0.284 | 0.015 | 68.162 |
| 656 | Kiran et al., 2019 | 39.9 | 33 | Lactuca sativa var. crispa | 46 | 0.75 | Car | NEA | L | -0.397 | 0.018 | 56.254 |
| 657 | Kiran et al., 2019 | 39.9 | 33 | Lactuca sativa var. crispa | 46 | 0.5 | MDA | PMP | L | 0.582 | 0.062 | 16.063 |
| 658 | Kiran et al., 2019 | 39.9 | 33 | Lactuca sativa var. crispa | 46 | 0.75 | MDA | PMP | L | 0.704 | 0.060 | 16.775 |
| 659 | Kiran et al., 2019 | 39.9 | 33 | Lactuca sativa var. crispa | 46 | 0.5 | SOD | EA | L | 0.671 | 0.067 | 14.932 |
| 660 | Kiran et al., 2019 | 39.9 | 33 | Lactuca sativa var. crispa | 46 | 0.75 | SOD | EA | L | 0.766 | 0.063 | 15.908 |
| 661 | Kiran et al., 2019 | 39.9 | 33 | Lactuca sativa var. crispa | 46 | 0.5 | CAT | EA | L | 1.630 | 0.405 | 2.467 |
| 662 | Kiran et al., 2019 | 39.9 | 33 | Lactuca sativa var. crispa | 46 | 0.75 | CAT | EA | L | 1.539 | 0.412 | 2.425 |
| 663 | Klunklin et al., 2017 | -43.6 | 172.4 | Solanum lycopersicum L. | 10 | 0.6 | Dry weight | Growth | P | -0.066 | 0.020 | 50.724 |
| 664 | Klunklin et al., 2017 | -43.6 | 172.4 | Solanum lycopersicum L. | 10 | 0.6 | Dry weight | Growth | P | 0.226 | 0.013 | 76.692 |
| 665 | Klunklin et al., 2017 | -43.6 | 172.4 | Solanum lycopersicum L. | 10 | 0.6 | Dry weight | Growth | P | 0.056 | 0.005 | 203.714 |
| 666 | Klunklin et al., 2017 | -43.6 | 172.4 | Solanum lycopersicum L. | 10 | 0.6 | Dry weight | Growth | P | -0.033 | 0.009 | 111.066 |
| 667 | Li et al., 2010 | 33.1 | 108.9 | Xanthoceras sorbifolia | 20 | 0.33 | MDA | PMP | L | 0.854 | 0.000 | 4474.013 |
| 668 | Li et al., 2010 | 33.1 | 108.9 | Xanthoceras sorbifolia | 20 | 0.53 | MDA | PMP | L | 1.724 | 0.000 | 5153.414 |
| 669 | Li et al., 2010 | 33.1 | 108.9 | Xanthoceras sorbifolia | 20 | 0.33 | EL | PMP | L | 0.738 | 0.002 | 618.675 |
| 670 | Li et al., 2010 | 33.1 | 108.9 | Xanthoceras sorbifolia | 20 | 0.53 | EL | PMP | L | 1.110 | 0.001 | 686.664 |
| 671 | Li et al., 2010 | 33.1 | 108.9 | Xanthoceras sorbifolia | 20 | 0.33 | Proline | NEA | L | 0.095 | 0.000 | 2495.375 |
| 672 | Li et al., 2010 | 33.1 | 108.9 | Xanthoceras sorbifolia | 20 | 0.53 | Proline | NEA | L | 0.454 | 0.002 | 425.950 |
| 673 | Li et al., 2010 | 33.1 | 108.9 | Xanthoceras sorbifolia | 20 | 0.33 | Protein | NEA | L | -0.088 | 0.001 | 1372.944 |
| 674 | Li et al., 2010 | 33.1 | 108.9 | Xanthoceras sorbifolia | 20 | 0.53 | Protein | NEA | L | -0.142 | 0.001 | 1235.181 |
| 675 | Li et al., 2010 | 33.1 | 108.9 | Xanthoceras sorbifolia | 20 | 0.33 | Soluble sugar | NEA | L | 0.776 | 0.002 | 515.193 |
| 676 | Li et al., 2010 | 33.1 | 108.9 | Xanthoceras sorbifolia | 20 | 0.53 | Soluble sugar | NEA | L | 2.208 | 0.002 | 597.705 |
| 677 | Li et al., 2010 | 33.1 | 108.9 | Xanthoceras sorbifolia | 20 | 0.33 | SOD | EA | L | -0.176 | 0.001 | 1864.697 |
| 678 | Li et al., 2010 | 33.1 | 108.9 | Xanthoceras sorbifolia | 20 | 0.53 | SOD | EA | L | -0.052 | 0.000 | 2178.553 |
| 679 | Li et al., 2010 | 33.1 | 108.9 | Xanthoceras sorbifolia | 20 | 0.33 | CAT | EA | L | 1.964 | 0.010 | 102.174 |
| 680 | Li et al., 2010 | 33.1 | 108.9 | Xanthoceras sorbifolia | 20 | 0.53 | CAT | EA | L | 1.547 | 0.021 | 48.052 |
| 681 | Li et al., 2010 | 33.1 | 108.9 | Xanthoceras sorbifolia | 20 | 0.33 | POD | EA | L | 0.332 | 0.001 | 711.596 |
| 682 | Li et al., 2010 | 33.1 | 108.9 | Xanthoceras sorbifolia | 20 | 0.53 | POD | EA | L | -0.400 | 0.006 | 157.675 |
| 683 | Li et al., 2010 | 33.1 | 108.9 | Xanthoceras sorbifolia | 20 | 0.33 | APX | EA | L | 0.203 | 0.000 | 2281.633 |
| 684 | Li et al., 2010 | 33.1 | 108.9 | Xanthoceras sorbifolia | 20 | 0.53 | APX | EA | L | -0.058 | 0.001 | 1855.552 |
| 685 | Li et al., 2010 | 33.1 | 108.9 | Xanthoceras sorbifolia | 20 | 0.33 | AsA | EA | L | -0.015 | 0.000 | 27210.633 |
| 686 | Li et al., 2010 | 33.1 | 108.9 | Xanthoceras sorbifolia | 20 | 0.53 | AsA | EA | L | -0.029 | 0.000 | 21261.966 |
| 687 | Li et al., 2010 | 33.1 | 108.9 | Xanthoceras sorbifolia | 20 | 0.33 | GR | EA | L | 0.126 | 0.008 | 121.940 |
| 688 | Li et al., 2010 | 33.1 | 108.9 | Xanthoceras sorbifolia | 20 | 0.53 | GR | EA | L | -0.419 | 0.005 | 190.614 |
| 689 | Li et al., 2011 | 41.8 | 123.5 | Oryza sativa L. | 6 | 0.5 | Chl | PS | L | -0.839 | 0.002 | 646.963 |
| 690 | Li et al., 2011 | 41.8 | 123.5 | Oryza sativa L. | 6 | 0.5 | Chl | PS | L | -1.179 | 0.006 | 171.784 |
| 691 | Li et al., 2011 | 41.8 | 123.5 | Oryza sativa L. | 6 | 0.5 | Car | NEA | L | -0.061 | 0.000 | 4525.015 |
| 692 | Li et al., 2018 | 19.3 | 109.8 | Areca catechu L. | 56 | 0.466667 | Dry weight | Growth | L | -0.380 | 0.000 | 2285.162 |
| 693 | Li et al., 2018 | 19.3 | 109.8 | Areca catechu L. | 56 | 0.73 | Dry weight | Growth | L | -0.497 | 0.000 | 2050.972 |
| 694 | Li et al., 2018 | 19.3 | 109.8 | Areca catechu L. | 56 | 0.47 | Dry weight | Growth | R | -0.228 | 0.000 | 2164.209 |
| 695 | Li et al., 2018 | 19.3 | 109.8 | Areca catechu L. | 56 | 0.733333 | Dry weight | Growth | R | -0.371 | 0.002 | 458.411 |
| 696 | Li et al., 2018 | 19.3 | 109.8 | Areca catechu L. | 56 | 0.466667 | SOD | EA | L | 0.003 | 0.000 | 2448.004 |
| 697 | Li et al., 2018 | 19.3 | 109.8 | Areca catechu L. | 56 | 0.73 | SOD | EA | L | -0.120 | 0.001 | 1254.276 |
| 698 | Li et al., 2018 | 19.3 | 109.8 | Areca catechu L. | 56 | 0.47 | POD | EA | L | 0.133 | 0.000 | 10816.180 |
| 699 | Li et al., 2018 | 19.3 | 109.8 | Areca catechu L. | 56 | 0.733333 | POD | EA | L | -0.091 | 0.000 | 5322.413 |
| 700 | Li et al., 2018 | 19.3 | 109.8 | Areca catechu L. | 56 | 0.466667 | CAT | EA | L | 0.388 | 0.009 | 115.186 |
| 701 | Li et al., 2018 | 19.3 | 109.8 | Areca catechu L. | 56 | 0.73 | CAT | EA | L | 0.703 | 0.005 | 219.630 |
| 702 | Li et al., 2018 | 19.3 | 109.8 | Areca catechu L. | 56 | 0.47 | MDA | PMP | L | 0.147 | 0.000 | 360467.814 |
| 703 | Li et al., 2018 | 19.3 | 109.8 | Areca catechu L. | 56 | 0.733333 | MDA | PMP | L | 0.282 | 0.001 | 1282.799 |
| 704 | Li et al., 2018 | 19.3 | 109.8 | Areca catechu L. | 56 | 0.466667 | Chl | PS | L | -0.445 | 0.002 | 599.001 |
| 705 | Li et al., 2018 | 19.3 | 109.8 | Areca catechu L. | 56 | 0.73 | Chl | PS | L | -0.467 | 0.004 | 242.605 |
| 706 | Li et al., 2018 | 19.3 | 109.8 | Areca catechu L. | 56 | 0.47 | Chl | PS | L | -0.452 | 0.008 | 132.794 |
| 707 | Li et al., 2018 | 19.3 | 109.8 | Areca catechu L. | 56 | 0.733333 | Chl | PS | L | -0.481 | 0.008 | 126.883 |
| 708 | Li et al., 2018 | 19.3 | 109.8 | Areca catechu L. | 56 | 0.47 | Car | NEA | L | -0.316 | 0.002 | 550.476 |
| 709 | Li et al., 2018 | 19.3 | 109.8 | Areca catechu L. | 56 | 0.733333 | Car | NEA | L | -0.470 | 0.004 | 256.228 |
| 710 | Liang et al., 2019 | 30.7 | 103.8 | Actinidia Chinensis var. deliciosa cv. Qinmei | 9 | 0.75 | Dry weight | Growth | S | -0.176 | 0.000 | 3108.649 |
| 711 | Liang et al., 2019 | 30.7 | 103.8 | Actinidia Chinensis var. deliciosa cv. Qinmei | 9 | 0.75 | Dry weight | Growth | S | -0.372 | 0.002 | 425.577 |
| 712 | Liang et al., 2019 | 30.7 | 103.8 | Actinidia Chinensis var. deliciosa cv. Qinmei | 9 | 0.75 | Dry weight | Growth | L | -0.453 | 0.000 | 2927.533 |
| 713 | Liang et al., 2019 | 30.7 | 103.8 | Actinidia Chinensis var. deliciosa cv. Qinmei | 9 | 0.75 | MDA | PMP | L | 0.515 | 0.000 | 2626.546 |
| 714 | Liang et al., 2019 | 30.7 | 103.8 | Actinidia Chinensis var. deliciosa cv. Qinmei | 9 | 0.75 | Proline | NEA | L | 0.231 | 0.011 | 93.327 |
| 715 | Liang et al., 2019 | 30.7 | 103.8 | Actinidia Chinensis var. deliciosa cv. Qinmei | 9 | 0.75 | Protein | NEA | L | -0.301 | 0.005 | 192.644 |
| 716 | Liang et al., 2019 | 30.7 | 103.8 | Actinidia Chinensis var. deliciosa cv. Qinmei | 9 | 0.75 | Chl | PS | L | -0.556 | 0.000 | 12422.188 |
| 717 | Liang et al., 2019 | 30.7 | 103.8 | Actinidia Chinensis var. deliciosa cv. Qinmei | 9 | 0.75 | Chl | PS | L | -0.663 | 0.000 | 7077.021 |
| 718 | Liang et al., 2019 | 30.7 | 103.8 | Actinidia Chinensis var. deliciosa cv. Qinmei | 9 | 0.75 | Car | PS | L | -0.526 | 0.000 | 7098.293 |
| 719 | Liang et al., 2019 | 30.7 | 103.8 | Actinidia Chinensis var. deliciosa cv. Qinmei | 9 | 0.75 | Fv/Fm | PS | L | -0.115 | 0.000 | 16285.944 |
| 720 | Liang et al., 2019 | 30.7 | 103.8 | Actinidia Chinensis var. deliciosa cv. Qinmei | 9 | 0.75 | qP | PS | L | -0.518 | 0.000 | 80884.904 |
| 721 | Lin et al., 2019 | 30.4 | 119.8 | Torreya grandi | 120 | 0.6 | Dry weight | Growth | P | -0.259 | 0.001 | 741.701 |
| 722 | Lin et al., 2019 | 30.4 | 119.8 | Torreya grandi | 120 | 0.6 | Fv/Fm | PS | L | -0.373 | 0.001 | 897.517 |
| 723 | Lin et al., 2019 | 30.4 | 119.8 | Torreya grandi | 120 | 0.6 | Proline | NEA | L | 0.224 | 0.005 | 187.596 |
| 724 | Lin et al., 2019 | 30.4 | 119.8 | Torreya grandi | 120 | 0.6 | Protein | Growth | L | 0.268 | 0.005 | 182.233 |
| 725 | Lin et al., 2019 | 30.4 | 119.8 | Torreya grandi | 120 | 0.6 | ROS | PMP | L | 0.333 | 0.002 | 492.558 |
| 726 | Lin et al., 2019 | 30.4 | 119.8 | Torreya grandi | 120 | 0.6 | ROS | PMP | L | 0.189 | 0.005 | 217.996 |
| 727 | Lin et al., 2019 | 30.4 | 119.8 | Torreya grandi | 120 | 0.6 | SOD | EA | L | 0.171 | 0.002 | 488.007 |
| 728 | Lin et al., 2019 | 30.4 | 119.8 | Torreya grandi | 120 | 0.6 | POD | EA | L | 0.100 | 0.000 | 3573.242 |
| 729 | Lin et al., 2019 | 30.4 | 119.8 | Torreya grandi | 120 | 0.6 | CAT | EA | L | 0.161 | 0.004 | 273.171 |
| 730 | Lqbal et al., 2018 | 43.46 | 87.36 | Chenopodium quinoa Willd. | 60 | 0.4 | Dry weight | Growth | S | -0.405 | 0.017 | 60.582 |
| 731 | Lqbal et al., 2018 | 43.46 | 87.36 | Chenopodium quinoa Willd. | 60 | 0.4 | ABA | NEA | L | 0.856 | 0.005 | 221.652 |
| 732 | Lqbal et al., 2018 | 43.46 | 87.36 | Chenopodium quinoa Willd. | 60 | 0.4 | Chl | PS | L | -0.584 | 0.005 | 203.948 |
| 733 | Lqbal et al., 2018 | 43.46 | 87.36 | Chenopodium quinoa Willd. | 60 | 0.4 | ROS | PMP | L | 0.669 | 0.003 | 349.350 |
| 734 | Lqbal et al., 2018 | 43.46 | 87.36 | Chenopodium quinoa Willd. | 60 | 0.4 | ROS | PMP | L | 1.247 | 0.004 | 242.220 |
| 735 | Lqbal et al., 2018 | 43.46 | 87.36 | Chenopodium quinoa Willd. | 60 | 0.4 | MDA | PMP | L | 1.020 | 0.024 | 41.052 |
| 736 | Lqbal et al., 2018 | 43.46 | 87.36 | Chenopodium quinoa Willd. | 60 | 0.4 | SOD | EA | L | 0.334 | 0.014 | 73.149 |
| 737 | Lqbal et al., 2018 | 43.46 | 87.36 | Chenopodium quinoa Willd. | 60 | 0.4 | POD | EA | L | -0.431 | 0.010 | 101.730 |
| 738 | Lqbal et al., 2018 | 43.46 | 87.36 | Chenopodium quinoa Willd. | 60 | 0.4 | CAT | EA | L | -0.556 | 0.001 | 1127.360 |
| 739 | Lqbal et al., 2018 | 43.46 | 87.36 | Chenopodium quinoa Willd. | 60 | 0.4 | APX | EA | L | 0.615 | 0.007 | 137.579 |
| 740 | Macar et al., 2009 | 39.9 | 32.9 | Cicer arietinum L. | 3 | 0.42 | Dry weight | Growth | S | -0.382 | 0.003 | 318.563 |
| 741 | Macar et al., 2009 | 39.9 | 32.9 | Cicer arietinum L. | 5 | 0.53 | Dry weight | Growth | S | -0.872 | 0.048 | 20.824 |
| 742 | Macar et al., 2009 | 39.9 | 32.9 | Cicer arietinum L. | 7 | 0.67 | Dry weight | Growth | S | -1.279 | 0.009 | 107.018 |
| 743 | Macar et al., 2009 | 39.9 | 32.9 | Cicer arietinum L. | 3 | 0.42 | Dry weight | Growth | S | -0.463 | 0.004 | 258.155 |
| 744 | Macar et al., 2009 | 39.9 | 32.9 | Cicer arietinum L. | 5 | 0.53 | Dry weight | Growth | S | -1.057 | 0.017 | 60.453 |
| 745 | Macar et al., 2009 | 39.9 | 32.9 | Cicer arietinum L. | 7 | 0.67 | Dry weight | Growth | S | -1.401 | 0.012 | 83.573 |
| 746 | Macar et al., 2009 | 39.9 | 32.9 | Cicer arietinum L. | 3 | 0.42 | Fv/Fm | PS | L | 0.000 | 0.003 | 339.406 |
| 747 | Macar et al., 2009 | 39.9 | 32.9 | Cicer arietinum L. | 5 | 0.53 | Fv/Fm | PS | L | -0.074 | 0.003 | 339.406 |
| 748 | Macar et al., 2009 | 39.9 | 32.9 | Cicer arietinum L. | 7 | 0.67 | Fv/Fm | PS | L | -0.076 | 0.002 | 497.372 |
| 749 | Macar et al., 2009 | 39.9 | 32.9 | Cicer arietinum L. | 3 | 0.42 | Fv/Fm | PS | L | -0.036 | 0.003 | 339.406 |
| 750 | Macar et al., 2009 | 39.9 | 32.9 | Cicer arietinum L. | 5 | 0.53 | Fv/Fm | PS | L | -0.099 | 0.003 | 339.406 |
| 751 | Macar et al., 2009 | 39.9 | 32.9 | Cicer arietinum L. | 7 | 0.67 | Fv/Fm | PS | L | -0.311 | 0.006 | 168.065 |
| 752 | Macar et al., 2009 | 39.9 | 32.9 | Cicer arietinum L. | 3 | 0.42 | qP | PS | L | -0.041 | 0.003 | 339.406 |
| 753 | Macar et al., 2009 | 39.9 | 32.9 | Cicer arietinum L. | 5 | 0.53 | qP | PS | L | -0.119 | 0.003 | 339.406 |
| 754 | Macar et al., 2009 | 39.9 | 32.9 | Cicer arietinum L. | 7 | 0.67 | qP | PS | L | -0.147 | 0.004 | 266.056 |
| 755 | Macar et al., 2009 | 39.9 | 32.9 | Cicer arietinum L. | 3 | 0.42 | qP | PS | L | -0.073 | 0.003 | 339.406 |
| 756 | Macar et al., 2009 | 39.9 | 32.9 | Cicer arietinum L. | 5 | 0.53 | qP | PS | L | -0.131 | 0.003 | 339.406 |
| 757 | Macar et al., 2009 | 39.9 | 32.9 | Cicer arietinum L. | 7 | 0.67 | qP | PS | L | -0.305 | 0.006 | 180.662 |
| 758 | Macar et al., 2009 | 39.9 | 32.9 | Cicer arietinum L. | 3 | 0.42 | Chl | PS | L | -0.126 | 0.003 | 327.318 |
| 759 | Macar et al., 2009 | 39.9 | 32.9 | Cicer arietinum L. | 5 | 0.53 | Chl | PS | L | -0.249 | 0.003 | 390.333 |
| 760 | Macar et al., 2009 | 39.9 | 32.9 | Cicer arietinum L. | 7 | 0.67 | Chl | PS | L | -0.285 | 0.002 | 443.013 |
| 761 | Macar et al., 2009 | 39.9 | 32.9 | Cicer arietinum L. | 3 | 0.42 | Car | NEA | L | -0.037 | 0.013 | 74.428 |
| 762 | Macar et al., 2009 | 39.9 | 32.9 | Cicer arietinum L. | 5 | 0.53 | Car | NEA | L | 0.033 | 0.017 | 59.742 |
| 763 | Macar et al., 2009 | 39.9 | 32.9 | Cicer arietinum L. | 7 | 0.67 | Car | NEA | L | 0.029 | 0.013 | 78.288 |
| 764 | Macar et al., 2009 | 39.9 | 32.9 | Cicer arietinum L. | 3 | 0.42 | MDA | PMP | L | 0.799 | 0.005 | 208.373 |
| 765 | Macar et al., 2009 | 39.9 | 32.9 | Cicer arietinum L. | 5 | 0.53 | MDA | PMP | L | 0.924 | 0.005 | 199.208 |
| 766 | Macar et al., 2009 | 39.9 | 32.9 | Cicer arietinum L. | 7 | 0.67 | MDA | PMP | L | 1.051 | 0.002 | 577.404 |
| 767 | Macar et al., 2009 | 39.9 | 32.9 | Cicer arietinum L. | 3 | 0.42 | Proline | NEA | L | 2.032 | 0.006 | 169.759 |
| 768 | Macar et al., 2009 | 39.9 | 32.9 | Cicer arietinum L. | 5 | 0.53 | Proline | NEA | L | 2.179 | 0.005 | 188.967 |
| 769 | Macar et al., 2009 | 39.9 | 32.9 | Cicer arietinum L. | 7 | 0.67 | Proline | NEA | L | 2.845 | 0.009 | 107.516 |
| 770 | Macar et al., 2009 | 39.9 | 32.9 | Cicer arietinum L. | 3 | 0.42 | SOD | EA | L | 0.092 | 0.063 | 15.987 |
| 771 | Macar et al., 2009 | 39.9 | 32.9 | Cicer arietinum L. | 5 | 0.53 | SOD | EA | L | 1.485 | 0.047 | 21.449 |
| 772 | Macar et al., 2009 | 39.9 | 32.9 | Cicer arietinum L. | 7 | 0.67 | SOD | EA | L | 0.327 | 0.129 | 7.754 |
| 773 | Macar et al., 2009 | 39.9 | 32.9 | Cicer arietinum L. | 3 | 0.42 | SOD | EA | L | 0.671 | 0.009 | 114.822 |
| 774 | Macar et al., 2009 | 39.9 | 32.9 | Cicer arietinum L. | 5 | 0.53 | SOD | EA | L | -0.248 | 0.012 | 84.023 |
| 775 | Macar et al., 2009 | 39.9 | 32.9 | Cicer arietinum L. | 7 | 0.67 | SOD | EA | L | 0.621 | 0.003 | 322.352 |
| 776 | Macar et al., 2009 | 39.9 | 32.9 | Cicer arietinum L. | 3 | 0.42 | APX | EA | L | -0.197 | 0.007 | 134.811 |
| 777 | Macar et al., 2009 | 39.9 | 32.9 | Cicer arietinum L. | 5 | 0.53 | APX | EA | L | 0.303 | 0.013 | 79.131 |
| 778 | Macar et al., 2009 | 39.9 | 32.9 | Cicer arietinum L. | 7 | 0.67 | APX | EA | L | 0.155 | 0.003 | 393.250 |
| 779 | Macar et al., 2009 | 39.9 | 32.9 | Cicer arietinum L. | 3 | 0.42 | APX | EA | L | 0.448 | 0.005 | 208.028 |
| 780 | Macar et al., 2009 | 39.9 | 32.9 | Cicer arietinum L. | 5 | 0.53 | APX | EA | L | -0.318 | 0.008 | 128.834 |
| 781 | Macar et al., 2009 | 39.9 | 32.9 | Cicer arietinum L. | 7 | 0.67 | APX | EA | L | 0.474 | 0.006 | 162.561 |
| 782 | Macar et al., 2009 | 39.9 | 32.9 | Cicer arietinum L. | 3 | 0.42 | GR | EA | L | 0.519 | 0.007 | 150.241 |
| 783 | Macar et al., 2009 | 39.9 | 32.9 | Cicer arietinum L. | 5 | 0.53 | GR | EA | L | 0.573 | 0.005 | 207.019 |
| 784 | Macar et al., 2009 | 39.9 | 32.9 | Cicer arietinum L. | 7 | 0.67 | GR | EA | L | 0.691 | 0.020 | 50.614 |
| 785 | Macar et al., 2009 | 39.9 | 32.9 | Cicer arietinum L. | 3 | 0.42 | GR | EA | L | 0.527 | 0.005 | 217.237 |
| 786 | Macar et al., 2009 | 39.9 | 32.9 | Cicer arietinum L. | 5 | 0.53 | GR | EA | L | 0.358 | 0.006 | 163.776 |
| 787 | Macar et al., 2009 | 39.9 | 32.9 | Cicer arietinum L. | 7 | 0.67 | GR | EA | L | 0.415 | 0.021 | 47.708 |
| 788 | Macar et al., 2009 | 39.9 | 32.9 | Cicer arietinum L. | 3 | 0.42 | POD | EA | L | 0.399 | 0.001 | 1151.955 |
| 789 | Macar et al., 2009 | 39.9 | 32.9 | Cicer arietinum L. | 5 | 0.53 | POD | EA | L | 0.380 | 0.004 | 269.254 |
| 790 | Macar et al., 2009 | 39.9 | 32.9 | Cicer arietinum L. | 7 | 0.67 | POD | EA | L | 0.777 | 0.003 | 363.713 |
| 791 | Macar et al., 2009 | 39.9 | 32.9 | Cicer arietinum L. | 3 | 0.42 | POD | EA | L | 0.414 | 0.007 | 152.001 |
| 792 | Macar et al., 2009 | 39.9 | 32.9 | Cicer arietinum L. | 5 | 0.53 | POD | EA | L | 0.333 | 0.002 | 443.939 |
| 793 | Macar et al., 2009 | 39.9 | 32.9 | Cicer arietinum L. | 7 | 0.67 | POD | EA | L | 0.517 | 0.004 | 222.280 |
| 794 | Maghsoudi et al., 2019 | 29.4 | 52.8 | Triticum aestivum L. | 30 | 0.6 | Dry weight | Growth | P | -0.623 | 0.006 | 169.703 |
| 795 | Maghsoudi et al., 2019 | 29.4 | 52.8 | Triticum aestivum L. | 30 | 0.6 | Dry weight | Growth | P | -0.307 | 0.006 | 169.703 |
| 796 | Maghsoudi et al., 2019 | 29.4 | 52.8 | Triticum aestivum L. | 30 | 0.6 | Protein | Growth | L | -0.450 | 0.006 | 169.703 |
| 797 | Maghsoudi et al., 2019 | 29.4 | 52.8 | Triticum aestivum L. | 30 | 0.6 | Protein | Growth | L | -0.226 | 0.006 | 169.703 |
| 798 | Maghsoudi et al., 2019 | 29.4 | 52.8 | Triticum aestivum L. | 30 | 0.6 | POD | EA | L | 0.053 | 0.006 | 169.703 |
| 799 | Maghsoudi et al., 2019 | 29.4 | 52.8 | Triticum aestivum L. | 30 | 0.6 | POD | EA | L | 0.560 | 0.006 | 169.703 |
| 800 | Maghsoudi et al., 2019 | 29.4 | 52.8 | Triticum aestivum L. | 30 | 0.6 | SOD | EA | L | 0.297 | 0.006 | 169.703 |
| 801 | Maghsoudi et al., 2019 | 29.4 | 52.8 | Triticum aestivum L. | 30 | 0.6 | SOD | EA | L | 0.486 | 0.006 | 169.703 |
| 802 | Maghsoudi et al., 2019 | 29.4 | 52.8 | Triticum aestivum L. | 30 | 0.6 | APX | EA | L | 0.306 | 0.006 | 169.703 |
| 803 | Maghsoudi et al., 2019 | 29.4 | 52.8 | Triticum aestivum L. | 30 | 0.6 | APX | EA | L | 0.889 | 0.006 | 169.703 |
| 804 | Maghsoudi et al., 2019 | 29.4 | 52.8 | Triticum aestivum L. | 30 | 0.6 | CAT | EA | L | 0.484 | 0.006 | 169.703 |
| 805 | Maghsoudi et al., 2019 | 29.4 | 52.8 | Triticum aestivum L. | 30 | 0.6 | CAT | EA | L | 0.838 | 0.006 | 169.703 |
| 806 | Maghsoudi et al., 2019 | 29.4 | 52.8 | Triticum aestivum L. | 30 | 0.6 | ROS | PMP | L | 1.576 | 0.006 | 169.703 |
| 807 | Maghsoudi et al., 2019 | 29.4 | 52.8 | Triticum aestivum L. | 30 | 0.6 | ROS | PMP | L | 1.336 | 0.006 | 169.703 |
| 808 | Maghsoudi et al., 2019 | 29.4 | 52.8 | Triticum aestivum L. | 30 | 0.6 | MDA | PMP | L | 1.155 | 0.006 | 169.703 |
| 809 | Maghsoudi et al., 2019 | 29.4 | 52.8 | Triticum aestivum L. | 30 | 0.6 | MDA | PMP | L | 0.802 | 0.006 | 169.703 |
| 810 | Masoumi et al., 2010 | 35.8 | 51.2 | Glycine max L. | 150 | 0.5 | SOD | EA | L | 0.379 | 0.004 | 226.271 |
| 811 | Masoumi et al., 2010 | 35.8 | 51.2 | Glycine max L. | 150 | 0.5 | SOD | EA | L | 0.262 | 0.004 | 226.271 |
| 812 | Masoumi et al., 2010 | 35.8 | 51.2 | Glycine max L. | 150 | 0.5 | SOD | EA | L | 0.351 | 0.004 | 226.271 |
| 813 | Masoumi et al., 2010 | 35.8 | 51.2 | Glycine max L. | 150 | 0.5 | SOD | EA | L | 0.609 | 0.004 | 226.271 |
| 814 | Masoumi et al., 2010 | 35.8 | 51.2 | Glycine max L. | 150 | 0.5 | SOD | EA | L | 0.527 | 0.004 | 226.271 |
| 815 | Masoumi et al., 2010 | 35.8 | 51.2 | Glycine max L. | 150 | 0.7 | SOD | EA | L | 0.114 | 0.004 | 226.271 |
| 816 | Masoumi et al., 2010 | 35.8 | 51.2 | Glycine max L. | 150 | 0.7 | SOD | EA | L | 0.212 | 0.004 | 226.271 |
| 817 | Masoumi et al., 2010 | 35.8 | 51.2 | Glycine max L. | 150 | 0.7 | SOD | EA | L | 0.140 | 0.004 | 226.271 |
| 818 | Masoumi et al., 2010 | 35.8 | 51.2 | Glycine max L. | 150 | 0.7 | SOD | EA | L | 0.528 | 0.004 | 226.271 |
| 819 | Masoumi et al., 2010 | 35.8 | 51.2 | Glycine max L. | 150 | 0.7 | SOD | EA | L | 0.471 | 0.004 | 226.271 |
| 820 | Masoumi et al., 2010 | 35.8 | 51.2 | Glycine max L. | 150 | 0.5 | CAT | EA | L | 0.120 | 0.004 | 226.271 |
| 821 | Masoumi et al., 2010 | 35.8 | 51.2 | Glycine max L. | 150 | 0.5 | CAT | EA | L | 0.113 | 0.004 | 226.271 |
| 822 | Masoumi et al., 2010 | 35.8 | 51.2 | Glycine max L. | 150 | 0.5 | CAT | EA | L | 0.350 | 0.004 | 226.271 |
| 823 | Masoumi et al., 2010 | 35.8 | 51.2 | Glycine max L. | 150 | 0.5 | CAT | EA | L | 0.272 | 0.004 | 226.271 |
| 824 | Masoumi et al., 2010 | 35.8 | 51.2 | Glycine max L. | 150 | 0.5 | CAT | EA | L | 0.275 | 0.004 | 226.271 |
| 825 | Masoumi et al., 2010 | 35.8 | 51.2 | Glycine max L. | 150 | 0.7 | CAT | EA | L | 0.037 | 0.004 | 226.271 |
| 826 | Masoumi et al., 2010 | 35.8 | 51.2 | Glycine max L. | 150 | 0.7 | CAT | EA | L | 0.072 | 0.004 | 226.271 |
| 827 | Masoumi et al., 2010 | 35.8 | 51.2 | Glycine max L. | 150 | 0.7 | CAT | EA | L | 0.140 | 0.004 | 226.271 |
| 828 | Masoumi et al., 2010 | 35.8 | 51.2 | Glycine max L. | 150 | 0.7 | CAT | EA | L | 0.238 | 0.004 | 226.271 |
| 829 | Masoumi et al., 2010 | 35.8 | 51.2 | Glycine max L. | 150 | 0.7 | CAT | EA | L | 0.274 | 0.004 | 226.271 |
| 830 | Masoumi et al., 2010 | 35.8 | 51.2 | Glycine max L. | 150 | 0.5 | Chl | PS | L | -0.212 | 0.004 | 226.271 |
| 831 | Masoumi et al., 2010 | 35.8 | 51.2 | Glycine max L. | 150 | 0.5 | Chl | PS | L | -0.213 | 0.004 | 226.271 |
| 832 | Masoumi et al., 2010 | 35.8 | 51.2 | Glycine max L. | 150 | 0.5 | Chl | PS | L | -0.196 | 0.004 | 226.271 |
| 833 | Masoumi et al., 2010 | 35.8 | 51.2 | Glycine max L. | 150 | 0.5 | Chl | PS | L | -0.092 | 0.004 | 226.271 |
| 834 | Masoumi et al., 2010 | 35.8 | 51.2 | Glycine max L. | 150 | 0.5 | Chl | PS | L | -0.151 | 0.004 | 226.271 |
| 835 | Masoumi et al., 2010 | 35.8 | 51.2 | Glycine max L. | 150 | 0.7 | Chl | PS | L | -0.462 | 0.004 | 226.271 |
| 836 | Masoumi et al., 2010 | 35.8 | 51.2 | Glycine max L. | 150 | 0.7 | Chl | PS | L | -0.411 | 0.004 | 226.271 |
| 837 | Masoumi et al., 2010 | 35.8 | 51.2 | Glycine max L. | 150 | 0.7 | Chl | PS | L | -0.622 | 0.004 | 226.271 |
| 838 | Masoumi et al., 2010 | 35.8 | 51.2 | Glycine max L. | 150 | 0.7 | Chl | PS | L | -0.179 | 0.004 | 226.271 |
| 839 | Masoumi et al., 2010 | 35.8 | 51.2 | Glycine max L. | 150 | 0.7 | Chl | PS | L | -0.287 | 0.004 | 226.271 |
| 840 | Mathur et al., 2018 | 22.7 | 75.9 | Triticum aestivum L. | 30 | 0.75 | Chl | PS | L | -0.904 | 0.003 | 314.935 |
| 841 | Mathur et al., 2018 | 22.7 | 75.9 | Triticum aestivum L. | 30 | 0.75 | Fv/Fm | PS | L | -1.447 | 0.026 | 38.918 |
| 842 | Moles et al., 2018 | 43.7 | 10.4 | tomato(Ciettaicale) | 20 | 0.5 | Dry weight | Growth | R | -0.288 | 0.001 | 1875.405 |
| 843 | Moles et al., 2018 | 43.7 | 10.4 | tomato(Moneymaker) | 20 | 0.5 | Dry weight | Growth | R | -1.323 | 0.002 | 528.191 |
| 844 | Moles et al., 2018 | 43.7 | 10.4 | tomato(Ciettaicale) | 20 | 0.5 | Fv/Fm | PS | L | 0.000 | 0.004 | 282.838 |
| 845 | Moles et al., 2018 | 43.7 | 10.4 | tomato(Moneymaker) | 20 | 0.5 | Fv/Fm | PS | L | 0.000 | 0.004 | 282.838 |
| 846 | Moles et al., 2018 | 43.7 | 10.4 | tomato(Ciettaicale) | 20 | 0.5 | ABA | NEA | L | 2.230 | 0.000 | 8719.137 |
| 847 | Moles et al., 2018 | 43.7 | 10.4 | tomato(Moneymaker) | 20 | 0.5 | ABA | NEA | L | 2.340 | 0.000 | 13757.558 |
| 848 | Moles et al., 2018 | 43.7 | 10.4 | tomato(Ciettaicale) | 20 | 0.5 | ABA | NEA | R | 2.331 | 0.023 | 42.693 |
| 849 | Moles et al., 2018 | 43.7 | 10.4 | tomato(Moneymaker) | 20 | 0.5 | ABA | NEA | R | 2.627 | 0.002 | 657.809 |
| 850 | Moles et al., 2018 | 43.7 | 10.4 | tomato(Ciettaicale) | 20 | 0.5 | ROS | PMP | L | 0.321 | 0.009 | 106.249 |
| 851 | Moles et al., 2018 | 43.7 | 10.4 | tomato(Moneymaker) | 20 | 0.5 | ROS | PMP | L | 0.604 | 0.010 | 96.997 |
| 852 | Moles et al., 2018 | 43.7 | 10.4 | tomato(Ciettaicale) | 20 | 0.5 | ROS | PMP | R | 0.509 | 0.001 | 1211.570 |
| 853 | Moles et al., 2018 | 43.7 | 10.4 | tomato(Moneymaker) | 20 | 0.5 | ROS | PMP | R | 0.558 | 0.008 | 122.255 |
| 854 | Moles et al., 2018 | 43.7 | 10.4 | tomato(Ciettaicale) | 20 | 0.5 | MDA | PMP | L | 0.280 | 0.003 | 323.015 |
| 855 | Moles et al., 2018 | 43.7 | 10.4 | tomato(Moneymaker) | 20 | 0.5 | MDA | PMP | L | 0.647 | 0.002 | 406.715 |
| 856 | Moles et al., 2018 | 43.7 | 10.4 | tomato(Ciettaicale) | 20 | 0.5 | MDA | PMP | R | 0.245 | 0.009 | 111.014 |
| 857 | Moles et al., 2018 | 43.7 | 10.4 | tomato(Moneymaker) | 20 | 0.5 | MDA | PMP | R | 0.855 | 0.011 | 93.090 |
| 858 | Moles et al., 2018 | 43.7 | 10.4 | tomato(Ciettaicale) | 20 | 0.5 | Soluble sugar | NEA | L | 0.705 | 0.003 | 355.796 |
| 859 | Moles et al., 2018 | 43.7 | 10.4 | tomato(Moneymaker) | 20 | 0.5 | Soluble sugar | NEA | L | 1.413 | 0.006 | 163.266 |
| 860 | Moles et al., 2018 | 43.7 | 10.4 | tomato(Ciettaicale) | 20 | 0.5 | Soluble sugar | NEA | R | 1.312 | 0.016 | 61.523 |
| 861 | Moles et al., 2018 | 43.7 | 10.4 | tomato(Moneymaker) | 20 | 0.5 | Soluble sugar | NEA | R | 1.824 | 0.015 | 66.325 |
| 862 | Moles et al., 2018 | 43.7 | 10.4 | tomato(Ciettaicale) | 20 | 0.5 | Proline | NEA | L | 2.275 | 0.014 | 70.590 |
| 863 | Moles et al., 2018 | 43.7 | 10.4 | tomato(Moneymaker) | 20 | 0.5 | Proline | NEA | L | 1.911 | 0.016 | 63.777 |
| 864 | Moles et al., 2018 | 43.7 | 10.4 | tomato(Ciettaicale) | 20 | 0.5 | Proline | NEA | R | 0.965 | 0.032 | 31.391 |
| 865 | Moles et al., 2018 | 43.7 | 10.4 | tomato(Moneymaker) | 20 | 0.5 | Proline | NEA | R | 1.543 | 0.025 | 40.623 |
| 866 | Nankishore et al., 2016 | 11.2 | -60.5 | Solanum lycopersicum L. | 11 | 0.8 | Chl | PS | L | -0.041 | 0.008 | 132.358 |
| 867 | Nankishore et al., 2016 | 11.2 | -60.5 | Solanum lycopersicum L. | 11 | 0.8 | Chl | PS | L | -0.062 | 0.005 | 186.841 |
| 868 | Nankishore et al., 2016 | 11.2 | -60.5 | Solanum lycopersicum L. | 11 | 0.8 | Chl | PS | L | -0.092 | 0.007 | 151.330 |
| 869 | Nankishore et al., 2016 | 11.2 | -60.5 | Solanum lycopersicum L. | 10 | 0.8 | Fv/Fm | PS | L | -0.076 | 0.000 | 6466.231 |
| 870 | Nankishore et al., 2016 | 11.2 | -60.5 | Solanum lycopersicum L. | 10 | 0.8 | Fv/Fm | PS | L | -0.119 | 0.000 | 9874.880 |
| 871 | Nankishore et al., 2016 | 11.2 | -60.5 | Solanum lycopersicum L. | 10 | 0.8 | Fv/Fm | PS | L | -0.476 | 0.000 | 2980.230 |
| 872 | Nazarli et al., 2011a | 37.5 | 45.1 | Triticum aestivum L. cv. Sardari | 60 | 0.25 | Proline | NEA | L | -0.285 | 0.006 | 169.703 |
| 873 | Nazarli et al., 2011a | 37.5 | 45.1 | Triticum aestivum L. cv. Sardari | 60 | 0.5 | Proline | NEA | L | -0.584 | 0.006 | 169.703 |
| 874 | Nazarli et al., 2011a | 37.5 | 45.1 | Triticum aestivum L. cv. Sardari | 60 | 0.75 | Proline | NEA | L | -1.648 | 0.006 | 169.703 |
| 875 | Nazarli et al., 2011a | 37.5 | 45.1 | Triticum aestivum L. cv. Sardari | 60 | 0.25 | APX | EA | L | -0.364 | 0.006 | 169.703 |
| 876 | Nazarli et al., 2011a | 37.5 | 45.1 | Triticum aestivum L. cv. Sardari | 60 | 0.5 | APX | EA | L | -0.877 | 0.006 | 169.703 |
| 877 | Nazarli et al., 2011a | 37.5 | 45.1 | Triticum aestivum L. cv. Sardari | 60 | 0.75 | APX | EA | L | -1.032 | 0.006 | 169.703 |
| 878 | Nazarli et al., 2011b | 37.5 | 45.1 | Helianthus annuus L. cv. master | 50 | 0.4 | CAT | EA | L | 0.628 | 0.092 | 10.911 |
| 879 | Nazarli et al., 2011b | 37.5 | 45.1 | Helianthus annuus L. cv. master | 50 | 0.57 | CAT | EA | L | 1.202 | 0.087 | 11.554 |
| 880 | Nazarli et al., 2011b | 37.5 | 45.1 | Helianthus annuus L. cv. master | 50 | 0.4 | APX | EA | L | 0.580 | 0.034 | 29.079 |
| 881 | Nazarli et al., 2011b | 37.5 | 45.1 | Helianthus annuus L. cv. master | 50 | 0.57 | APX | EA | L | 0.887 | 0.033 | 29.981 |
| 882 | Nikolaeva et al., 2010 | 55.7 | 37.6 | Triticum aestivum L.(Ballada) | 3 | 0.42 | Chl | PS | L | 0.102 | 0.006 | 169.703 |
| 883 | Nikolaeva et al., 2010 | 55.7 | 37.6 | Triticum aestivum L.(Ballada) | 5 | 0.53 | Chl | PS | L | -0.108 | 0.006 | 169.703 |
| 884 | Nikolaeva et al., 2010 | 55.7 | 37.6 | Triticum aestivum L.(Ballada) | 7 | 0.67 | Chl | PS | L | -0.178 | 0.006 | 169.703 |
| 885 | Nikolaeva et al., 2010 | 55.7 | 37.6 | Triticum aestivum L.(Belchanka) | 3 | 0.42 | Chl | PS | L | 0.009 | 0.006 | 169.703 |
| 886 | Nikolaeva et al., 2010 | 55.7 | 37.6 | Triticum aestivum L.(Belchanka) | 5 | 0.53 | Chl | PS | L | -0.132 | 0.006 | 169.703 |
| 887 | Nikolaeva et al., 2010 | 55.7 | 37.6 | Triticum aestivum L.(Belchanka) | 7 | 0.67 | Chl | PS | L | -0.140 | 0.006 | 169.703 |
| 888 | Nikolaeva et al., 2010 | 55.7 | 37.6 | Triticum aestivum L.(Beltskaya) | 3 | 0.42 | Chl | PS | L | 0.069 | 0.006 | 169.703 |
| 889 | Nikolaeva et al., 2010 | 55.7 | 37.6 | Triticum aestivum L.(Beltskaya) | 5 | 0.53 | Chl | PS | L | -0.083 | 0.006 | 169.703 |
| 890 | Nikolaeva et al., 2010 | 55.7 | 37.6 | Triticum aestivum L.(Beltskaya) | 7 | 0.67 | Chl | PS | L | -0.128 | 0.006 | 169.703 |
| 891 | Nikolaeva et al., 2010 | 55.7 | 37.6 | Triticum aestivum L.(Ballada) | 3 | 0.42 | Chl | PS | L | 0.095 | 0.006 | 169.703 |
| 892 | Nikolaeva et al., 2010 | 55.7 | 37.6 | Triticum aestivum L.(Ballada) | 5 | 0.53 | Chl | PS | L | -0.034 | 0.006 | 169.703 |
| 893 | Nikolaeva et al., 2010 | 55.7 | 37.6 | Triticum aestivum L.(Ballada) | 7 | 0.67 | Chl | PS | L | -0.143 | 0.006 | 169.703 |
| 894 | Nikolaeva et al., 2010 | 55.7 | 37.6 | Triticum aestivum L.(Belchanka) | 3 | 0.42 | Chl | PS | L | 0.002 | 0.006 | 169.703 |
| 895 | Nikolaeva et al., 2010 | 55.7 | 37.6 | Triticum aestivum L.(Belchanka) | 5 | 0.53 | Chl | PS | L | -0.140 | 0.006 | 169.703 |
| 896 | Nikolaeva et al., 2010 | 55.7 | 37.6 | Triticum aestivum L.(Belchanka) | 7 | 0.67 | Chl | PS | L | -0.217 | 0.006 | 169.703 |
| 897 | Nikolaeva et al., 2010 | 55.7 | 37.6 | Triticum aestivum L.(Beltskaya) | 3 | 0.42 | Chl | PS | L | 0.015 | 0.006 | 169.703 |
| 898 | Nikolaeva et al., 2010 | 55.7 | 37.6 | Triticum aestivum L.(Beltskaya) | 5 | 0.53 | Chl | PS | L | -0.040 | 0.006 | 169.703 |
| 899 | Nikolaeva et al., 2010 | 55.7 | 37.6 | Triticum aestivum L.(Beltskaya) | 7 | 0.67 | Chl | PS | L | -0.151 | 0.006 | 169.703 |
| 900 | Nikolaeva et al., 2010 | 55.7 | 37.6 | Triticum aestivum L.(Ballada) | 3 | 0.42 | GR | EA | L | 0.130 | 0.006 | 169.703 |
| 901 | Nikolaeva et al., 2010 | 55.7 | 37.6 | Triticum aestivum L.(Ballada) | 5 | 0.53 | GR | EA | L | 0.081 | 0.006 | 169.703 |
| 902 | Nikolaeva et al., 2010 | 55.7 | 37.6 | Triticum aestivum L.(Ballada) | 7 | 0.67 | GR | EA | L | -0.143 | 0.006 | 169.703 |
| 903 | Nikolaeva et al., 2010 | 55.7 | 37.6 | Triticum aestivum L.(Belchanka) | 3 | 0.42 | GR | EA | L | 0.332 | 0.006 | 169.703 |
| 904 | Nikolaeva et al., 2010 | 55.7 | 37.6 | Triticum aestivum L.(Belchanka) | 5 | 0.53 | GR | EA | L | 0.311 | 0.006 | 169.703 |
| 905 | Nikolaeva et al., 2010 | 55.7 | 37.6 | Triticum aestivum L.(Belchanka) | 7 | 0.67 | GR | EA | L | -0.380 | 0.006 | 169.703 |
| 906 | Nikolaeva et al., 2010 | 55.7 | 37.6 | Triticum aestivum L.(Beltskaya) | 3 | 0.42 | GR | EA | L | -0.484 | 0.006 | 169.703 |
| 907 | Nikolaeva et al., 2010 | 55.7 | 37.6 | Triticum aestivum L.(Beltskaya) | 5 | 0.53 | GR | EA | L | -0.499 | 0.006 | 169.703 |
| 908 | Nikolaeva et al., 2010 | 55.7 | 37.6 | Triticum aestivum L.(Beltskaya) | 7 | 0.67 | GR | EA | L | -0.558 | 0.006 | 169.703 |
| 909 | Nikolaeva et al., 2010 | 55.7 | 37.6 | Triticum aestivum L.(Ballada) | 3 | 0.42 | APX | EA | L | 0.385 | 0.006 | 169.703 |
| 910 | Nikolaeva et al., 2010 | 55.7 | 37.6 | Triticum aestivum L.(Ballada) | 5 | 0.53 | APX | EA | L | -0.072 | 0.006 | 169.703 |
| 911 | Nikolaeva et al., 2010 | 55.7 | 37.6 | Triticum aestivum L.(Ballada) | 7 | 0.67 | APX | EA | L | -0.224 | 0.006 | 169.703 |
| 912 | Nikolaeva et al., 2010 | 55.7 | 37.6 | Triticum aestivum L.(Belchanka) | 3 | 0.42 | APX | EA | L | 0.123 | 0.006 | 169.703 |
| 913 | Nikolaeva et al., 2010 | 55.7 | 37.6 | Triticum aestivum L.(Belchanka) | 5 | 0.53 | APX | EA | L | 0.097 | 0.006 | 169.703 |
| 914 | Nikolaeva et al., 2010 | 55.7 | 37.6 | Triticum aestivum L.(Belchanka) | 7 | 0.67 | APX | EA | L | -0.273 | 0.006 | 169.703 |
| 915 | Nikolaeva et al., 2010 | 55.7 | 37.6 | Triticum aestivum L.(Beltskaya) | 3 | 0.42 | APX | EA | L | -0.122 | 0.006 | 169.703 |
| 916 | Nikolaeva et al., 2010 | 55.7 | 37.6 | Triticum aestivum L.(Beltskaya) | 5 | 0.53 | APX | EA | L | -0.203 | 0.006 | 169.703 |
| 917 | Nikolaeva et al., 2010 | 55.7 | 37.6 | Triticum aestivum L.(Beltskaya) | 7 | 0.67 | APX | EA | L | -0.306 | 0.006 | 169.703 |
| 918 | Nikolaeva et al., 2010 | 55.7 | 37.6 | Triticum aestivum L.(Ballada) | 3 | 0.42 | MDA | PMP | L | 0.176 | 0.006 | 169.703 |
| 919 | Nikolaeva et al., 2010 | 55.7 | 37.6 | Triticum aestivum L.(Ballada) | 5 | 0.53 | MDA | PMP | L | 0.177 | 0.006 | 169.703 |
| 920 | Nikolaeva et al., 2010 | 55.7 | 37.6 | Triticum aestivum L.(Ballada) | 7 | 0.67 | MDA | PMP | L | 0.463 | 0.006 | 169.703 |
| 921 | Nikolaeva et al., 2010 | 55.7 | 37.6 | Triticum aestivum L.(Belchanka) | 3 | 0.42 | MDA | PMP | L | 0.026 | 0.006 | 169.703 |
| 922 | Nikolaeva et al., 2010 | 55.7 | 37.6 | Triticum aestivum L.(Belchanka) | 5 | 0.53 | MDA | PMP | L | 0.118 | 0.006 | 169.703 |
| 923 | Nikolaeva et al., 2010 | 55.7 | 37.6 | Triticum aestivum L.(Belchanka) | 7 | 0.67 | MDA | PMP | L | 0.513 | 0.006 | 169.703 |
| 924 | Nikolaeva et al., 2010 | 55.7 | 37.6 | Triticum aestivum L.(Beltskaya) | 3 | 0.42 | MDA | PMP | L | 0.087 | 0.006 | 169.703 |
| 925 | Nikolaeva et al., 2010 | 55.7 | 37.6 | Triticum aestivum L.(Beltskaya) | 5 | 0.53 | MDA | PMP | L | -0.026 | 0.006 | 169.703 |
| 926 | Nikolaeva et al., 2010 | 55.7 | 37.6 | Triticum aestivum L.(Beltskaya) | 7 | 0.67 | MDA | PMP | L | 0.182 | 0.006 | 169.703 |
| 927 | Nikolaeva et al., 2010 | 55.7 | 37.6 | Triticum aestivum L.(Ballada) | 3 | 0.42 | Proline | NEA | L | 0.304 | 0.006 | 169.703 |
| 928 | Nikolaeva et al., 2010 | 55.7 | 37.6 | Triticum aestivum L.(Ballada) | 5 | 0.53 | Proline | NEA | L | 1.082 | 0.006 | 169.703 |
| 929 | Nikolaeva et al., 2010 | 55.7 | 37.6 | Triticum aestivum L.(Ballada) | 7 | 0.67 | Proline | NEA | L | 0.275 | 0.006 | 169.703 |
| 930 | Nikolaeva et al., 2010 | 55.7 | 37.6 | Triticum aestivum L.(Belchanka) | 3 | 0.42 | Proline | NEA | L | 0.619 | 0.006 | 169.703 |
| 931 | Nikolaeva et al., 2010 | 55.7 | 37.6 | Triticum aestivum L.(Belchanka) | 5 | 0.53 | Proline | NEA | L | 1.153 | 0.006 | 169.703 |
| 932 | Nikolaeva et al., 2010 | 55.7 | 37.6 | Triticum aestivum L.(Belchanka) | 7 | 0.67 | Proline | NEA | L | 0.447 | 0.006 | 169.703 |
| 933 | Nikolaeva et al., 2010 | 55.7 | 37.6 | Triticum aestivum L.(Beltskaya) | 3 | 0.42 | Proline | NEA | L | 0.244 | 0.006 | 169.703 |
| 934 | Nikolaeva et al., 2010 | 55.7 | 37.6 | Triticum aestivum L.(Beltskaya) | 5 | 0.53 | Proline | NEA | L | 1.229 | 0.006 | 169.703 |
| 935 | Nikolaeva et al., 2010 | 55.7 | 37.6 | Triticum aestivum L.(Beltskaya) | 7 | 0.67 | Proline | NEA | L | 0.437 | 0.006 | 169.703 |
| 936 | Nouraei et al., 2018 | 32.6 | 51.26 | Cynara cardunculus var. scolymus Hayek L. | 90 | 0.38 | CAT | EA | L | 0.119 | 0.001 | 1879.929 |
| 937 | Nouraei et al., 2018 | 32.6 | 51.26 | Cynara cardunculus var. scolymus Hayek L. | 90 | 0.75 | CAT | EA | L | 0.248 | 0.001 | 1248.876 |
| 938 | Nouraei et al., 2018 | 32.6 | 51.26 | Cynara cardunculus var. scolymus Hayek L. | 90 | 0.38 | POD | EA | L | 0.368 | 0.003 | 328.536 |
| 939 | Nouraei et al., 2018 | 32.6 | 51.26 | Cynara cardunculus var. scolymus Hayek L. | 90 | 0.75 | POD | EA | L | 0.799 | 0.003 | 333.877 |
| 940 | Nouraei et al., 2018 | 32.6 | 51.26 | Cynara cardunculus var. scolymus Hayek L. | 90 | 0.38 | APX | EA | L | 0.190 | 0.002 | 556.346 |
| 941 | Nouraei et al., 2018 | 32.6 | 51.26 | Cynara cardunculus var. scolymus Hayek L. | 90 | 0.75 | APX | EA | L | 0.633 | 0.003 | 348.570 |
| 942 | Nouraei et al., 2018 | 32.6 | 51.26 | Cynara cardunculus var. scolymus Hayek L. | 90 | 0.38 | ROS | PMP | L | 0.112 | 0.003 | 341.939 |
| 943 | Nouraei et al., 2018 | 32.6 | 51.26 | Cynara cardunculus var. scolymus Hayek L. | 90 | 0.75 | ROS | PMP | L | 0.307 | 0.003 | 380.518 |
| 944 | Nouraei et al., 2018 | 32.6 | 51.26 | Cynara cardunculus var. scolymus Hayek L. | 90 | 0.38 | MDA | PMP | L | 0.385 | 0.004 | 268.804 |
| 945 | Nouraei et al., 2018 | 32.6 | 51.26 | Cynara cardunculus var. scolymus Hayek L. | 90 | 0.75 | MDA | PMP | L | 0.793 | 0.003 | 303.034 |
| 946 | Nouraei et al., 2018 | 32.6 | 51.26 | Cynara cardunculus var. scolymus Hayek L. | 90 | 0.38 | Chl | PS | L | -0.141 | 0.002 | 483.302 |
| 947 | Nouraei et al., 2018 | 32.6 | 51.26 | Cynara cardunculus var. scolymus Hayek L. | 90 | 0.75 | Chl | PS | L | -0.350 | 0.003 | 296.451 |
| 948 | Nouraei et al., 2018 | 32.6 | 51.26 | Cynara cardunculus var. scolymus Hayek L. | 90 | 0.38 | Chl | PS | L | -0.044 | 0.002 | 623.211 |
| 949 | Nouraei et al., 2018 | 32.6 | 51.26 | Cynara cardunculus var. scolymus Hayek L. | 90 | 0.75 | Chl | PS | L | -0.245 | 0.002 | 563.494 |
| 950 | Nouraei et al., 2018 | 32.6 | 51.26 | Cynara cardunculus var. scolymus Hayek L. | 90 | 0.38 | Proline | NEA | L | 0.679 | 0.006 | 167.410 |
| 951 | Nouraei et al., 2018 | 32.6 | 51.26 | Cynara cardunculus var. scolymus Hayek L. | 90 | 0.75 | Proline | NEA | L | 1.023 | 0.006 | 180.489 |
| 952 | Nouraei et al., 2018 | 32.6 | 51.26 | Cynara cardunculus var. scolymus Hayek L. | 90 | 0.38 | Dry weight | Growth | P | -0.141 | 0.004 | 257.225 |
| 953 | Nouraei et al., 2018 | 32.6 | 51.26 | Cynara cardunculus var. scolymus Hayek L. | 90 | 0.75 | Dry weight | Growth | P | -0.214 | 0.004 | 235.533 |
| 954 | Oliveira et al., 2019 | -1.5 | -48.5 | Euterpe oleracea palms | 21 | 0.5 | Fv/Fm | PS | L | -0.025 | 0.001 | 1302.596 |
| 955 | Oliveira et al., 2019 | -1.5 | -48.5 | Euterpe oleracea palms | 34 | 0.5 | Fv/Fm | PS | L | -0.012 | 0.000 | 2504.079 |
| 956 | Oliveira et al., 2019 | -1.5 | -48.5 | Euterpe oleracea palms | 21 | 0.5 | qP | PS | L | -1.386 | 0.005 | 196.000 |
| 957 | Oliveira et al., 2019 | -1.5 | -48.5 | Euterpe oleracea palms | 34 | 0.5 | qP | PS | L | -0.693 | 0.003 | 400.000 |
| 958 | Oliveira et al., 2019 | -1.5 | -48.5 | Euterpe oleracea palms | 21 | 0.5 | MDA | PMP | L | 0.706 | 0.031 | 32.446 |
| 959 | Oliveira et al., 2019 | -1.5 | -48.5 | Euterpe oleracea palms | 34 | 0.5 | MDA | PMP | L | 0.949 | 0.010 | 97.779 |
| 960 | Ors et al., 2016 | -1.5 | -48.5 | Cucurbita pepo L. | 25 | 0.33 | EL | PMP | L | 0.163 | 0.006 | 169.703 |
| 961 | Ors et al., 2016 | -1.5 | -48.5 | Cucurbita pepo L. | 25 | 0.67 | EL | PMP | L | 0.544 | 0.006 | 169.703 |
| 962 | Ors et al., 2016 | -1.5 | -48.5 | Cucurbita pepo L. | 25 | 0.33 | Dry weight | Growth | P | -0.208 | 0.006 | 169.703 |
| 963 | Ors et al., 2016 | -1.5 | -48.5 | Cucurbita pepo L. | 25 | 0.67 | Dry weight | Growth | P | -0.992 | 0.006 | 169.703 |
| 964 | Ors et al., 2016 | -1.5 | -48.5 | Cucurbita pepo L. | 25 | 0.33 | Chl | PS | L | -0.032 | 0.006 | 169.703 |
| 965 | Ors et al., 2016 | -1.5 | -48.5 | Cucurbita pepo L. | 25 | 0.67 | Chl | PS | L | -0.076 | 0.006 | 169.703 |
| 966 | Parveen et al., 2019 | 31.4 | 73.1 | Zea mays L.(variety Pearl) | 21 | 0.4 | Dry weight | Growth | S | -0.167 | 0.005 | 211.541 |
| 967 | Parveen et al., 2019 | 31.4 | 73.1 | Zea mays L.(variety Malka) | 21 | 0.4 | Dry weight | Growth | S | -0.297 | 0.002 | 632.298 |
| 968 | Parveen et al., 2019 | 31.4 | 73.1 | Zea mays L.(variety Pearl) | 21 | 0.4 | Dry weight | Growth | R | -0.762 | 0.013 | 78.563 |
| 969 | Parveen et al., 2019 | 31.4 | 73.1 | Zea mays L.(variety Malka) | 21 | 0.4 | Dry weight | Growth | R | -0.325 | 0.019 | 53.247 |
| 970 | Parveen et al., 2019 | 31.4 | 73.1 | Zea mays L.(variety Pearl) | 21 | 0.4 | Chl | Growth | L | -0.311 | 0.003 | 361.981 |
| 971 | Parveen et al., 2019 | 31.4 | 73.1 | Zea mays L.(variety Malka) | 21 | 0.4 | Chl | Growth | L | -0.187 | 0.005 | 193.466 |
| 972 | Parveen et al., 2019 | 31.4 | 73.1 | Zea mays L.(variety Pearl) | 21 | 0.4 | Chl | Growth | L | -0.578 | 0.018 | 55.257 |
| 973 | Parveen et al., 2019 | 31.4 | 73.1 | Zea mays L.(variety Malka) | 21 | 0.4 | Chl | Growth | L | -0.421 | 0.002 | 438.715 |
| 974 | Parveen et al., 2019 | 31.4 | 73.1 | Zea mays L.(variety Pearl) | 21 | 0.4 | Proline | NEA | L | 0.366 | 0.002 | 413.443 |
| 975 | Parveen et al., 2019 | 31.4 | 73.1 | Zea mays L.(variety Malka) | 21 | 0.4 | Proline | NEA | L | 0.397 | 0.003 | 371.171 |
| 976 | Parveen et al., 2019 | 31.4 | 73.1 | Zea mays L.(variety Pearl) | 21 | 0.4 | Soluble sugar | NEA | L | 0.486 | 0.009 | 112.246 |
| 977 | Parveen et al., 2019 | 31.4 | 73.1 | Zea mays L.(variety Malka) | 21 | 0.4 | Soluble sugar | NEA | L | 0.347 | 0.002 | 588.699 |
| 978 | Parveen et al., 2019 | 31.4 | 73.1 | Zea mays L.(variety Pearl) | 21 | 0.4 | CAT | EA | L | 0.305 | 0.003 | 380.248 |
| 979 | Parveen et al., 2019 | 31.4 | 73.1 | Zea mays L.(variety Malka) | 21 | 0.4 | CAT | EA | L | 0.234 | 0.002 | 563.755 |
| 980 | Parveen et al., 2019 | 31.4 | 73.1 | Zea mays L.(variety Pearl) | 21 | 0.4 | SOD | EA | L | 0.241 | 0.012 | 83.045 |
| 981 | Parveen et al., 2019 | 31.4 | 73.1 | Zea mays L.(variety Malka) | 21 | 0.4 | SOD | EA | L | 0.248 | 0.005 | 184.536 |
| 982 | Parveen et al., 2019 | 31.4 | 73.1 | Zea mays L.(variety Pearl) | 21 | 0.4 | POD | EA | L | 0.097 | 0.004 | 275.905 |
| 983 | Parveen et al., 2019 | 31.4 | 73.1 | Zea mays L.(variety Malka) | 21 | 0.4 | POD | EA | L | -0.036 | 0.001 | 710.270 |
| 984 | Parveen et al., 2019 | 31.4 | 73.1 | Zea mays L.(variety Pearl) | 21 | 0.4 | MDA | PMP | L | 0.288 | 0.013 | 78.505 |
| 985 | Parveen et al., 2019 | 31.4 | 73.1 | Zea mays L.(variety Malka) | 21 | 0.4 | MDA | PMP | L | 0.473 | 0.019 | 53.489 |
| 986 | Parveen et al., 2019 | 31.4 | 73.1 | Zea mays L.(variety Pearl) | 21 | 0.4 | ROS | PMP | L | 0.827 | 0.010 | 102.878 |
| 987 | Parveen et al., 2019 | 31.4 | 73.1 | Zea mays L.(variety Malka) | 21 | 0.4 | ROS | PMP | L | 0.739 | 0.010 | 97.535 |
| 988 | Petridis et al., 2012 | 40.7 | 22.9 | Olea europaea L. Gaidourelia | 30 | 0.34 | Fv/Fm | PS | L | -0.039 | 0.001 | 897.946 |
| 989 | Petridis et al., 2012 | 40.7 | 22.9 | Olea europaea L. Kalamon | 30 | 0.34 | Fv/Fm | PS | L | 0.013 | 0.004 | 267.911 |
| 990 | Petridis et al., 2012 | 40.7 | 22.9 | Olea europaea L. Koroneiki | 30 | 0.34 | Fv/Fm | PS | L | -0.013 | 0.006 | 169.703 |
| 991 | Petridis et al., 2012 | 40.7 | 22.9 | Olea europaea L. Megaritiki | 30 | 0.34 | Fv/Fm | PS | L | 0.013 | 0.006 | 169.703 |
| 992 | Petridis et al., 2012 | 40.7 | 22.9 | Olea europaea L. Gaidourelia | 30 | 0.67 | Fv/Fm | PS | L | -0.230 | 0.001 | 817.343 |
| 993 | Petridis et al., 2012 | 40.7 | 22.9 | Olea europaea L. Kalamon | 30 | 0.67 | Fv/Fm | PS | L | -0.329 | 0.015 | 66.966 |
| 994 | Petridis et al., 2012 | 40.7 | 22.9 | Olea europaea L. Koroneiki | 30 | 0.67 | Fv/Fm | PS | L | -0.053 | 0.005 | 197.413 |
| 995 | Petridis et al., 2012 | 40.7 | 22.9 | Olea europaea L. Megaritiki | 30 | 0.67 | Fv/Fm | PS | L | -0.082 | 0.004 | 280.130 |
| 996 | Petridis et al., 2012 | 40.7 | 22.9 | Olea europaea L. Gaidourelia | 60 | 0.34 | Fv/Fm | PS | L | 0.000 | 0.002 | 408.394 |
| 997 | Petridis et al., 2012 | 40.7 | 22.9 | Olea europaea L. Kalamon | 60 | 0.34 | Fv/Fm | PS | L | 0.038 | 0.000 | 3779.944 |
| 998 | Petridis et al., 2012 | 40.7 | 22.9 | Olea europaea L.Koroneiki | 60 | 0.34 | Fv/Fm | PS | L | 0.000 | 0.002 | 408.643 |
| 999 | Petridis et al., 2012 | 40.7 | 22.9 | Olea europaea L. Megaritiki | 60 | 0.34 | Fv/Fm | PS | L | 0.013 | 0.003 | 382.294 |
| 1000 | Petridis et al., 2012 | 40.7 | 22.9 | Olea europaea L. Gaidourelia | 60 | 0.67 | Fv/Fm | PS | L | -0.013 | 0.002 | 408.394 |
| 1001 | Petridis et al., 2012 | 40.7 | 22.9 | Olea europaea L. Kalamon | 60 | 0.67 | Fv/Fm | PS | L | -0.201 | 0.019 | 52.763 |
| 1002 | Petridis et al., 2012 | 40.7 | 22.9 | Olea europaea L. Koroneiki | 60 | 0.67 | Fv/Fm | PS | L | -0.154 | 0.008 | 129.727 |
| 1003 | Petridis et al., 2012 | 40.7 | 22.9 | Olea europaea L. Megaritiki | 60 | 0.67 | Fv/Fm | PS | L | -0.169 | 0.005 | 189.602 |
| 1004 | Petridis et al., 2012 | 40.7 | 22.9 | Olea europaea L. Gaidourelia | 30 | 0.34 | MDA | PMP | L | -0.023 | 0.001 | 998.795 |
| 1005 | Petridis et al., 2012 | 40.7 | 22.9 | Olea europaea L. Kalamon | 30 | 0.34 | MDA | PMP | L | 0.003 | 0.001 | 981.876 |
| 1006 | Petridis et al., 2012 | 40.7 | 22.9 | Olea europaea L. Koroneiki | 30 | 0.34 | MDA | PMP | L | 0.129 | 0.002 | 561.766 |
| 1007 | Petridis et al., 2012 | 40.7 | 22.9 | Olea europaea L. Megaritiki | 30 | 0.34 | MDA | PMP | L | -0.003 | 0.004 | 227.119 |
| 1008 | Petridis et al., 2012 | 40.7 | 22.9 | Olea europaea L. Gaidourelia | 30 | 0.67 | MDA | PMP | L | -0.098 | 0.002 | 531.489 |
| 1009 | Petridis et al., 2012 | 40.7 | 22.9 | Olea europaea L. Kalamon | 30 | 0.67 | MDA | PMP | L | 0.051 | 0.001 | 999.788 |
| 1010 | Petridis et al., 2012 | 40.7 | 22.9 | Olea europaea L. Koroneiki | 30 | 0.67 | MDA | PMP | L | 0.165 | 0.002 | 529.667 |
| 1011 | Petridis et al., 2012 | 40.7 | 22.9 | Olea europaea L. Megaritiki | 30 | 0.67 | MDA | PMP | L | -0.014 | 0.008 | 132.043 |
| 1012 | Petridis et al., 2012 | 40.7 | 22.9 | Olea europaea L. Gaidourelia | 60 | 0.34 | MDA | PMP | L | 0.182 | 0.000 | 5443.785 |
| 1013 | Petridis et al., 2012 | 40.7 | 22.9 | Olea europaea L. Kalamon | 60 | 0.34 | MDA | PMP | L | 0.270 | 0.001 | 1848.262 |
| 1014 | Petridis et al., 2012 | 40.7 | 22.9 | Olea europaea L. Koroneiki | 60 | 0.34 | MDA | PMP | L | 0.068 | 0.000 | 4019.547 |
| 1015 | Petridis et al., 2012 | 40.7 | 22.9 | Olea europaea L. Megaritiki | 60 | 0.34 | MDA | PMP | L | 0.050 | 0.000 | 4179.114 |
| 1016 | Petridis et al., 2012 | 40.7 | 22.9 | Olea europaea L. Gaidourelia | 60 | 0.67 | MDA | PMP | L | 0.238 | 0.000 | 4035.429 |
| 1017 | Petridis et al., 2012 | 40.7 | 22.9 | Olea europaea L. Kalamon | 60 | 0.67 | MDA | PMP | L | 0.511 | 0.001 | 1875.209 |
| 1018 | Petridis et al., 2012 | 40.7 | 22.9 | Olea europaea L. Koroneiki | 60 | 0.67 | MDA | PMP | L | 0.263 | 0.000 | 3274.391 |
| 1019 | Petridis et al., 2012 | 40.7 | 22.9 | Olea europaea L. Megaritiki | 60 | 0.67 | MDA | PMP | L | 0.152 | 0.000 | 3541.542 |
| 1020 | Plazas et al., 2019 | 39.5 | -0.4 | Solanum melongena L. | 11 | 0.7 | Dry weight | Growth | S | -2.159 | 0.046 | 21.609 |
| 1021 | Plazas et al., 2019 | 39.5 | -0.4 | Solanum melongena L. | 11 | 0.7 | Dry weight | Growth | R | -1.420 | 0.006 | 154.317 |
| 1022 | Plazas et al., 2019 | 39.5 | -0.4 | Solanum melongena L. | 11 | 0.7 | Dry weight | Growth | L | -1.479 | 0.007 | 136.753 |
| 1023 | Plazas et al., 2019 | 39.5 | -0.4 | Solanum melongena L. | 11 | 0.7 | Chl | PS | L | -1.049 | 0.005 | 196.916 |
| 1024 | Plazas et al., 2019 | 39.5 | -0.4 | Solanum melongena L. | 11 | 0.7 | Chl | PS | L | -0.865 | 0.015 | 65.078 |
| 1025 | Plazas et al., 2019 | 39.5 | -0.4 | Solanum melongena L. | 11 | 0.7 | Proline | NEA | L | 3.166 | 0.007 | 135.594 |
| 1026 | Plazas et al., 2019 | 39.5 | -0.4 | Solanum melongena L. | 11 | 0.7 | MDA | PMP | L | 0.192 | 0.002 | 422.739 |
| 1027 | Plazas et al., 2019 | 39.5 | -0.4 | Solanum melongena L. | 11 | 0.7 | SOD | EA | L | 0.288 | 0.014 | 70.611 |
| 1028 | Plazas et al., 2019 | 39.5 | -0.4 | Solanum melongena L. | 11 | 0.7 | CAT | EA | L | 1.046 | 0.061 | 16.378 |
| 1029 | Plazas et al., 2019 | 39.5 | -0.4 | Solanum melongena L. | 11 | 0.7 | APX | EA | L | 0.450 | 0.031 | 32.196 |
| 1030 | Plazas et al., 2019 | 39.5 | -0.4 | Solanum melongena L. | 11 | 0.7 | GR | EA | L | -0.331 | 0.002 | 606.508 |
| 1031 | Pompelli et al., 2010 | -9.5 | -35.8 | Jatropha curcas L. | 4 | 0.4 | Chl | PS | L | -0.107 | 0.001 | 857.736 |
| 1032 | Pompelli et al., 2010 | -9.5 | -35.8 | Jatropha curcas L. | 8 | 0.7 | Chl | PS | L | -0.281 | 0.002 | 472.168 |
| 1033 | Pompelli et al., 2010 | -9.5 | -35.8 | Jatropha curcas L. | 18 | 0.8 | Chl | PS | L | -0.722 | 0.004 | 284.497 |
| 1034 | Pompelli et al., 2010 | -9.5 | -35.8 | Jatropha curcas L. | 4 | 0.4 | Chl | PS | L | 0.117 | 0.002 | 429.177 |
| 1035 | Pompelli et al., 2010 | -9.5 | -35.8 | Jatropha curcas L. | 8 | 0.7 | Chl | PS | L | -0.207 | 0.002 | 624.899 |
| 1036 | Pompelli et al., 2010 | -9.5 | -35.8 | Jatropha curcas L. | 18 | 0.8 | Chl | PS | L | -0.492 | 0.005 | 206.728 |
| 1037 | Pompelli et al., 2010 | -9.5 | -35.8 | Jatropha curcas L. | 4 | 0.4 | Protein | Growth | L | -0.321 | 0.003 | 309.856 |
| 1038 | Pompelli et al., 2010 | -9.5 | -35.8 | Jatropha curcas L. | 8 | 0.7 | Protein | Growth | L | -0.901 | 0.006 | 164.595 |
| 1039 | Pompelli et al., 2010 | -9.5 | -35.8 | Jatropha curcas L. | 18 | 0.8 | Protein | Growth | L | -0.214 | 0.002 | 500.106 |
| 1040 | Pompelli et al., 2010 | -9.5 | -35.8 | Jatropha curcas L. | 4 | 0.4 | SOD | EA | L | 0.401 | 0.014 | 70.770 |
| 1041 | Pompelli et al., 2010 | -9.5 | -35.8 | Jatropha curcas L. | 8 | 0.7 | SOD | EA | L | 1.073 | 0.006 | 160.328 |
| 1042 | Pompelli et al., 2010 | -9.5 | -35.8 | Jatropha curcas L. | 18 | 0.8 | SOD | EA | L | 0.546 | 0.005 | 192.766 |
| 1043 | Pompelli et al., 2010 | -9.5 | -35.8 | Jatropha curcas L. | 4 | 0.4 | CAT | EA | L | 0.251 | 0.007 | 152.654 |
| 1044 | Pompelli et al., 2010 | -9.5 | -35.8 | Jatropha curcas L. | 8 | 0.7 | CAT | EA | L | 0.336 | 0.003 | 331.081 |
| 1045 | Pompelli et al., 2010 | -9.5 | -35.8 | Jatropha curcas L. | 18 | 0.8 | CAT | EA | L | 0.182 | 0.002 | 663.934 |
| 1046 | Pompelli et al., 2010 | -9.5 | -35.8 | Jatropha curcas L. | 4 | 0.4 | APX | EA | L | -0.280 | 0.003 | 291.137 |
| 1047 | Pompelli et al., 2010 | -9.5 | -35.8 | Jatropha curcas L. | 8 | 0.7 | APX | EA | L | 0.493 | 0.002 | 484.673 |
| 1048 | Pompelli et al., 2010 | -9.5 | -35.8 | Jatropha curcas L. | 18 | 0.8 | APX | EA | L | 0.371 | 0.008 | 129.962 |
| 1049 | Pompelli et al., 2010 | -9.5 | -35.8 | Jatropha curcas L. | 4 | 0.4 | MDA | PMP | L | 0.167 | 0.000 | 3853.467 |
| 1050 | Pompelli et al., 2010 | -9.5 | -35.8 | Jatropha curcas L. | 8 | 0.7 | MDA | PMP | L | 0.230 | 0.000 | 3768.513 |
| 1051 | Pompelli et al., 2010 | -9.5 | -35.8 | Jatropha curcas L. | 18 | 0.8 | MDA | PMP | L | 0.453 | 0.001 | 1537.514 |
| 1052 | Pourghayoumi et al., 2017 | 29.9 | 52 | pomegranate | 14 | 0.7 | Chl | PS | L | -0.424 | 0.057 | 17.522 |
| 1053 | Pourghayoumi et al., 2017 | 29.9 | 52 | pomegranate | 14 | 0.7 | Chl | PS | L | -0.389 | 0.001 | 1544.272 |
| 1054 | Pourghayoumi et al., 2017 | 29.9 | 52 | pomegranate | 14 | 0.7 | SOD | EA | L | 0.393 | 0.000 | 3196.028 |
| 1055 | Pourghayoumi et al., 2017 | 29.9 | 52 | pomegranate | 14 | 0.7 | SOD | EA | L | 0.271 | 0.000 | 2652.991 |
| 1056 | Pourghayoumi et al., 2017 | 29.9 | 52 | pomegranate | 14 | 0.7 | CAT | EA | L | 0.773 | 0.008 | 126.724 |
| 1057 | Pourghayoumi et al., 2017 | 29.9 | 52 | pomegranate | 14 | 0.7 | CAT | EA | L | 0.624 | 0.005 | 209.938 |
| 1058 | Pourghayoumi et al., 2017 | 29.9 | 52 | pomegranate | 14 | 0.7 | MDA | PMP | L | 0.147 | 0.001 | 900.204 |
| 1059 | Pourghayoumi et al., 2017 | 29.9 | 52 | pomegranate | 14 | 0.7 | MDA | PMP | L | 0.252 | 0.001 | 1409.927 |
| 1060 | Pourghayoumi et al., 2017 | 29.9 | 52 | pomegranate | 14 | 0.7 | Proline | NEA | L | 0.960 | 0.006 | 165.998 |
| 1061 | Pourghayoumi et al., 2017 | 29.9 | 52 | pomegranate | 14 | 0.7 | Proline | NEA | L | 0.942 | 0.007 | 148.499 |
| 1062 | Rigui et al., 2019 | -23.6 | -46.6 | Lolium perenne L. cv. AberAvon | 23 | 0.75 | Dry weight | Growth | L | 0.405 | 0.030 | 33.231 |
| 1063 | Rigui et al., 2019 | -23.6 | -46.6 | Lolium perenne L. cv. AberAvon | 23 | 0.75 | Chl | PS | L | -0.961 | 0.018 | 54.734 |
| 1064 | Rigui et al., 2019 | -23.6 | -46.6 | Lolium perenne L. cv. AberAvon | 23 | 0.75 | Chl | PS | L | -0.803 | 0.006 | 166.485 |
| 1065 | Rigui et al., 2019 | -23.6 | -46.6 | Lolium perenne L. cv. AberAvon | 23 | 0.75 | ROS | PMP | L | -0.570 | 0.026 | 38.364 |
| 1066 | Rigui et al., 2019 | -23.6 | -46.6 | Lolium perenne L. cv. AberAvon | 23 | 0.75 | MDA | PMP | L | -0.083 | 0.024 | 41.774 |
| 1067 | Rigui et al., 2019 | -23.6 | -46.6 | Lolium perenne L. cv. AberAvon | 23 | 0.75 | AsA | NEA | L | -0.473 | 0.012 | 85.260 |
| 1068 | Rigui et al., 2019 | -23.6 | -46.6 | Lolium perenne L. cv. AberAvon | 23 | 0.75 | APX | EA | L | -0.321 | 0.020 | 49.670 |
| 1069 | Rigui et al., 2019 | -23.6 | -46.6 | Lolium perenne L. cv. AberAvon | 23 | 0.75 | GR | EA | L | 0.236 | 0.001 | 1663.311 |
| 1070 | Rolando et al., 2015 | -12.1 | -77 | Solanum tuberosum L. | 20 | 0.5 | Chl | PS | L | 0.066 | 0.000 | 14353.959 |
| 1071 | Rolando et al., 2015 | -12.1 | -77 | Solanum tuberosum L. | 40 | 0.5 | Chl | PS | L | 0.047 | 0.000 | 10519.451 |
| 1072 | Rolando et al., 2015 | -12.1 | -77 | Solanum tuberosum L. | 60 | 0.5 | Chl | PS | L | 0.000 | 0.000 | 8880.320 |
| 1073 | Rolando et al., 2015 | -12.1 | -77 | Solanum tuberosum L. | 80 | 0.5 | Chl | PS | L | 0.000 | 0.003 | 351.655 |
| 1074 | Samota et al., 2017 | 28.7 | 76.9 | Oryza sativa L. | 6 | 0.55 | Proline | NEA | L | 0.357 | 0.001 | 1578.523 |
| 1075 | Samota et al., 2017 | 28.7 | 76.9 | Oryza sativa L. | 6 | 0.55 | Proline | NEA | L | 0.556 | 0.001 | 848.884 |
| 1076 | Samota et al., 2017 | 28.7 | 76.9 | Oryza sativa L. | 6 | 0.55 | SOD | EA | L | 0.106 | 0.000 | 812362.346 |
| 1077 | Samota et al., 2017 | 28.7 | 76.9 | Oryza sativa L. | 6 | 0.55 | SOD | EA | L | 0.153 | 0.000 | 156638.762 |
| 1078 | Samota et al., 2017 | 28.7 | 76.9 | Oryza sativa L. | 6 | 0.55 | CAT | EA | L | 0.226 | 0.009 | 114.518 |
| 1079 | Samota et al., 2017 | 28.7 | 76.9 | Oryza sativa L. | 6 | 0.55 | CAT | EA | L | 0.380 | 0.006 | 163.860 |
| 1080 | Samota et al., 2017 | 28.7 | 76.9 | Oryza sativa L. | 6 | 0.55 | POD | EA | L | 0.045 | 0.000 | 5458.858 |
| 1081 | Samota et al., 2017 | 28.7 | 76.9 | Oryza sativa L. | 6 | 0.55 | POD | EA | L | 0.018 | 0.000 | 5109.084 |
| 1082 | Sanchez-Rodriguez et al., 2012 | 37.2 | -3.6 | Lycopersicon esculentum Mill | 22 | 0.5 | Chl | PS | L | -0.479 | 0.044 | 22.767 |
| 1083 | Sanchez-Rodriguez et al., 2012 | 37.2 | -3.6 | Lycopersicon esculentum Mill | 22 | 0.5 | Chl | PS | L | -0.635 | 0.030 | 32.978 |
| 1084 | Sanchez-Rodriguez et al., 2012 | 37.2 | -3.6 | Lycopersicon esculentum Mill | 22 | 0.5 | ROS | PMP | L | 0.589 | 0.039 | 25.338 |
| 1085 | Sanchez-Rodriguez et al., 2012 | 37.2 | -3.6 | Lycopersicon esculentum Mill | 22 | 0.5 | ROS | PMP | L | 0.315 | 0.005 | 203.618 |
| 1086 | Sanchez-Rodriguez et al., 2012 | 37.2 | -3.6 | Lycopersicon esculentum Mill | 22 | 0.5 | SOD | EA | L | 0.687 | 0.022 | 46.043 |
| 1087 | Sanchez-Rodriguez et al., 2012 | 37.2 | -3.6 | Lycopersicon esculentum Mill | 22 | 0.5 | CAT | EA | L | 0.460 | 0.001 | 683.001 |
| 1088 | Sanchez-Rodriguez et al., 2012 | 37.2 | -3.6 | Lycopersicon esculentum Mill | 22 | 0.5 | GR | EA | L | 0.081 | 0.018 | 56.325 |
| 1089 | Sanchez-Rodriguez et al., 2012 | 37.2 | -3.6 | Lycopersicon esculentum Mill | 22 | 0.5 | MDA | PMP | L | 0.727 | 0.004 | 258.967 |
| 1090 | Sedaghat et al., 2017 | 29.4 | 52.8 | Triticum aestivum L. | 45 | 0.5 | EL | PMP | L | 0.938 | 0.014 | 73.200 |
| 1091 | Sedaghat et al., 2017 | 29.4 | 52.8 | Triticum aestivum L. | 45 | 0.5 | EL | PMP | L | 1.168 | 0.056 | 17.905 |
| 1092 | Sedaghat et al., 2017 | 29.4 | 52.8 | Triticum aestivum L. | 45 | 0.5 | CAT | EA | L | -1.708 | 0.040 | 24.847 |
| 1093 | Sedaghat et al., 2017 | 29.4 | 52.8 | Triticum aestivum L. | 45 | 0.5 | CAT | EA | L | -1.721 | 0.014 | 70.534 |
| 1094 | Sedaghat et al., 2017 | 29.4 | 52.8 | Triticum aestivum L. | 45 | 0.5 | APX | EA | L | 0.553 | 0.009 | 111.270 |
| 1095 | Sedaghat et al., 2017 | 29.4 | 52.8 | Triticum aestivum L. | 45 | 0.5 | APX | EA | L | 0.357 | 0.040 | 25.157 |
| 1096 | Sedaghat et al., 2017 | 29.4 | 52.8 | Triticum aestivum L. | 45 | 0.5 | POD | EA | L | 0.666 | 0.002 | 587.183 |
| 1097 | Sedaghat et al., 2017 | 29.4 | 52.8 | Triticum aestivum L. | 45 | 0.5 | POD | EA | L | 0.119 | 0.004 | 283.618 |
| 1098 | Sedaghat et al., 2017 | 29.4 | 52.8 | Triticum aestivum L. | 45 | 0.5 | SOD | EA | L | 0.505 | 0.012 | 83.407 |
| 1099 | Sedaghat et al., 2017 | 29.4 | 52.8 | Triticum aestivum L. | 45 | 0.5 | SOD | EA | L | 0.422 | 0.009 | 114.746 |
| 1100 | Sedaghat et al., 2017 | 29.4 | 52.8 | Triticum aestivum L. | 45 | 0.5 | MDA | PMP | L | 0.842 | 0.005 | 193.855 |
| 1101 | Sedaghat et al., 2017 | 29.4 | 52.8 | Triticum aestivum L. | 45 | 0.5 | MDA | PMP | L | 1.141 | 0.003 | 315.450 |
| 1102 | Sharifi et al., 2016 | 37.5 | 45.1 | Triticum aestivum L. | 27 | 0.25 | Chl | PS | L | -0.069 | 0.049 | 20.332 |
| 1103 | Sharifi et al., 2016 | 37.5 | 45.1 | Triticum aestivum L. | 27 | 0.63 | Chl | PS | L | -0.458 | 0.017 | 58.157 |
| 1104 | Sharifi et al., 2016 | 37.5 | 45.1 | Triticum aestivum L. | 27 | 0.76 | Chl | PS | L | -1.148 | 0.154 | 6.509 |
| 1105 | Sharifi et al., 2016 | 37.5 | 45.1 | Triticum aestivum L. | 27 | 0.25 | Chl | PS | L | -0.033 | 0.050 | 20.049 |
| 1106 | Sharifi et al., 2016 | 37.5 | 45.1 | Triticum aestivum L. | 27 | 0.63 | Chl | PS | L | -0.445 | 0.021 | 47.866 |
| 1107 | Sharifi et al., 2016 | 37.5 | 45.1 | Triticum aestivum L. | 27 | 0.76 | Chl | PS | L | -1.064 | 0.101 | 9.895 |
| 1108 | Sharifi et al., 2016 | 37.5 | 45.1 | Triticum aestivum L. | 27 | 0.25 | Chl | PS | L | -0.121 | 0.001 | 956.072 |
| 1109 | Sharifi et al., 2016 | 37.5 | 45.1 | Triticum aestivum L. | 27 | 0.63 | Chl | PS | L | -0.364 | 0.002 | 576.921 |
| 1110 | Sharifi et al., 2016 | 37.5 | 45.1 | Triticum aestivum L. | 27 | 0.76 | Chl | PS | L | -0.648 | 0.002 | 559.232 |
| 1111 | Sharifi et al., 2016 | 37.5 | 45.1 | Triticum aestivum L. | 27 | 0.25 | Chl | PS | L | -0.099 | 0.001 | 852.858 |
| 1112 | Sharifi et al., 2016 | 37.5 | 45.1 | Triticum aestivum L. | 27 | 0.63 | Chl | PS | L | -0.402 | 0.001 | 688.196 |
| 1113 | Sharifi et al., 2016 | 37.5 | 45.1 | Triticum aestivum L. | 27 | 0.76 | Chl | PS | L | -0.640 | 0.002 | 586.638 |
| 1114 | Sharifi et al., 2016 | 37.5 | 45.1 | Triticum aestivum L. | 27 | 0.25 | ABA | NEA | L | 0.226 | 0.002 | 463.211 |
| 1115 | Sharifi et al., 2016 | 37.5 | 45.1 | Triticum aestivum L. | 27 | 0.63 | ABA | NEA | L | 0.919 | 0.002 | 538.621 |
| 1116 | Sharifi et al., 2016 | 37.5 | 45.1 | Triticum aestivum L. | 27 | 0.76 | ABA | NEA | L | 1.212 | 0.002 | 516.297 |
| 1117 | Sharifi et al., 2016 | 37.5 | 45.1 | Triticum aestivum L. | 27 | 0.25 | ABA | NEA | L | 0.009 | 0.003 | 371.892 |
| 1118 | Sharifi et al., 2016 | 37.5 | 45.1 | Triticum aestivum L. | 27 | 0.63 | ABA | NEA | L | 1.050 | 0.002 | 527.454 |
| 1119 | Sharifi et al., 2016 | 37.5 | 45.1 | Triticum aestivum L. | 27 | 0.76 | ABA | NEA | L | 1.110 | 0.002 | 498.078 |
| 1120 | Sharifi et al., 2016 | 37.5 | 45.1 | Triticum aestivum L. | 27 | 0.25 | Protein | Growth | L | 0.283 | 0.000 | 3110.673 |
| 1121 | Sharifi et al., 2016 | 37.5 | 45.1 | Triticum aestivum L. | 27 | 0.63 | Protein | Growth | L | 0.440 | 0.000 | 3242.397 |
| 1122 | Sharifi et al., 2016 | 37.5 | 45.1 | Triticum aestivum L. | 27 | 0.76 | Protein | Growth | L | 0.207 | 0.000 | 3107.843 |
| 1123 | Sharifi et al., 2016 | 37.5 | 45.1 | Triticum aestivum L. | 27 | 0.25 | Protein | Growth | L | 0.180 | 0.002 | 529.707 |
| 1124 | Sharifi et al., 2016 | 37.5 | 45.1 | Triticum aestivum L. | 27 | 0.63 | Protein | Growth | L | 0.331 | 0.002 | 531.473 |
| 1125 | Sharifi et al., 2016 | 37.5 | 45.1 | Triticum aestivum L. | 27 | 0.76 | Protein | Growth | L | 0.106 | 0.002 | 527.635 |
| 1126 | Shawon et al., 2020 | 35.9 | 126.9 | Brassica rapa | 7 | 0.666667 | Dry weight | Growth | P | -0.092 | 0.004 | 282.838 |
| 1127 | Shawon et al., 2020 | 35.9 | 126.9 | Brassica rapa | 14 | 0.666667 | Dry weight | Growth | P | -0.234 | 0.004 | 282.838 |
| 1128 | Shawon et al., 2020 | 35.9 | 126.9 | Brassica rapa | 21 | 0.666667 | Dry weight | Growth | P | -0.260 | 0.004 | 282.838 |
| 1129 | Shawon et al., 2020 | 35.9 | 126.9 | Brassica rapa | 7 | 0.666667 | Dry weight | Growth | L | -0.116 | 0.004 | 282.838 |
| 1130 | Shawon et al., 2020 | 35.9 | 126.9 | Brassica rapa | 14 | 0.666667 | Dry weight | Growth | L | -0.158 | 0.004 | 282.838 |
| 1131 | Shawon et al., 2020 | 35.9 | 126.9 | Brassica rapa | 21 | 0.666667 | Dry weight | Growth | L | -0.105 | 0.004 | 282.838 |
| 1132 | Shawon et al., 2020 | 35.9 | 126.9 | Brassica rapa | 7 | 0.666667 | CAT | EA | L | 1.054 | 0.004 | 282.838 |
| 1133 | Shawon et al., 2020 | 35.9 | 126.9 | Brassica rapa | 14 | 0.666667 | CAT | EA | L | -0.634 | 0.004 | 282.838 |
| 1134 | Shawon et al., 2020 | 35.9 | 126.9 | Brassica rapa | 21 | 0.666667 | CAT | EA | L | -0.042 | 0.004 | 282.838 |
| 1135 | Shawon et al., 2020 | 35.9 | 126.9 | Brassica rapa | 7 | 0.666667 | APX | EA | L | 0.500 | 0.004 | 282.838 |
| 1136 | Shawon et al., 2020 | 35.9 | 126.9 | Brassica rapa | 14 | 0.666667 | APX | EA | L | -0.066 | 0.004 | 282.838 |
| 1137 | Shawon et al., 2020 | 35.9 | 126.9 | Brassica rapa | 21 | 0.666667 | APX | EA | L | -0.223 | 0.004 | 282.838 |
| 1138 | Shawon et al., 2020 | 35.9 | 126.9 | Brassica rapa | 7 | 0.666667 | SOD | EA | L | -0.044 | 0.004 | 282.838 |
| 1139 | Shawon et al., 2020 | 35.9 | 126.9 | Brassica rapa | 14 | 0.666667 | SOD | EA | L | -0.072 | 0.004 | 282.838 |
| 1140 | Shawon et al., 2020 | 35.9 | 126.9 | Brassica rapa | 21 | 0.666667 | SOD | EA | L | -0.045 | 0.004 | 282.838 |
| 1141 | Shawon et al., 2020 | 35.9 | 126.9 | Brassica rapa | 7 | 0.666667 | POD | EA | L | -0.085 | 0.004 | 282.838 |
| 1142 | Shawon et al., 2020 | 35.9 | 126.9 | Brassica rapa | 14 | 0.666667 | POD | EA | L | -0.245 | 0.004 | 282.838 |
| 1143 | Shawon et al., 2020 | 35.9 | 126.9 | Brassica rapa | 21 | 0.666667 | POD | EA | L | -0.347 | 0.004 | 282.838 |
| 1144 | Sheoran et al., 2015 | 21.6 | 80.2 | Triticum aestivum L. | 10 | 0.25 | Dry weight | Growth | P | -0.073 | 0.005 | 197.994 |
| 1145 | Sheoran et al., 2015 | 21.6 | 80.2 | Triticum aestivum L. | 10 | 0.55 | Dry weight | Growth | P | -0.087 | 0.004 | 280.912 |
| 1146 | Sheoran et al., 2015 | 21.6 | 80.2 | Triticum aestivum L. | 10 | 0.25 | Dry weight | Growth | P | -0.061 | 0.003 | 337.258 |
| 1147 | Sheoran et al., 2015 | 21.6 | 80.2 | Triticum aestivum L. | 10 | 0.55 | Dry weight | Growth | P | -0.072 | 0.001 | 714.067 |
| 1148 | Sheoran et al., 2015 | 21.6 | 80.2 | Triticum aestivum L. | 1 | 0.55 | SOD | EA | L | 0.203 | 0.003 | 358.597 |
| 1149 | Sheoran et al., 2015 | 21.6 | 80.2 | Triticum aestivum L. | 10 | 0.55 | SOD | EA | L | 0.267 | 0.937 | 1.067 |
| 1150 | Sheoran et al., 2015 | 21.6 | 80.2 | Triticum aestivum L. | 1 | 0.55 | CAT | EA | L | -0.125 | 0.013 | 75.831 |
| 1151 | Sheoran et al., 2015 | 21.6 | 80.2 | Triticum aestivum L. | 10 | 0.55 | CAT | EA | L | -0.460 | 0.020 | 49.213 |
| 1152 | Sheoran et al., 2015 | 21.6 | 80.2 | Triticum aestivum L. | 1 | 0.55 | POD | EA | L | 0.413 | 0.003 | 331.567 |
| 1153 | Sheoran et al., 2015 | 21.6 | 80.2 | Triticum aestivum L. | 10 | 0.55 | POD | EA | L | 0.607 | 0.002 | 581.113 |
| 1154 | Sheoran et al., 2015 | 21.6 | 80.2 | Triticum aestivum L. | 1 | 0.55 | MDA | EA | L | -0.078 | 0.008 | 121.498 |
| 1155 | Sheoran et al., 2015 | 21.6 | 80.2 | Triticum aestivum L. | 10 | 0.55 | MDA | EA | L | 0.188 | 0.010 | 101.169 |
| 1156 | Sheshbahreh et al., 2019 | 31.5 | 50.8 | Echinacea purpurea (L.) | 90 | 0.3 | Protein | Growth | L | -0.009 | 0.004 | 239.452 |
| 1157 | Sheshbahreh et al., 2019 | 31.5 | 50.8 | Echinacea purpurea (L.) | 90 | 0.6 | Protein | Growth | L | -0.199 | 0.004 | 227.668 |
| 1158 | Sheshbahreh et al., 2019 | 31.5 | 50.8 | Echinacea purpurea (L.) | 90 | 0.3 | CAT | EA | L | 0.482 | 0.006 | 169.703 |
| 1159 | Sheshbahreh et al., 2019 | 31.5 | 50.8 | Echinacea purpurea (L.) | 90 | 0.6 | CAT | EA | L | 0.938 | 0.006 | 169.703 |
| 1160 | Sheshbahreh et al., 2019 | 31.5 | 50.8 | Echinacea purpurea (L.) | 90 | 0.3 | POD | EA | L | 0.104 | 0.006 | 169.703 |
| 1161 | Sheshbahreh et al., 2019 | 31.5 | 50.8 | Echinacea purpurea (L.) | 90 | 0.6 | POD | EA | L | 0.363 | 0.006 | 169.703 |
| 1162 | Sheshbahreh et al., 2019 | 31.5 | 50.8 | Echinacea purpurea (L.) | 90 | 0.3 | Car | NEA | L | 0.118 | 0.006 | 169.703 |
| 1163 | Sheshbahreh et al., 2019 | 31.5 | 50.8 | Echinacea purpurea (L.) | 90 | 0.6 | Car | NEA | L | 0.318 | 0.006 | 169.703 |
| 1164 | Siddiqui et al., 2016 | 25.1 | 67.3 | Halopyrum mucronatum (L.) Staph. | 7 | 0.67 | Dry weight | Growth | P | -0.663 | 0.013 | 75.676 |
| 1165 | Siddiqui et al., 2016 | 25.1 | 67.3 | Cenchrus ciliaris (L.) | 7 | 0.67 | Dry weight | Growth | P | -0.589 | 0.030 | 33.043 |
| 1166 | Siddiqui et al., 2016 | 25.1 | 67.3 | Halopyrum mucronatum (L.) Staph. | 7 | 0.67 | Chl | PS | L | -0.208 | 0.001 | 869.812 |
| 1167 | Siddiqui et al., 2016 | 25.1 | 67.3 | Cenchrus ciliaris (L.) | 7 | 0.67 | Chl | PS | L | -0.616 | 0.002 | 571.296 |
| 1168 | Siddiqui et al., 2016 | 25.1 | 67.3 | Halopyrum mucronatum (L.) Staph. | 7 | 0.67 | Chl | PS | L | -0.220 | 0.005 | 197.636 |
| 1169 | Siddiqui et al., 2016 | 25.1 | 67.3 | Cenchrus ciliaris (L.) | 7 | 0.67 | Chl | PS | L | -0.634 | 0.015 | 65.814 |
| 1170 | Siddiqui et al., 2016 | 25.1 | 67.3 | Halopyrum mucronatum (L.) Staph. | 7 | 0.67 | Fv/Fm | PS | L | -0.028 | 0.003 | 297.299 |
| 1171 | Siddiqui et al., 2016 | 25.1 | 67.3 | Cenchrus ciliaris (L.) | 7 | 0.67 | Fv/Fm | PS | L | -0.044 | 0.007 | 145.019 |
| 1172 | Siddiqui et al., 2016 | 25.1 | 67.3 | Halopyrum mucronatum (L.) Staph. | 7 | 0.67 | Proline | NEA | L | 0.509 | 0.012 | 84.447 |
| 1173 | Siddiqui et al., 2016 | 25.1 | 67.3 | Cenchrus ciliaris (L.) | 7 | 0.67 | Proline | NEA | L | 0.444 | 0.006 | 166.802 |
| 1174 | Siddiqui et al., 2016 | 25.1 | 67.3 | Halopyrum mucronatum (L.) Staph. | 7 | 0.67 | ROS | PMP | L | 0.047 | 0.001 | 995.341 |
| 1175 | Siddiqui et al., 2016 | 25.1 | 67.3 | Cenchrus ciliaris (L.) | 7 | 0.67 | ROS | PMP | L | 0.276 | 0.002 | 429.734 |
| 1176 | Silva et al., 2010 | -18.7 | -53.6 | Jatropha curcas L. | 5 | 0.53 | EL | PMP | L | 0.098 | 0.001 | 733.329 |
| 1177 | Silva et al., 2010 | -18.7 | -53.6 | Jatropha curcas L. | 5 | 0.53 | CAT | EA | L | -1.042 | 0.004 | 232.211 |
| 1178 | Silva et al., 2010 | -18.7 | -53.6 | Jatropha curcas L. | 5 | 0.53 | APX | EA | L | 0.714 | 0.007 | 135.614 |
| 1179 | Silva et al., 2010 | -18.7 | -53.6 | Jatropha curcas L. | 5 | 0.53 | SOD | EA | L | -0.094 | 0.002 | 617.374 |
| 1180 | Silva et al., 2010 | -18.7 | -53.6 | Jatropha curcas L. | 5 | 0.53 | ROS | EA | L | 0.394 | 0.001 | 1255.039 |
| 1181 | Sperdouli et al., 2012 | 49.9 | -97.1 | Arabidopsis thaliana | 1 | 0.05 | MDA | PMP | L | 0.041 | 0.002 | 604.144 |
| 1182 | Sperdouli et al., 2012 | 49.9 | -97.1 | Arabidopsis thaliana | 6 | 0.35 | MDA | PMP | L | 0.178 | 0.001 | 676.653 |
| 1183 | Sperdouli et al., 2012 | 49.9 | -97.1 | Arabidopsis thaliana | 10 | 0.5 | MDA | PMP | L | 0.045 | 0.002 | 643.474 |
| 1184 | Sperdouli et al., 2012 | 49.9 | -97.1 | Arabidopsis thaliana | 1 | 0.05 | Fv/Fm | PS | L | -0.025 | 0.001 | 1406.671 |
| 1185 | Sperdouli et al., 2012 | 49.9 | -97.1 | Arabidopsis thaliana | 6 | 0.35 | Fv/Fm | PS | L | -0.105 | 0.001 | 844.988 |
| 1186 | Sperdouli et al., 2012 | 49.9 | -97.1 | Arabidopsis thaliana | 10 | 0.5 | Fv/Fm | PS | L | -0.078 | 0.001 | 1949.260 |
| 1187 | Sperdouli et al., 2012 | 49.9 | -97.1 | Arabidopsis thaliana | 1 | 0.05 | qP | PS | L | -0.232 | 0.009 | 115.351 |
| 1188 | Sperdouli et al., 2012 | 49.9 | -97.1 | Arabidopsis thaliana | 6 | 0.35 | qP | PS | L | -0.842 | 0.069 | 14.480 |
| 1189 | Sperdouli et al., 2012 | 49.9 | -97.1 | Arabidopsis thaliana | 10 | 0.5 | qP | PS | L | -0.169 | 0.016 | 60.934 |
| 1190 | Sperdouli et al., 2012 | 49.9 | -97.1 | Arabidopsis thaliana | 1 | 0.05 | Proline | NEA | L | 0.129 | 0.016 | 62.557 |
| 1191 | Sperdouli et al., 2012 | 49.9 | -97.1 | Arabidopsis thaliana | 6 | 0.35 | Proline | NEA | L | 1.372 | 0.014 | 70.204 |
| 1192 | Sperdouli et al., 2012 | 49.9 | -97.1 | Arabidopsis thaliana | 10 | 0.5 | Proline | NEA | L | 1.857 | 0.018 | 56.623 |
| 1193 | Sperdouli et al., 2012 | 49.9 | -97.1 | Arabidopsis thaliana | 1 | 0.05 | Soluble sugar | NEA | L | 0.052 | 0.013 | 79.840 |
| 1194 | Sperdouli et al., 2012 | 49.9 | -97.1 | Arabidopsis thaliana | 6 | 0.35 | Soluble sugar | NEA | L | 0.394 | 0.010 | 100.126 |
| 1195 | Sperdouli et al., 2012 | 49.9 | -97.1 | Arabidopsis thaliana | 10 | 0.5 | Soluble sugar | NEA | L | 0.504 | 0.010 | 101.138 |
| 1196 | Sun et al., 2015 | 34.3 | 117.2 | Toyonoka Fragaria ananassa | 2 | 0.290323 | Proline | NEA | L | 0.064 | 0.003 | 361.298 |
| 1197 | Sun et al., 2015 | 34.3 | 117.2 | Toyonoka Fragaria ananassa | 6 | 0.290323 | Proline | NEA | L | 0.100 | 0.003 | 313.827 |
| 1198 | Sun et al., 2015 | 34.3 | 117.2 | Toyonoka Fragaria ananassa | 10 | 0.290323 | Proline | NEA | L | 0.249 | 0.003 | 346.208 |
| 1199 | Sun et al., 2015 | 34.3 | 117.2 | Toyonoka Fragaria ananassa | 2 | 0.42 | Proline | NEA | L | 0.111 | 0.003 | 314.765 |
| 1200 | Sun et al., 2015 | 34.3 | 117.2 | Toyonoka Fragaria ananassa | 6 | 0.42 | Proline | NEA | L | 0.348 | 0.003 | 357.642 |
| 1201 | Sun et al., 2015 | 34.3 | 117.2 | Toyonoka Fragaria ananassa | 10 | 0.42 | Proline | NEA | L | 0.610 | 0.002 | 441.247 |
| 1202 | Sun et al., 2015 | 34.3 | 117.2 | Toyonoka Fragaria ananassa | 2 | 0.55 | Proline | NEA | L | 0.271 | 0.003 | 358.873 |
| 1203 | Sun et al., 2015 | 34.3 | 117.2 | Toyonoka Fragaria ananassa | 6 | 0.55 | Proline | NEA | L | 0.699 | 0.002 | 437.030 |
| 1204 | Sun et al., 2015 | 34.3 | 117.2 | Toyonoka Fragaria ananassa | 10 | 0.55 | Proline | NEA | L | 0.799 | 0.002 | 463.317 |
| 1205 | Sun et al., 2015 | 34.3 | 117.2 | Toyonoka Fragaria ananassa | 2 | 0.290323 | Soluble sugar | NEA | L | 0.057 | 0.004 | 254.993 |
| 1206 | Sun et al., 2015 | 34.3 | 117.2 | Toyonoka Fragaria ananassa | 6 | 0.290323 | Soluble sugar | NEA | L | 0.229 | 0.003 | 332.458 |
| 1207 | Sun et al., 2015 | 34.3 | 117.2 | Toyonoka Fragaria ananassa | 10 | 0.290323 | Soluble sugar | NEA | L | 0.417 | 0.003 | 394.072 |
| 1208 | Sun et al., 2015 | 34.3 | 117.2 | Toyonoka Fragaria ananassa | 2 | 0.42 | Soluble sugar | NEA | L | 0.172 | 0.003 | 300.760 |
| 1209 | Sun et al., 2015 | 34.3 | 117.2 | Toyonoka Fragaria ananassa | 6 | 0.42 | Soluble sugar | NEA | L | 0.627 | 0.002 | 483.118 |
| 1210 | Sun et al., 2015 | 34.3 | 117.2 | Toyonoka Fragaria ananassa | 10 | 0.42 | Soluble sugar | NEA | L | 0.699 | 0.002 | 503.674 |
| 1211 | Sun et al., 2015 | 34.3 | 117.2 | Toyonoka Fragaria ananassa | 2 | 0.55 | Soluble sugar | NEA | L | 0.225 | 0.004 | 282.577 |
| 1212 | Sun et al., 2015 | 34.3 | 117.2 | Toyonoka Fragaria ananassa | 6 | 0.55 | Soluble sugar | NEA | L | 0.681 | 0.002 | 516.194 |
| 1213 | Sun et al., 2015 | 34.3 | 117.2 | Toyonoka Fragaria ananassa | 10 | 0.55 | Soluble sugar | NEA | L | 0.873 | 0.002 | 501.791 |
| 1214 | Sun et al., 2015 | 34.3 | 117.2 | Toyonoka Fragaria ananassa | 2 | 0.290323 | MDA | PMP | L | 0.012 | 0.003 | 305.439 |
| 1215 | Sun et al., 2015 | 34.3 | 117.2 | Toyonoka Fragaria ananassa | 6 | 0.290323 | MDA | PMP | L | 0.143 | 0.003 | 320.201 |
| 1216 | Sun et al., 2015 | 34.3 | 117.2 | Toyonoka Fragaria ananassa | 10 | 0.290323 | MDA | PMP | L | 0.260 | 0.003 | 377.694 |
| 1217 | Sun et al., 2015 | 34.3 | 117.2 | Toyonoka Fragaria ananassa | 2 | 0.42 | MDA | PMP | L | 0.070 | 0.003 | 309.614 |
| 1218 | Sun et al., 2015 | 34.3 | 117.2 | Toyonoka Fragaria ananassa | 6 | 0.42 | MDA | PMP | L | 0.334 | 0.003 | 369.350 |
| 1219 | Sun et al., 2015 | 34.3 | 117.2 | Toyonoka Fragaria ananassa | 10 | 0.42 | MDA | PMP | L | 0.604 | 0.002 | 544.012 |
| 1220 | Sun et al., 2015 | 34.3 | 117.2 | Toyonoka Fragaria ananassa | 2 | 0.55 | MDA | PMP | L | 0.192 | 0.002 | 402.725 |
| 1221 | Sun et al., 2015 | 34.3 | 117.2 | Toyonoka Fragaria ananassa | 6 | 0.55 | MDA | PMP | L | 0.621 | 0.002 | 443.044 |
| 1222 | Sun et al., 2015 | 34.3 | 117.2 | Toyonoka Fragaria ananassa | 10 | 0.55 | MDA | PMP | L | 0.825 | 0.002 | 569.779 |
| 1223 | Sun et al., 2015 | 34.3 | 117.2 | Toyonoka Fragaria ananassa | 2 | 0.290323 | SOD | EA | L | 0.038 | 0.005 | 205.487 |
| 1224 | Sun et al., 2015 | 34.3 | 117.2 | Toyonoka Fragaria ananassa | 6 | 0.290323 | SOD | EA | L | 0.335 | 0.004 | 250.516 |
| 1225 | Sun et al., 2015 | 34.3 | 117.2 | Toyonoka Fragaria ananassa | 10 | 0.290323 | SOD | EA | L | 0.464 | 0.004 | 281.221 |
| 1226 | Sun et al., 2015 | 34.3 | 117.2 | Toyonoka Fragaria ananassa | 2 | 0.42 | SOD | EA | L | 0.246 | 0.004 | 242.296 |
| 1227 | Sun et al., 2015 | 34.3 | 117.2 | Toyonoka Fragaria ananassa | 6 | 0.42 | SOD | EA | L | 0.805 | 0.003 | 333.333 |
| 1228 | Sun et al., 2015 | 34.3 | 117.2 | Toyonoka Fragaria ananassa | 10 | 0.42 | SOD | EA | L | 0.685 | 0.003 | 315.248 |
| 1229 | Sun et al., 2015 | 34.3 | 117.2 | Toyonoka Fragaria ananassa | 2 | 0.55 | SOD | EA | L | 0.358 | 0.004 | 266.985 |
| 1230 | Sun et al., 2015 | 34.3 | 117.2 | Toyonoka Fragaria ananassa | 6 | 0.55 | SOD | EA | L | 0.960 | 0.003 | 347.209 |
| 1231 | Sun et al., 2015 | 34.3 | 117.2 | Toyonoka Fragaria ananassa | 10 | 0.55 | SOD | EA | L | 0.658 | 0.003 | 312.785 |
| 1232 | Sun et al., 2015 | 34.3 | 117.2 | Toyonoka Fragaria ananassa | 2 | 0.290323 | POD | EA | L | 0.038 | 0.003 | 359.311 |
| 1233 | Sun et al., 2015 | 34.3 | 117.2 | Toyonoka Fragaria ananassa | 6 | 0.290323 | POD | EA | L | 0.430 | 0.002 | 472.928 |
| 1234 | Sun et al., 2015 | 34.3 | 117.2 | Toyonoka Fragaria ananassa | 10 | 0.290323 | POD | EA | L | 0.586 | 0.002 | 527.616 |
| 1235 | Sun et al., 2015 | 34.3 | 117.2 | Toyonoka Fragaria ananassa | 2 | 0.42 | POD | EA | L | 0.206 | 0.002 | 409.561 |
| 1236 | Sun et al., 2015 | 34.3 | 117.2 | Toyonoka Fragaria ananassa | 6 | 0.42 | POD | EA | L | 0.778 | 0.002 | 550.350 |
| 1237 | Sun et al., 2015 | 34.3 | 117.2 | Toyonoka Fragaria ananassa | 10 | 0.42 | POD | EA | L | 0.640 | 0.002 | 530.083 |
| 1238 | Sun et al., 2015 | 34.3 | 117.2 | Toyonoka Fragaria ananassa | 2 | 0.55 | POD | EA | L | 0.413 | 0.002 | 479.546 |
| 1239 | Sun et al., 2015 | 34.3 | 117.2 | Toyonoka Fragaria ananassa | 6 | 0.55 | POD | EA | L | 0.945 | 0.002 | 585.574 |
| 1240 | Sun et al., 2015 | 34.3 | 117.2 | Toyonoka Fragaria ananassa | 10 | 0.55 | POD | EA | L | 0.327 | 0.002 | 449.356 |
| 1241 | Sun et al., 2015 | 34.3 | 117.2 | Toyonoka Fragaria ananassa | 2 | 0.290323 | CAT | EA | L | 0.263 | 0.002 | 551.014 |
| 1242 | Sun et al., 2015 | 34.3 | 117.2 | Toyonoka Fragaria ananassa | 6 | 0.290323 | CAT | EA | L | 1.084 | 0.001 | 850.090 |
| 1243 | Sun et al., 2015 | 34.3 | 117.2 | Toyonoka Fragaria ananassa | 10 | 0.290323 | CAT | EA | L | 0.219 | 0.002 | 546.871 |
| 1244 | Sun et al., 2015 | 34.3 | 117.2 | Toyonoka Fragaria ananassa | 2 | 0.42 | CAT | EA | L | 0.514 | 0.001 | 672.282 |
| 1245 | Sun et al., 2015 | 34.3 | 117.2 | Toyonoka Fragaria ananassa | 6 | 0.42 | CAT | EA | L | 1.004 | 0.001 | 847.987 |
| 1246 | Sun et al., 2015 | 34.3 | 117.2 | Toyonoka Fragaria ananassa | 10 | 0.42 | CAT | EA | L | 0.523 | 0.002 | 666.123 |
| 1247 | Sun et al., 2015 | 34.3 | 117.2 | Toyonoka Fragaria ananassa | 2 | 0.55 | CAT | EA | L | 0.394 | 0.002 | 623.594 |
| 1248 | Sun et al., 2015 | 34.3 | 117.2 | Toyonoka Fragaria ananassa | 6 | 0.55 | CAT | EA | L | 0.400 | 0.002 | 625.497 |
| 1249 | Sun et al., 2015 | 34.3 | 117.2 | Toyonoka Fragaria ananassa | 10 | 0.55 | CAT | EA | L | -0.274 | 0.003 | 293.727 |
| 1250 | Vaseva et al., 2012 | 42.7 | 23.3 | Trifolium pratense L. | 14 | 0.7 | EL | PMP | L | 1.833 | 0.156 | 6.402 |
| 1251 | Vaseva et al., 2012 | 42.7 | 23.3 | Trifolium repens L. | 14 | 0.7 | EL | PMP | L | 2.044 | 0.077 | 12.966 |
| 1252 | Vaseva et al., 2012 | 42.7 | 23.3 | Trifolium repens L. | 14 | 0.7 | EL | PMP | L | 1.188 | 0.002 | 447.771 |
| 1253 | Vaseva et al., 2012 | 42.7 | 23.3 | Trifolium pratense L. | 14 | 0.7 | MDA | PMP | L | 1.688 | 0.041 | 24.614 |
| 1254 | Vaseva et al., 2012 | 42.7 | 23.3 | Trifolium repens L. | 14 | 0.7 | MDA | PMP | L | 0.135 | 0.011 | 93.340 |
| 1255 | Vaseva et al., 2012 | 42.7 | 23.3 | Trifolium repens L. | 14 | 0.7 | MDA | PMP | L | 0.465 | 0.014 | 72.775 |
| 1256 | Vaseva et al., 2012 | 42.7 | 23.3 | Trifolium pratense L. | 14 | 0.7 | ROS | PMP | L | 1.578 | 0.016 | 61.558 |
| 1257 | Vaseva et al., 2012 | 42.7 | 23.3 | Trifolium repens L. | 14 | 0.7 | ROS | PMP | L | 1.734 | 0.005 | 221.570 |
| 1258 | Vaseva et al., 2012 | 42.7 | 23.3 | Trifolium repens L. | 14 | 0.7 | ROS | PMP | L | 1.825 | 0.007 | 146.619 |
| 1259 | Vaseva et al., 2012 | 42.7 | 23.3 | Trifolium pratense L. | 14 | 0.7 | Proline | NEA | L | 4.327 | 0.009 | 106.071 |
| 1260 | Vaseva et al., 2012 | 42.7 | 23.3 | Trifolium repens L. | 14 | 0.7 | Proline | NEA | L | 4.503 | 0.005 | 186.417 |
| 1261 | Vaseva et al., 2012 | 42.7 | 23.3 | Trifolium repens L. | 14 | 0.7 | Proline | NEA | L | 2.843 | 0.004 | 250.818 |
| 1262 | Yactayo et al., 2013 | -12.1 | -77 | Solanum tuberosum L | 14 | 0.5 | Chl | PS | L | 0.041 | 0.004 | 226.271 |
| 1263 | Yactayo et al., 2013 | -12.1 | -77 | Solanum tuberosum L | 30 | 0.5 | Chl | PS | L | 0.045 | 0.004 | 226.271 |
| 1264 | Yactayo et al., 2013 | -12.1 | -77 | Solanum tuberosum L | 40 | 0.5 | Chl | PS | L | 0.046 | 0.004 | 226.271 |
| 1265 | Yactayo et al., 2013 | -12.1 | -77 | Solanum tuberosum L | 65 | 0.5 | Chl | PS | L | 0.267 | 0.004 | 226.271 |
| 1266 | Yang et al., 2009 | 32.1 | 118.8 | Malus sieversii | 7 | 0.333333 | EL | PMP | L | 0.617 | 0.009 | 115.317 |
| 1267 | Yang et al., 2009 | 32.1 | 118.8 | Malus sieversii | 14 | 0.333333 | EL | PMP | L | 0.643 | 0.004 | 258.512 |
| 1268 | Yang et al., 2009 | 32.1 | 118.8 | Malus sieversii | 21 | 0.333333 | EL | PMP | L | 0.758 | 0.006 | 173.357 |
| 1269 | Yang et al., 2009 | 32.1 | 118.8 | Malus sieversii | 7 | 0.333333 | MDA | PMP | L | 0.464 | 0.042 | 23.566 |
| 1270 | Yang et al., 2009 | 32.1 | 118.8 | Malus sieversii | 14 | 0.333333 | MDA | PMP | L | 0.858 | 0.047 | 21.112 |
| 1271 | Yang et al., 2009 | 32.1 | 118.8 | Malus sieversii | 21 | 0.333333 | MDA | PMP | L | 0.571 | 0.027 | 37.649 |
| 1272 | Yang et al., 2009 | 32.1 | 118.8 | Malus sieversii | 7 | 0.333333 | SOD | EA | L | -0.406 | 0.001 | 767.864 |
| 1273 | Yang et al., 2009 | 32.1 | 118.8 | Malus sieversii | 14 | 0.333333 | SOD | EA | L | -0.599 | 0.022 | 45.241 |
| 1274 | Yang et al., 2009 | 32.1 | 118.8 | Malus sieversii | 21 | 0.333333 | SOD | EA | L | -1.194 | 0.005 | 200.826 |
| 1275 | Yang et al., 2009 | 32.1 | 118.8 | Malus sieversii | 7 | 0.333333 | CAT | EA | L | 1.624 | 0.034 | 29.347 |
| 1276 | Yang et al., 2009 | 32.1 | 118.8 | Malus sieversii | 14 | 0.333333 | CAT | EA | L | 0.161 | 0.026 | 38.085 |
| 1277 | Yang et al., 2009 | 32.1 | 118.8 | Malus sieversii | 21 | 0.333333 | CAT | EA | L | 0.335 | 0.043 | 23.119 |
| 1278 | Zhang et al., 2013 | 34.5 | 108.1 | Canna edulis Ker. | 7 | 0.4 | Chl | PS | L | 0.033 | 0.008 | 131.885 |
| 1279 | Zhang et al., 2013 | 34.5 | 108.1 | Canna edulis Ker. | 21 | 0.4 | Chl | PS | L | -0.022 | 0.017 | 60.597 |
| 1280 | Zhang et al., 2013 | 34.5 | 108.1 | Canna edulis Ker. | 35 | 0.4 | Chl | PS | L | -0.265 | 0.006 | 177.981 |
| 1281 | Zhang et al., 2013 | 34.5 | 108.1 | Canna edulis Ker. | 7 | 0.4 | Chl | PS | L | -0.087 | 0.005 | 197.253 |
| 1282 | Zhang et al., 2013 | 34.5 | 108.1 | Canna edulis Ker. | 21 | 0.4 | Chl | PS | L | -0.095 | 0.014 | 69.674 |
| 1283 | Zhang et al., 2013 | 34.5 | 108.1 | Canna edulis Ker. | 35 | 0.4 | Chl | PS | L | 0.118 | 0.009 | 107.255 |
| 1284 | Zhang et al., 2013 | 34.5 | 108.1 | Canna edulis Ker. | 7 | 0.4 | MDA | PMP | L | 0.004 | 0.034 | 29.380 |
| 1285 | Zhang et al., 2013 | 34.5 | 108.1 | Canna edulis Ker. | 21 | 0.4 | MDA | PMP | L | -0.257 | 0.058 | 17.160 |
| 1286 | Zhang et al., 2013 | 34.5 | 108.1 | Canna edulis Ker. | 35 | 0.4 | MDA | PMP | L | 0.367 | 0.004 | 231.186 |
| 1287 | Zhang et al., 2013 | 34.5 | 108.1 | Canna edulis Ker. | 7 | 0.4 | SOD | EA | L | -0.278 | 0.015 | 65.789 |
| 1288 | Zhang et al., 2013 | 34.5 | 108.1 | Canna edulis Ker. | 21 | 0.4 | SOD | EA | L | -0.232 | 0.004 | 236.740 |
| 1289 | Zhang et al., 2013 | 34.5 | 108.1 | Canna edulis Ker. | 35 | 0.4 | SOD | EA | L | -0.535 | 0.007 | 137.616 |
| 1290 | Zhang et al., 2013 | 34.5 | 108.1 | Canna edulis Ker. | 7 | 0.4 | CAT | EA | L | 0.545 | 0.014 | 73.189 |
| 1291 | Zhang et al., 2013 | 34.5 | 108.1 | Canna edulis Ker. | 21 | 0.4 | CAT | EA | L | -0.772 | 0.032 | 31.168 |
| 1292 | Zhang et al., 2013 | 34.5 | 108.1 | Canna edulis Ker. | 35 | 0.4 | CAT | EA | L | -0.742 | 0.027 | 36.865 |
| 1293 | Zhang et al., 2013 | 34.5 | 108.1 | Canna edulis Ker. | 7 | 0.4 | POD | EA | L | -0.030 | 0.013 | 79.269 |
| 1294 | Zhang et al., 2013 | 34.5 | 108.1 | Canna edulis Ker. | 21 | 0.4 | POD | EA | L | 0.034 | 0.000 | 2612.711 |
| 1295 | Zhang et al., 2013 | 34.5 | 108.1 | Canna edulis Ker. | 35 | 0.4 | POD | EA | L | -1.236 | 0.017 | 57.511 |
| 1296 | Zhang et al., 2019 | 39.8 | 116.3 | Zea mays L. | 3 | 0.42 | Dry weight | Growth | L | -0.147 | 0.005 | 197.990 |
| 1297 | Zhang et al., 2019 | 39.8 | 116.3 | Zea mays L. | 6 | 0.53 | Dry weight | Growth | L | -0.288 | 0.009 | 111.483 |
| 1298 | Zhang et al., 2019 | 39.8 | 116.3 | Zea mays L. | 3 | 0.42 | Fv/Fm | PS | L | -0.143 | 0.003 | 289.530 |
| 1299 | Zhang et al., 2019 | 39.8 | 116.3 | Zea mays L. | 6 | 0.53 | Fv/Fm | PS | L | -0.216 | 0.007 | 139.467 |
| 1300 | Zhang et al., 2019 | 39.8 | 116.3 | Zea mays L. | 3 | 0.42 | ABA | NEA | L | 0.772 | 0.003 | 383.929 |
| 1301 | Zhang et al., 2019 | 39.8 | 116.3 | Zea mays L. | 6 | 0.53 | ABA | NEA | L | 0.139 | 0.015 | 66.741 |

**Table S3.** The values of Akaike information criterion for five alternative models of each physiological indices (see Methods).

| Physiological  indices | Model 1 | Model 2 | Model 3 | Model 4* | Model 5 |
| --- | --- | --- | --- | --- | --- |
| ABA | 52.19 | 51.06 | 51.33 | 52.47 | 54.02 |
| APX | 130.36 | 133.81 | 133.79 | 130.26 | 133.17 |
| AsA | 19.92 | 18.04 | 18.57 | 20.41 | 22.95 |
| Carotenoid | 93.86 | 93.57 | 92.85 | 93.28 | 96.89 |
| CAT | 283.73 | 282.67 | 281.82 | 282.78 | 289.12 |
| Chl | 122.88 | 131.31 | 131.06 | 122.39 | 126.77 |
| Dry Weight | 342.87 | 343.91 | 343.66 | 342.68 | 348.14 |
| EL | 45.91 | 48.67 | 48.60 | 45.76 | 50.55 |
| Fv/Fm | 3.27 | 3.77 | 3.99 | 3.03 | 11.73 |
| GR | 68.01 | 68.31 | 68.28 | 67.97 | 73.81 |
| MDA | 211.47 | 217.10 | 217.98 | 213.56 | 219.46 |
| POD | 170.24 | 169.60 | 169.68 | 170.29 | 174.86 |
| Proline | 199.95 | 199.28 | 198.05 | 198.70 | 204.07 |
| Protein | 42.20 | 48.52 | 48.67 | 42.32 | 47.58 |
| qP | 20.87 | 18.24 | 17.03 | 19.85 | 25.83 |
| ROS | 78.59 | 77.63 | 78.48 | 79.33 | 83.80 |
| SOD | 276.84 | 283.06 | 280.47 | 274.23 | 279.46 |
| Soluble sugar | 39.45 | 39.04 | 41.65 | 42.14 | 35.70 |

Model 1:$\ln RR=\beta_{0}+\beta_{1}\cdot DI+\beta_{2}\cdot ln (ED)+\pi_{study}+\varepsilon$

Model 2: $\ln RR=\beta_{0}+\beta_{1}\cdot DI+\beta_{2}\cdot ED+\pi_{study}+\varepsilon$

Model 3: $\ln RR=\beta_{0}+\beta_{1}\cdot\ln(DI)+\beta_{2}\cdot ED+\pi_{study}+\varepsilon$

Model 4: $\ln RR=\beta_{0}+\beta_{1}\cdot\ln(DI)+\beta_{2}\cdot\ln(ED)+\pi_{study}+\varepsilon$

Model5:$\ln RR=\beta_{0}+\beta_{1}\cdot DI+\beta_{2}\cdot\ln(ED)+\beta_{3}\cdot ln(DI)\times\ln(ED)+\pi_{study}+\varepsilon$
